# Multivariate network meta-analysis incorporating class effects

**DOI:** 10.1186/s12874-020-01025-8

**Published:** 2020-07-08

**Authors:** Rhiannon K. Owen, Sylwia Bujkiewicz, Douglas G. Tincello, Keith R. Abrams

**Affiliations:** Department of Health Sciences, University of Leicester, University Road, Leicester, LE1 7RH UK

**Keywords:** Multivariate, Network meta-analysis, Mixed treatment comparisons, Meta-analysis, Class effect

## Abstract

**Background:**

Network meta-analysis synthesises data from a number of clinical trials in order to assess the comparative efficacy of multiple healthcare interventions in similar patient populations. In situations where clinical trial data are heterogeneously reported i.e. data are missing for one or more outcomes of interest, synthesising such data can lead to disconnected networks of evidence, increased uncertainty, and potentially biased estimates which can have severe implications for decision-making. To overcome this issue, strength can be borrowed between outcomes of interest in multivariate network meta-analyses. Furthermore, in situations where there are relatively few trials informing each treatment comparison, there is a potential issue with the sparsity of data in the treatment networks, which can lead to substantial parameter uncertainty. A multivariate network meta-analysis approach can be further extended to borrow strength between interventions of the same class using hierarchical models.

**Methods:**

We extend the trivariate network meta-analysis model to incorporate the exchangeability between treatment effects belonging to the same class of intervention to increase precision in treatment effect estimates. We further incorporate a missing data framework to estimate uncertainty in trials that did not report measures of variability in order to maximise the use of all available information for healthcare decision-making. The methods are applied to a motivating dataset in overactive bladder syndrome. The outcomes of interest were mean change from baseline in incontinence, voiding and urgency episodes. All models were fitted using Bayesian Markov Chain Monte Carlo (MCMC) methods in WinBUGS.

**Results:**

All models (univariate, multivariate, and multivariate models incorporating class effects) produced similar point estimates for all treatment effects. Incorporating class effects in multivariate models often increased precision in treatment effect estimates.

**Conclusions:**

Multivariate network meta-analysis incorporating class effects allowed for the comparison of all interventions across all outcome measures to ameliorate the potential impact of outcome reporting bias, and further borrowed strength between interventions belonging to the same class of treatment to increase the precision in treatment effect estimates for healthcare policy and decision-making.

## Background

In a health technology assessment (HTA) setting, it is imperative that decisions regarding healthcare policy are formulated with consideration to all outcomes associated with the decision question, using all relevant evidence, and appropriately taking uncertainty in to account. In many healthcare conditions, the effects of new interventions are reported on multiple outcomes and these effects are correlated. Taking in to account the dependence between correlated treatment effects, as well as heterogeneous reporting of treatment effects, may have a substantial impact on the ability to appropriately estimate cost-effectiveness for new interventions [1], especially in terms of capturing uncertainty.

Network meta-analysis (NMA) is widely used in an evidence synthesis setting due to the attractive nature of utilizing all relevant information from both direct evidence (obtained from head-to-head trials) and indirect evidence (obtained from trials that share a common treatment comparator) [2–4]. Multivariate network meta-analysis (MVNMA) builds on this framework and has the ability to simultaneously model treatment effects for multiple outcomes, as well as estimate the correlation between them. Consequently, for the studies that did not report certain outcomes of interest, it is possible to obtain a predictive value for missing data by modelling the treatment effects on multiple outcomes jointly, taking into account the correlation between them. This methodology can not only increase the information for decision making, but it can also limit the potential for outcome reporting bias in the original trials [5, 6], and consequently increase precision and confidence in the treatment effect estimates [7].

There are two types of correlations that need to be incorporated in the estimation of treatment effect estimates for multiple outcomes - one at the between-study level, and another at the within-study level. Between-study correlations occur due to variability between-studies. Between-study variability occurs in situations with which studies have a different distribution of potential treatment effect modifiers, such as differences in patient characteristics, trial designs, and baseline patient severity. Within-study correlations occur between treatment effects on multiple outcomes within a trial, and are a consequence of differences in patient-level characteristics. These correlations are indicative of the association between treatment effects measured on multiple outcomes on the same patients within a study. Within-study correlations are often difficult to estimate and they are seldom reported in clinical trials. Thus, estimation is often required using individual participant data [8], for example by bootstrapping [9], or elicited from expert opinion [10].

The ability to simultaneously model treatment effects on multiple outcomes in a multivariate analysis is an appealing feature in many HTA settings, as interest frequently lies in multiple and correlated outcome measures [11]. In the last 20 years, evidence synthesis methods have witnessed a rapid increase in methodological developments and applications of multivariate analyses to assess interventions with two or more outcomes of interest [9, 11–19]. It is often desirable to account for the correlation between treatment effects on multiple outcomes in a meta-analysis framework as this has the ability to borrow strength between all reported outcomes and studies in order to inform treatment effect estimates [9]. Such an approach is commonly referred to as multivariate meta-analysis. In more recent developments, multivariate meta-analyses have been extended to incorporate multiple treatment comparisons or network meta-analyses [7, 10, 20]. Ades et al. [20] simultaneously modelled mutually exclusive, competing risk outcomes, using a multinomial likelihood whereby the within-study correlations were accounted for but the between-study correlations were assumed to be zero. Efthimiou et al. [10] proposed a MVNMA model that accounts for both the between- and within-study correlations of binary outcomes. Specifically, Efthimiou et al. [10] incorporated the within-study correlations at the study-specific treatment contrast level, which can be problematic when incorporating multi-arm trials. Achana et al. [7] used a more natural modelling approach for arm-level data whereby the within-study correlations were incorporated at the treatment-arm level. Using this approach, Achana et al. [7] considered the treatment arms to be independent as a consequence of randomisation, which greatly eases computation of the likelihood for multi-arm trials. Furthermore, Achana et al. [7] developed this methodology to borrow information across outcomes in order to predict an estimate for missing data. This methodology allows disconnected interventions to be incorporated in to the analyses if they belong to a connected network for one or more additional outcomes; thereby, allowing all interventions to be evaluated across all outcome measures.

In situations with which there are a large number of interventions in the treatment networks and relatively few trials informing each treatment comparison, there is a potential issue with sparsity of data, which can lead to substantial parameter uncertainty. Collapsing the intervention arms into their respective treatment classes, also known as “lumping" interventions, increases the evidence base and precision in the effect estimates, but with such a class-based approach, the direct interpretation of individual intervention effects (especially those of dose or formulation effects) are lost, which can hinder decision-making. To overcome this issue, exchangeability between the same interventions, but with different formulations and/or treatment regimes can be incorporated in a three-level hierarchical NMA to borrow information within the classes of interventions, strengthening inferences, and potentially reducing the uncertainty around the individual intervention effects [21].

Furthermore, in situations where clinical trial data are heterogeneously reported and/or missing, synthesising such data can be problematic. Meta-analysis methods require specification of both a measure of effect and a measure of variability in the original trial reports. In the absence of such data, trials are often excluded from the analysis [22]. In a decision making context, missing data can have a detrimental impact on the overall decision, as many of the interventions of interest have to be excluded from the analyses. For this reason, when meta-analysing data, it is good practice to estimate missing standard errors for all models, based on additional measures of uncertainty [23].

This paper extends the methodology of Achana et al. [7] to incorporate the exchangeability between interventions of the same class [21], and further accounts for correlations between baseline and follow-up for missing measures of variability. This approach makes use of all available data by borrowing strength within classes of interventions as well as across outcomes. Borrowing information in this way has the potential to increase the precision in treatment effect estimates, as well as allowing for the comparison of all interventions across all outcome measures, to aid decision making. The remainder of this article is set out as follows: the first section describes the motivating example in overactive bladder (OAB). This is followed by a description of the multivariate network meta-analysis methods, model developments, and model estimation, before applying these methods to the motivating dataset and presenting the results. The article concludes with a discussion and final remarks.

### Motivating dataset

The cardinal symptoms of OAB syndrome are urinary incontinence, increased daytime frequency (or voiding), and urgency. A gold standard tool for assessing the quantitative measure of OAB symptoms are patient-reported bladder diaries whereby individuals with OAB record the frequency and severity of OAB symptoms on a daily basis [24]. These patient-reported bladder diaries are often used as the primary outcome in trials assessing new interventions for OAB. However, many of the key symptoms are often under-reported. For example, in the current literature urgency is defined as “the cardinal symptom" of OAB [25], but there are far fewer studies evaluating interventions for urgency (n=62) compared to incontinence (n=117) and voiding episodes (n=124). This poses several limitations for decision makers; most notably, it is difficult to estimate both clinical and cost-effectiveness of interventions across the entire symptom syndrome.

In this motivating dataset, interest lies in evaluating the change from baseline for several symptomatic responses. Whilst symptoms at baseline and follow-up are commonly reported, change from baseline is often unreported; and thus, whilst it is possible to calculate the mean change from baseline, the variance is often missing. Table 1 illustrates the different scenarios for trial reporting in the included studies. In this example, there were no trials that reported follow-up data alone.

**Table 1:** Scenarios for trial reporting

	**Change from baseline**		**Baseline**		**Follow-up**	
**Scenario**	mean	variance	mean	variance	mean	variance
**1**	✓	✓	✓	✓	✓	✓
**2**	✓	✓	✓	✓	NR	NR
**3**	✓	✓	NR	NR	✓	✓
**4**	✓	✓	NR	NR	NR	NR
**5**	✓	NR	✓	✓	✓	✓
**6**	✓	NR	✓	✓	NR	NR
**7**	✓	NR	NR	NR	NR	NR

NR denotes not reported

**Fig. 1:**
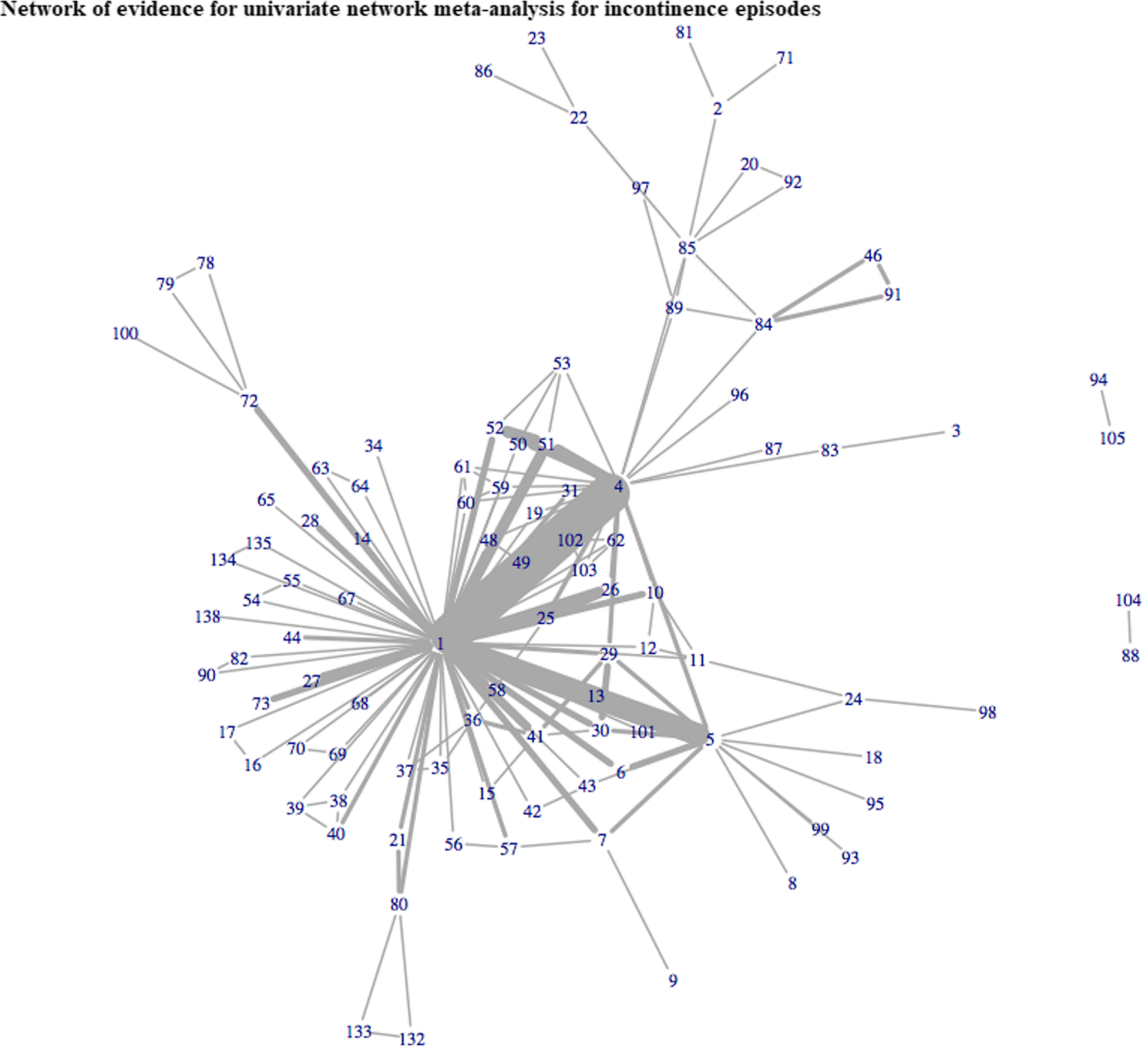
Network of evidence for univariate network meta-analysis for incontinence episodes

**Fig. 2:**
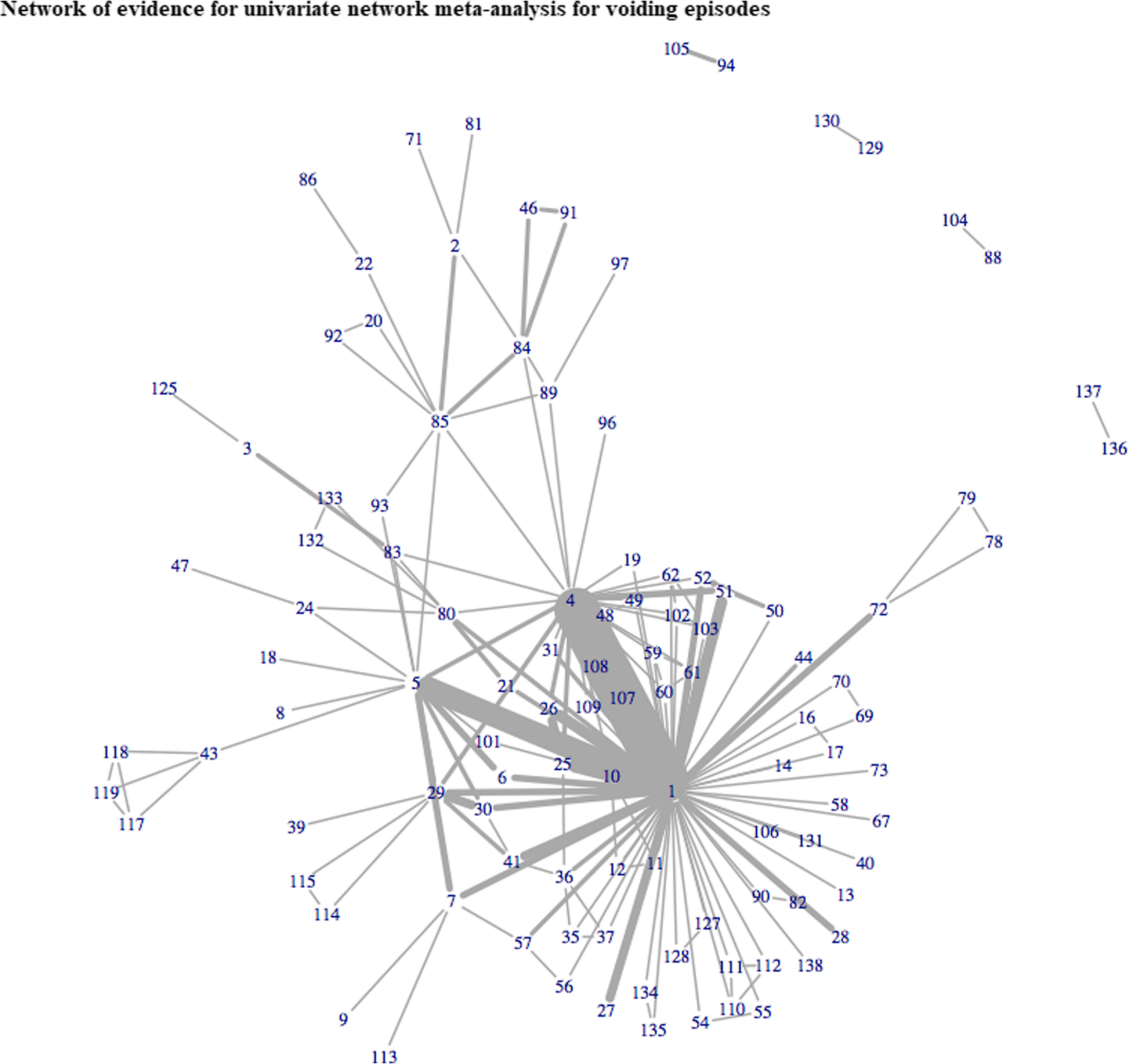
Network of evidence for univariate network meta-analysis for voiding episodes

**Fig. 3:**
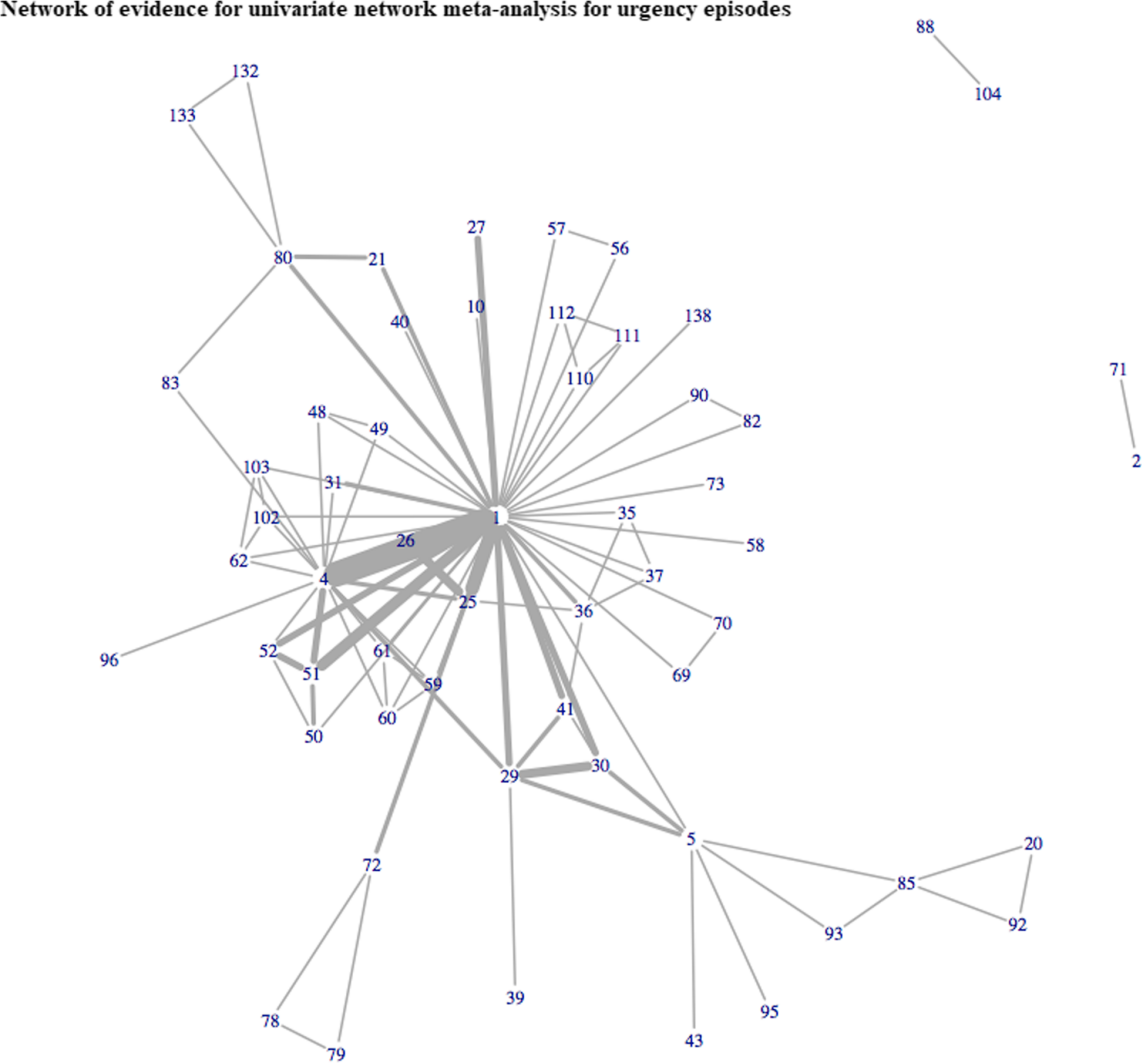
Network of evidence for univariate network meta-analysis for urgency episodes

In total, 143 studies reported one of the three cardinal symptoms of OAB. Of these, only 51 (36%) reported treatment effects for all 3 outcomes. A total of 117, 124, and 62 trials evaluated 101, 108, and 58 different interventions for incontinence, voiding, and urgency episodes, respectively. Figures 1, 2 and 3 illustrate the networks of evidence for incontinence, voiding and urgency episodes, with corresponding treatment codes given in Additional file 1. Nearly one third of all studies did not report measures of variability in the mean treatment effects. In total, 44 (38%), 42 (34%), and 18 (29%) studies solely reported mean effects and gave no measure of uncertainty or variability for incontinence, voiding, and urgency episodes, respectively.

**Fig. 4:**
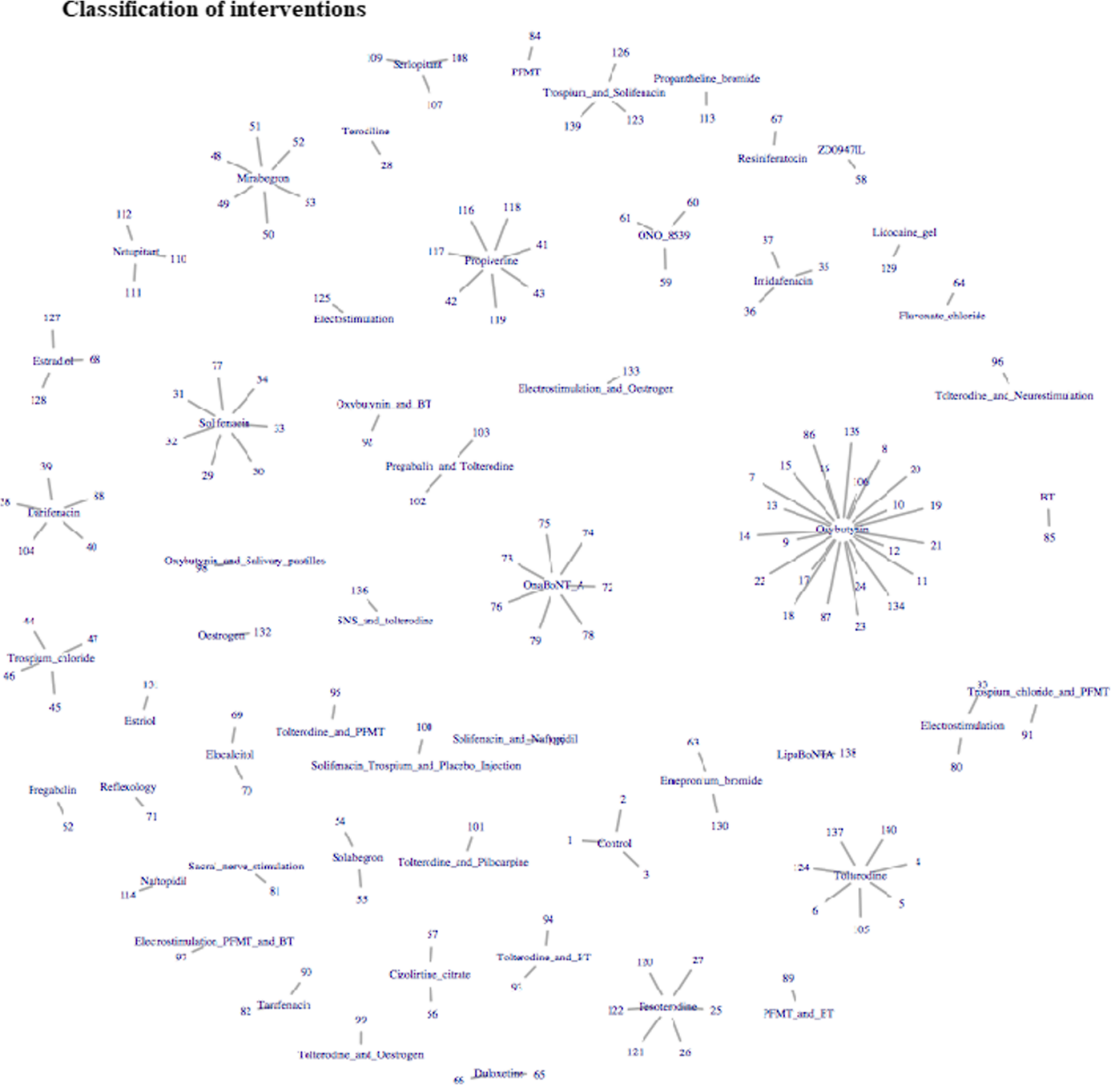
Classification of interventions

For analyses incorporating class effects, interventions were grouped according to expert clinical opinion (DGT). Figure 4 demonstrates the treatment classification of each of the individual interventions, where the central node represents the classes of treatments and the linked arms represent each of the individual interventions within those classes.

## Methods

In this section, we begin by recalling the MVNMA model described by Achana et al. [7] (Model 1) and develop this framework further to incorporate the exchangeability between interventions of the same class of interventions (Model 2). We then describe a missing data framework to incorporate studies with missing measures of uncertainty or variability.

### Model 1: multivariate network meta-analysis

Following the random effects MVNMA described by Achana et al. [7], let ***Y***_***ij***_=(*y*_*ij*1_,*y*_*ij*2_,*y*_*ij*3_), be the observed vector of effects for intervention *j* of the *i*^*th*^ study (*i*=1,2,...,*n*_*s*_) for each of the outcomes of interest (1 = urinary incontinence, 2 = voiding frequency, 3 = urgency episodes). Let ***Y***_***ij***_ follow a multivariate normal (MVN) distribution such that:

1 $$ {\begin{aligned} \boldsymbol{Y_{ij}} &\sim \textrm{MVN}(\boldsymbol{\theta_{ij}}, \boldsymbol{S_{ij}})\\ \boldsymbol{\theta_{ij}} & = \boldsymbol{\mu_{i}} + \boldsymbol{\delta_{i,bk}}I_{\lbrace j = k \rbrace} \\ \quad \text{where}~ I_{\left\lbrace u\right\rbrace}& = \left\{ \begin{array}{ll} 1 \quad \textrm{ if}\, u \, \text{is true} \\ 0 \quad \text{otherwise} \end{array} \right. \end{aligned}}  $$

where ***θ***_***ij***_ is a vector of true treatment effects, ***S***_***ij***_ is the treatment-specific within-study covariance matrix assumed known, ***μ***_***i***_ is a vector of true baseline effects in study *i* with baseline treatment *b*, and ***δ***_***i,bk***_ is a vector of treatment-specific effects in the *k*^*th*^ arm relative to the baseline treatment in arm 1 of study *i*. The elements of ***S***_***ij***_ are expressed as:

2 $$ {{}\begin{aligned} \boldsymbol{S_{ij}}= \left(\begin{array}{ccc} se_{ij(1)}^{2} & \rho_{w_{(12)}}se_{ij(1)}se_{ij(2)} & \rho_{w_{(13)}}se_{ij(1)}se_{ij(3)}\\ \rho_{w_{(12)}}se_{ij(1)}se_{ij(2)} & se_{ij(2)}^{2} & \rho_{w_{(23)}}se_{ij(2)}se_{ij(3)} \\ \rho_{w_{(13)}}se_{ij(1)}se_{ij(3)} & \rho_{w_{(23)}}se_{ij(2)}se_{ij(3)} & se_{ij(3)}^{2} \end{array}\right) \end{aligned}}  $$

where *se*_*ij*(*l*)_ denotes the observed standard errors of intervention *j* for outcome, *l*=1,2,3, and $\rho _{w_{(qr)}}$ denotes the within-study correlations, for *q*=1,2 and *r*=2,3. For 2-arm trials, the treatment effect differences of ***δ***_***i,bk***_ are assumed to be normally distributed and correlated:

3 $$ {\begin{aligned} \left(\begin{array}{l} \delta_{i,bk(1)} \\ \delta_{i,bk(2)} \\ \delta_{i,bk(3)} \end{array}\right) \sim \textrm{MVN}& \left(\left(\begin{array}{l} d_{t_{ib}t_{ik}(1)}= d_{1,t_{ik}(1)} - d_{1,t_{ib}(1)} \\ d_{t_{ib}t_{ik}(2)}= d_{1,t_{ik}(2)} - d_{1,t_{ib}(2)} \\ d_{t_{ib}t_{ik}(3)}= d_{1,t_{ik}(3)} - d_{1,t_{ib}(3)} \end{array}\right), \boldsymbol \Sigma \right) \end{aligned}}   $$

where

4 $$ \begin{aligned} \boldsymbol \Sigma = \left(\begin{array}{ccc} \sigma_{1}^{2} & \rho_{12}\sigma_{1}\sigma_{2} & \rho_{13}\sigma_{1}\sigma_{3}\\. & \sigma_{2}^{2} & \rho_{23}\sigma_{2}\sigma_{3}\\. &. & \sigma_{3}^{2} \end{array}\right) \end{aligned}  $$

where $d_{t_{ib}t_{ik}}(l)$ represents the pooled effect of the treatment in arm *k* relative to the treatment in arm *b* in study *i* for each outcome, *l*. ***Σ*** is the between-study covariance matrix under an assumption of homogeneous between-study variances [2] and correlations across treatment contrasts [7]. This notation can easily be extended to account for multi-arm trials [26], which is further described in Additional file 2.

The relative effect of the study-specific reference treatment in arm 1 (the control arm) relative to itself for outcome *l*, *δ*_*i*,*b*1(*l*)_, is set to 0 and as such the set of conditional univariate distributions begin with the relative effect of the intervention in arm 2 relative to the control arm, *δ*_*i*,*b*2(*l*)_. The study-specific treatment comparisons, *δ*_*i*,*b*(*k*−1)(*l*)_, are expressed in terms of the basic parameters of the pooled treatment effects for the intervention in arm *k*, $d_{1,t_{ik}(l)}$ and the basic parameters of the pooled treatment effects for the intervention in the reference treatment arm, $d_{1,t_{ib}(l)}$. For example, for trials that compare interventions *A* and *B* for outcome *l*, the pooled treatment effect, $d_{t_{ib}t_{ik}(l)}= d_{AB(l)}$, is given by:

5 $$ d_{t_{ib}t_{ik}(l)}= d_{AB(l)} = d_{1B(l)} - d_{1A(l)}   $$

For *d*_*AB*_ where *A*>1 and *B*>1, the relative effects are expressed in terms of the basic parameters described in Eq. (5). The intervention effect of the reference treatment for the entire treatment network, *j*=1(*l*), usually a placebo or control intervention, is set to 0 for every outcome *l*, such that *d*_11(*l*)_=0.

In order to predict treatment effect estimates for missing data for trials that did not report all outcomes of interest, following Achana et al. [7], we assumed that the pooled effects of intervention *j*=2,...,*n*_*t*_ relative to a reference treatment, *d*_1*j*(*l*)_, for outcome, *l*, can be expressed as a sum of treatment-specific effects, *α*_*j*_, and outcome-specific effect, *γ*_*l*_, such that:

6 $$ \begin{aligned} d_{1j(l)}\sim \text{Normal}(\alpha_{j}+\gamma_{l}, \zeta^{2})  \end{aligned}  $$

The parameter *ζ* indicates the deviation of treatment effect profiles across outcomes. If *ζ* was close to zero, this would indicate a high degree of similarity between outcomes. In situations where *ζ* was particularly large, this would indicate a substantial deviation between treatment effect profiles across outcomes.

Non-informative prior distributions were specified for *μ*_*il*_, *α*_*j*_∼Normal(0,10^3^), *σ*_*l*_∼Uniform(0,2), and *ζ*∼Uniform(0,2), for *l*=1,2,3. The spherical parameterisation technique based on Cholesky decomposition [27] was used to express the between-studies variance-covariance matrix, ***Σ***, and discussed in more detail in Additional file 3.

### Model 2: multivariate network meta-analysis incorporating class effects

To utilise the additional similarity between the same interventions with different regimes, Model 1 was developed further to incorporate class effects. For study *i* evaluating intervention *j* belonging to class *m*, $\boldsymbol {Y_{ij_{m}}} = (y_{ij_{m}1}\textrm {,} y_{ij_{m}2}\textrm {,} y_{ij_{m}3})$, denotes the observed vector of effects for each of the outcomes of interest, and expressed in the following way:

7 $$ \begin{aligned} \boldsymbol{Y_{ij_{m}}} \sim \textrm{MVN}(\boldsymbol{\theta_{ij_{m}}}, \boldsymbol{S_{ij_{m}}}) \\ \boldsymbol{\theta_{ij_{m}}}= \boldsymbol{\mu_{i}} + \boldsymbol{\delta_{i,bk}}I_{\lbrace j_{m} = k \rbrace} \\ \textrm{where }I_{\left\lbrace u\right\rbrace}= \left\{\begin{array}{ll} 1 \quad \textrm{ if}\, u \, \text{is true} \\ 0 \quad \text{otherwise} \end{array}\right. \end{aligned}  $$

where ***μ***_***i***_ and ***δ***_***i,bk***_ have the same interpretation as in Model 1. The parameter $\boldsymbol {\theta _{ij_{m}}}$, represents a vector of true treatment effects, and $\boldsymbol {S_{ij_{m}}}$ represents the within-study covariance matrix, for intervention *j* belonging to a broader class of interventions *m*, for study *i*, such that:

8 $$ {{}\begin{aligned} \boldsymbol{S_{ij_{m}}}= \left(\begin{array}{ccc} se_{ij_{m}(1)}^{2} & \rho_{w_{(12)}}se_{ij_{m}(1)}se_{ij_{m}(2)} & \rho_{w_{(13)}}se_{ij_{m}(1)}se_{ij_{m}(3)}\\ \rho_{w_{(12)}}se_{ij_{m}(1)}se_{ij_{m}(2)} & se_{ij_{m}(2)}^{2} & \rho_{w_{(23)}}se_{ij_{m}(2)}se_{ij_{m}(3)} \\ \rho_{w_{(13)}}se_{ij_{m}(1)}se_{ij_{m}(3)} & \rho_{w_{(23)}}se_{ij_{m}(2)}se_{ij_{m}(3)} & se_{ij_{m}(3)}^{2} \end{array}\right) \end{aligned}}  $$

As in Model 1, the elements of ***δ***_***i,bk***_ for 2-arm trials are assumed to be drawn from a multivariate normal distribution as described in Eq. 3 (and generalised to multi-arm trials as described in Additional file 2). Here ***δ***_***i,bk***_ is expressed in terms of the pooled effect, $d_{t_{ib}t_{ik}}(l)$, of the treatment in arm *k* relative to the treatment in arm *b* in study *i* for each outcome, *l*. For example, when we incorporate class effects, the pooled treatment effect of treatment *A* belonging to class *c*, relative to the pooled treatment effect of treatment *B* belonging to class *g*, $d_{A_{c}B_{g}(l)}$, is given by:

9 $$ d_{t_{ib}t_{ik}(l)}= d_{A_{c}B_{g}(l)} = d_{1_{1}B_{g}(l)} - d_{1_{1}A_{c}(l)}   $$

The intervention effect of the reference treatment for the entire treatment network, *j*=1_1_(*l*), usually a placebo or control intervention, is set to 0 for every outcome *l*, such that $d_{1_{1}1_{1}(l)}=0$. The basic parameters for relative treatment effects, $d_{1_{1}j_{m}(l)}$, of intervention *j* within class *m*, relative to the reference treatment, were assumed to follow a normal distribution with mean equal to the treatment-specific effect, $\alpha _{j_{m}}$, plus the outcome-specific effect, *γ*_*l*_, and variance, *ζ*^2^:

10 $$ d_{1_{1}j_{m}(l)}\sim \text{Normal}(\alpha_{j_{m}}+\gamma_{l}, \zeta^{2})   $$

where *γ*_*l*_ and *ζ*^2^ have the same interpretation as Model 1.

In order to incorporate the exchangeability between treatment-specific effects, $\alpha _{j_{m}}$, within class *m*, $\alpha _{j_{m}}$ was assumed to follow a normal distribution with mean equal to the pooled effect estimate for the *m*^*th*^ class of interventions, *β*_*m*_, with class specific between-intervention variance, $\nu _{m}^{2}$, [21] such that:

11 $$ \alpha_{j_{m}}\sim \text{Normal}(\beta_{m}, {\nu_{m}}^{2})   $$

Non-informative prior distributions were specified for *μ*_*il*_, *σ*_*l*_∼Uniform(0,2) for *l*=1,2,3, *ζ*∼Uniform(0,2), *β*_*m*_∼Normal(0,10^3^) and *ν*_*m*_∼Uniform(0,2). The spherical parameterisation technique based on Cholesky decomposition [27] was used to construct a prior distribution for the between-study variance-covariance matrix, ***Σ***, and discussed in more detail in Additional file 3.

In all multivariate analyses, prior distributions for the variance parameters (i.e. *σ*_*l*_, *ζ*, and *ν*_*m*_) were restricted to a Uniform(0,2) distribution on the standard deviation scale. For example, a value of 2 for the class-specific between-intervention variance, *ν*_*m*_, suggests that for a random pair of interventions, the difference in the mean change from baseline could be as large as 2.2 events on average. Uniform(0,2) prior distributions were considered for variance components in multivariate analyses in order to aid computation of the variance-covariance matrix. However, there is an argument to suggest that the variance parameters in a Bayesian model can be decomposed into the sum of several other variance components [28]. Thus, in hierarchical NMAs with additional variance components at the class-level, more informative prior distributions for variance parameters may be reasonable.

### Missing data framework

In situations where the observed standard errors of treatment effects, *se*_*ij*_, were not reported, but baseline and follow-up variances were available (scenario 5 of Table 1), the correlation, *ξ*, between variance at baseline, ${sd_{\text {baseline}_{ij}}}^{2}$, and follow-up, ${sd_{\text {followup}_{ij}}}^{2}$, were used to impute estimates of the variance for change from baseline, ${sd_{\text {change}_{ij}}}^{2}$. This is calculated as:

12 $$ \begin{aligned} {sd_{\text{change}_{ij}}}^{2} &= {sd_{\text{baseline}_{ij}}}^{2} + {sd_{\text{followup}_{ij}}}^{2} \\&- 2\xi({sd_{\text{baseline}_{ij}}} \times {sd_{\text{followup}_{ij}}}) & \\ &{se_{ij}}^{2} = \frac{{sd_{\text{change}_{ij}}}^{2}}{\sqrt{n_{ij}}} \end{aligned}  $$

Using external information from trials that report all variance terms (scenario 1 of Table 1), an informative prior distribution was placed on the correlation, *ξ*, using Fisher’s Z-transformation [23]. For trials that do not report the variability at follow-up (scenario 6 of Table 1), a linear predictor with baseline variance as a covariate was included such that:

13 $$ sd_{\text{followup}_{ij}} = \upsilon + \lambda (sd_{\text{baseline}_{ij}})  $$

where *υ* represents a constant term, and *λ* the regression coefficient. Trials that did not provide a measure of variability (scenario 7 of Table 1) were excluded from the analyses.

### Model estimation

All models were estimated using Markov Chain Monte Carlo implemented in WinBUGS 1.4.3 [29]. Example WinBUGS code for the MVNMA incorporating class effects and the missing data framework is given in Additional file 4. Samples were collected for 150,000 MCMC iterations with the first 10,000 iterations discarded in the form of a ‘burn-in.’ Convergence plots were assessed for a random sample of parameters of interest including treatment effect estimates and between-study standard deviations [30]. Brooks-Gelman-Rubin statistics, autocorrelation, history, and trace plots were used to detect non-convergence for three individual MCMC chains with disparate starting values. Sensitivity analyses were undertaken to assess the impact of the choice of prior distributions for variance parameter. For both variance parameters, two alternative distributions were considered. For prior specification of the elements of ***V***^***1/2***^, (1) Gamma(0.001,0.001) on the precision scale, and (2) Half-normal(0,1) on the standard deviation scale were considered. For prior specification of *ζ*, (1) Gamma(0.01,0.01) on the precision scale, and (2) Half-normal(0,1) on the standard deviation scale were considered.

## Results

### Multivariate network meta-analysis

**Fig. 5:**
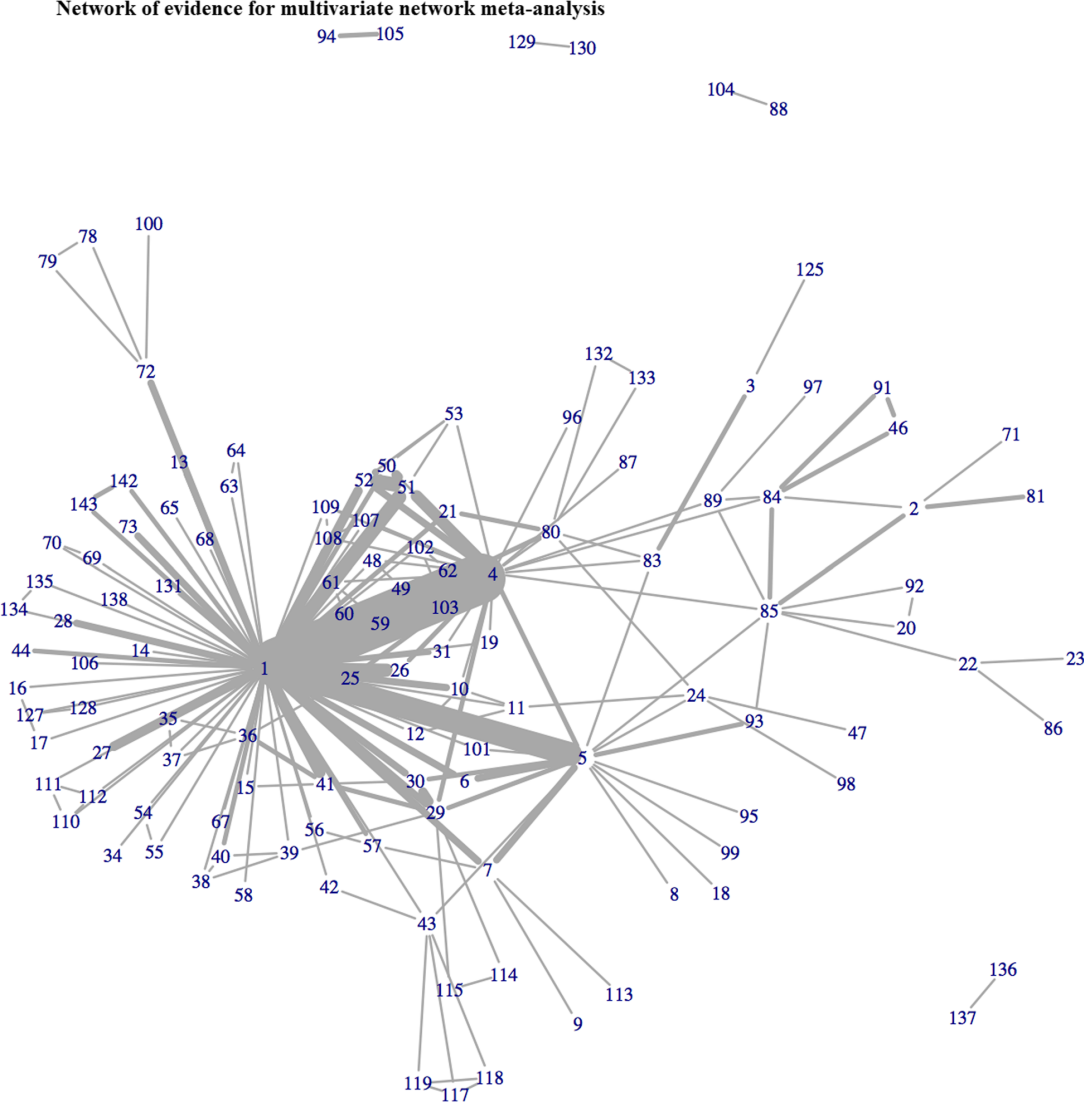
Network of evidence for multivariate network meta-analysis

A multivariate approach allowed for the inclusion of 143 studies evaluating 115 interventions for OAB across all 3 outcomes. Previously, univariate analyses of urinary incontinence, voiding frequency and urgency episodes included 115, 119, and 60 studies evaluating 97, 100, and 54 interventions, respectively. Results for univariate analyses are given in Additional file 5 for completeness. Figure 5 illustrates the network of evidence for MVNMA evaluating incontinence, voiding, and urgency episodes. Incorporating a multivariate approach increased the ability to include more interventions for all three outcomes, compared to that of univariate analyses displayed in Figs. 1, 2 and 3. This was particularly apparent for urgency episodes, with which all interventions evaluated for incontinence and voiding could now be evaluated for urgency. Furthermore, for interventions that were evaluated for urgency in the original trials but disconnected from the univariate network of evidence (Fig. 3), e.g. reflexology (71), a MVNMA could borrow information between outcomes for which they were connected, in order to complete the network of evidence. However, eight treatments remained disconnected from the multivariate network of evidence: darifenacin 7.5-15mg once daily + BT (88), tolterodine + BT (94), darifenacin ER 7.5-15mg once daily (104), tolterodine (105), lidocaine gel 2x6ml (129), emepronium bromide immediate release 200mg three times a day (130), sacral nerve stimulation + tolterodine extended release 2mg once daily (136), tolterodine extended release 2mg once daily (137). These interventions were disconnected in all networks of evidence for univariate NMAs evaluating each of the outcomes of interest; and thus, borrowing information between outcomes had little impact on the inclusion of these particular interventions in multivariate analyses.

Table 2 displays the estimated posterior median reduction and 95% credible intervals for change from baseline in incontinence, voiding, and urgency episodes. Treatment effect estimates were first ranked according to their effectiveness in reducing incontinence episodes, then by voiding frequency, and finally by urgency episodes. Sacral nerve stimulation appeared to be the most effective intervention for the management of urinary incontinence, voiding and urgency with an estimated posterior median reduction of -7.43 (95%CrI: -9.59,-4.73), -7.64 (95%CrI: -9.82,-4.91) and -7.96 (95%CrI: -10.19,-5.29) episodes, relative to placebo, respectively.

**Table 2:** Estimated posterior median difference (and 95% credible intervals) in change from baseline for urinary incontinence, voiding and urgency episodes obtained from multivariate network meta-analysis

**Treatment**	**Code**	**Incontinence episodes*†***	**Voiding episodes*†***	**Urgency episodes*†***
Sacral nerve stimulation	(81)	-7.43 (-9.59,-4.73)	-7.64 (-9.82,-4.91)	-7.96 (-10.19,-5.29)
OnaBoNT-A 200u trigone sparing	(73)	-2.38 (-3.13,-1.66)	-2.61 (-3.38,-1.9)	-2.9 (-3.76,-2.14)
Estradiol 3mg intravaginally	(128)	-1.91 (-3.6,-0.26)	-2.13 (-3.82,-0.51)	-2.44 (-4.18,-0.72)
Oxybutynin IR 2.5mg b.i.d + Salivary pastilles	(98)	-1.84 (-3.51,-0.17)	-2.06 (-3.73,-0.35)	-2.39 (-4.09,-0.7)
OnaBoNT-A 100u bladder base + trigone	(79)	-1.73 (-3.26,-0.12)	-1.95 (-3.48,-0.35)	-2.25 (-3.84,-0.62)
Electrostimulation + PFE + Bladder training	(97)	-1.6 (-2.52,-0.7)	-1.8 (-2.77,-0.91)	-2.13 (-3.05,-1.16)
Solifenacin/trospium + placebo injection	(100)	-1.64 (-2.56,-0.59)	-1.85 (-2.82,-0.8)	-2.14 (-3.17,-1.12)
OnaBoNTA 100u trigone sparing	(72)	-1.58 (-1.96,-1.21)	-1.69 (-2.04,-1.36)	-2.06 (-2.53,-1.64)
OnaBoNT-A 100u bladder body + trigone	(78)	-1.48 (-2.43,-0.48)	-1.7 (-2.67,-0.69)	-2.01 (-3.02,-1.05)
Oxybutynin intravesically 5mg t.i.d	(14)	-1.31 (-2.4,-0.07)	-1.53 (-2.63,-0.28)	-1.84 (-2.95,-0.54)
Tolerodine ER 4mg q.d + Neurostimulation	(96)	-1.35 (-1.76,-0.96)	-1.6 (-2.05,-1.15)	-1.9 (-2.43,-1.38)
Propiverine 30mg b.i.d	(42)	-1.3 (-3.48,0.7)	-1.53 (-3.69,0.47)	-1.87 (-4.06,0.26)
Estriol 1mg intravesival	(131)	-1.31 (-2.53,0.05)	-1.54 (-2.72,-0.19)	-1.85 (-3.11,-0.45)
Oxybutynin ER 10mg q.d	(8)	-1.06 (-1.53,-0.56)	-1.29 (-1.8,-0.78)	-1.59 (-2.18,-0.99)
Mirabegron 100mg b.i.d	(48)	-1.05 (-1.69,-0.43)	-1.26 (-1.9,-0.64)	-1.58 (-2.29,-0.83)
Solifenacin ER 10mg q.d	(30)	-0.87 (-1.09,-0.65)	-1.13 (-1.35,-0.92)	-1.41 (-1.78,-1.04)
Imidafenacin IR 0.25mg b.i.d	(37)	-0.88 (-1.49,-0.32)	-1.11 (-1.72,-0.53)	-1.42 (-2.06,-0.75)
Mirabegron 150mg b.i.d	(49)	-0.83 (-1.69,-0.08)	-1.07 (-1.92,-0.32)	-1.37 (-2.29,-0.58)
Pregabalin 150mg b.i.d + Tolterodine ER 4mg q.d	(102)	-0.83 (-1.57,-0.2)	-1.06 (-1.78,-0.45)	-1.36 (-2.15,-0.67)
Oxybutynin IR 3mg t.i.d	(19)	-0.83 (-1.17,-0.47)	-1.02 (-1.4,-0.64)	-1.35 (-1.81,-0.88)
Tolterodine ER 4mg q.d + Behaviour therapy	(87)	-0.78 (-1.47,-0.15)	-1 (-1.71,-0.35)	-1.32 (-2.03,-0.62)
Propiverine ER 30mg q.d	(42)	-0.76 (-1.27,-0.26)	-0.99 (-1.54,-0.43)	-1.3 (-1.92,-0.7)
Darifenacin ER 30mg q.d	(38)	-0.73 (-1.35,-0.13)	-0.95 (-1.59,-0.33)	-1.26 (-1.9,-0.57)
Tolterodine ER 2mg b.i.d + Oestrogen 0.625mg 2xwk	(99)	-0.72 (-1.22,-0.22)	-0.94 (-1.5,-0.38)	-1.26 (-1.88,-0.66)
Fesoterodine ER 8mg q.d	(26)	-0.71 (-0.9,-0.53)	-0.94 (-1.14,-0.76)	-1.26 (-1.62,-0.9)
Trospium IR 15mg t.i.d + Physiotherapy	(91)	-0.7 (-1.54,0.14)	-0.95 (-1.8,-0.1)	-1.24 (-2.11,-0.39)
Solifenacin ER (5mg-10mg) q.d	(31)	-0.67 (-0.91,-0.43)	-0.85 (-1.09,-0.61)	-1.18 (-1.56,-0.78)
Solabegron 125mg b.i.d	(55)	-0.64 (-0.92,-0.38)	-0.87 (-1.16,-0.61)	-1.18 (-1.58,-0.78)
Mirabegron 25mg q.d	(50)	-0.63 (-0.9,-0.36)	-0.85 (-1.12,-0.57)	-1.14 (-1.54,-0.74)
Oxybutynin vaginal ring 6mg q.d	(17)	-0.61 (-1.16,-0.03)	-0.86 (-1.41,-0.27)	-1.15 (-1.78,-0.53)
Solifenacin ER 5mg - 15mg q.d	(34)	-0.61 (-1.11,-0.12)	-0.82 (-1.38,-0.29)	-1.12 (-1.75,-0.54)
Trospium ER 60mg q.d	(44)	-0.6 (-0.94,-0.24)	-0.81 (-1.15,-0.45)	-1.12 (-1.58,-0.66)
Cizolirtine Citrate 400mg b.i.d	(57)	-0.58 (-1.21,-0.04)	-0.83 (-1.47,-0.24)	-1.14 (-1.81,-0.47)
Mirabegron 100mg q.d	(52)	-0.59 (-0.79,-0.4)	-0.78 (-0.97,-0.57)	-1.1 (-1.45,-0.75)
Solifenacin ER 5mg q.d	(29)	-0.57 (-0.79,-0.38)	-0.73 (-0.93,-0.56)	-1.09 (-1.46,-0.73)
Mirabegron 50mg q.d	(51)	-0.56 (-0.73,-0.4)	-0.82 (-0.99,-0.65)	-1.09 (-1.43,-0.74)
Oxybutynin IR (2.5-5mg) b.i.d	(24)	-0.53 (-1.14,0.12)	-0.74 (-1.34,-0.09)	-1.05 (-1.72,-0.35)
Oxybutynin IR 5mg t.i.d	(7)	-0.53 (-0.91,-0.14)	-0.73 (-1.12,-0.34)	-1.07 (-1.55,-0.53)
Mirabegron 200mg q.d	(53)	-0.52 (-1.11,-0.01)	-0.74 (-1.38,-0.19)	-1.06 (-1.72,-0.43)
Propiverine IR 15mg b.i.d	(43)	-0.53 (-0.94,-0.1)	-0.71 (-1.15,-0.25)	-1.04 (-1.56,-0.5)
Oxybutynin vaginal ring 4mg q.d	(16)	-0.51 (-1.12,0.12)	-0.73 (-1.32,-0.1)	-1.04 (-1.66,-0.39)
Pregabalin 150mg b.i.d	(62)	-0.49 (-1.02,0.06)	-0.74 (-1.24,-0.25)	-1.03 (-1.61,-0.45)
Tolterodine IR 2mg b.i.d + Pilocarpine 9mg b.i.d	(101)	-0.5 (-0.78,-0.21)	-0.73 (-1.06,-0.41)	-1.03 (-1.47,-0.6)
Fesoterodine ER 4mg q.d	(25)	-0.49 (-0.67,-0.33)	-0.7 (-0.87,-0.55)	-1.02 (-1.37,-0.68)
Oxybutynin chloride topical gel 1g q.d	(13)	-0.5 (-0.93,-0.06)	-0.71 (-1.15,-0.28)	-1.03 (-1.54,-0.5)
Tolterodine ER 4mg q.d	(4)	-0.49 (-0.6,-0.39)	-0.64 (-0.75,-0.52)	-0.98 (-1.28,-0.68)
Darifenacin ER 15mg q.d	(40)	-0.47 (-0.94,0.03)	-0.68 (-1.18,-0.11)	-1 (-1.55,-0.37)
Electromagnetic stimulation	(125)	-0.45 (-2.54,1.46)	-0.67 (-2.73,1.22)	-0.98 (-3.1,0.98)
Oxybutynin gel 84mg/day	(134)	-0.45 (-1.03,0.2)	-0.71 (-1.28,-0.07)	-1.01 (-1.64,-0.25)
Tolterodine IR 2mg b.i.d	(5)	-0.45 (-0.58,-0.31)	-0.69 (-0.84,-0.53)	-0.97 (-1.3,-0.64)
Tolterodine IR 2mg b.i.d + PFE	(95)	-0.44 (-1.06,0.22)	-0.65 (-1.29,0.06)	-0.96 (-1.62,-0.23)
Tolterodine IR 2mg b.i.d + BT	(93)	-0.41 (-0.96,0.11)	-0.67 (-1.23,-0.13)	-0.96 (-1.57,-0.37)
Propiverine ER 20mg q.d	(41)	-0.41 (-0.59,-0.22)	-0.65 (-0.84,-0.45)	-0.95 (-1.3,-0.59)
Trospium chloride 45mg t.i.d	(47)	-0.41 (-1.12,0.4)	-0.63 (-1.31,0.13)	-0.94 (-1.69,-0.08)
Elocalcitol 75mg	(70)	-0.39 (-0.91,0.21)	-0.56 (-1.08,0.07)	-0.9 (-1.53,-0.25)
Propiverine 45mg t.i.d	(118)	-0.38 (-2.55,1.76)	-0.61 (-2.75,1.58)	-0.93 (-3.13,1.32)
Fesoterodine ER (4mg-8mg) q.d	(27)	-0.36 (-0.57,-0.13)	-0.64 (-0.84,-0.44)	-0.91 (-1.27,-0.55)
Tolterodine IR 1mg b.i.d	(6)	-0.34 (-0.67,-0.01)	-0.57 (-0.92,-0.21)	-0.88 (-1.35,-0.42)
Imidafenacin IR 0.1mg b.i.d	(36)	-0.35 (-0.62,-0.1)	-0.53 (-0.8,-0.28)	-0.86 (-1.26,-0.44)
Terodiline IR 25mg b.i.d	(28)	-0.35 (-0.81,0.09)	-0.51 (-0.96,-0.09)	-0.86 (-1.37,-0.28)
Oxybutynin transdermal 3.9mg/day	(10)	-0.32 (-0.64,-0.03)	-0.55 (-0.87,-0.24)	-0.84 (-1.28,-0.44)
Oxybutynin gel 56mg/day	(135)	-0.32 (-0.95,0.33)	-0.48 (-1.1,0.15)	-0.81 (-1.53,-0.08)
Oxbutynin patch 73.5mg	(15)	-0.31 (-0.65,0.05)	-0.53 (-0.93,-0.11)	-0.84 (-1.31,-0.34)
Imidafenacin IR 0.05mg b.i.d	(35)	-0.29 (-0.73,0.17)	-0.53 (-0.99,-0.05)	-0.82 (-1.36,-0.26)
Darifenacin ER 7.5mg q.d	(39)	-0.28 (-0.85,0.26)	-0.5 (-1.09,0.06)	-0.83 (-1.46,-0.2)
Elocalcitol 150mg	(69)	-0.28 (-0.82,0.33)	-0.5 (-1.05,0.12)	-0.8 (-1.45,-0.13)
Duloxetine IR 40mg b.i.d	(65)	-0.27 (-0.77,0.24)	-0.5 (-1.06,0.06)	-0.8 (-1.43,-0.2)
Solabegron 50mg b.i.d	(54)	-0.24 (-0.52,0.03)	-0.47 (-0.75,-0.21)	-0.77 (-1.18,-0.38)
Lipo-BoNTA	(138)	-0.21 (-1.06,0.64)	-0.46 (-1.35,0.42)	-0.77 (-1.68,0.19)
Tarafenacin 0.4mg q.d	(82)	-0.2 (-0.87,0.51)	-0.45 (-1.1,0.27)	-0.74 (-1.45,0.07)
Serlopitant 0.25mg q.d	(107)	-0.21 (-0.73,0.37)	-0.42 (-0.91,0.09)	-0.72 (-1.36,-0.12)
Serlopitant 4mg q.d	(109)	-0.19 (-0.76,0.36)	-0.41 (-0.94,0.1)	-0.71 (-1.34,-0.11)
Pregabalin 75mg b.i.d + Tolterodine ER 2mg q.d	(103)	-0.18 (-0.66,0.3)	-0.42 (-0.9,0.04)	-0.71 (-1.29,-0.17)
Oxybutynin 20mg intravesically q.d	(106)	-0.15 (-1.63,1.3)	-0.36 (-1.83,1.05)	-0.65 (-2.14,0.75)
PFMT + BT	(89)	-0.1 (-0.66,0.43)	-0.37 (-0.97,0.2)	-0.66 (-1.28,-0.04)
Cizolirtine citrate 200mg b.i.d	(56)	-0.12 (-1.5,1.07)	-0.33 (-1.69,0.84)	-0.63 (-2.03,0.52)
Oxybutynin IR 2.5mg t.i.d	(21)	-0.1 (-0.41,0.21)	-0.42 (-0.9,-0.07)	-0.63 (-1.08,-0.17)
Percutaneous tibial nerve stimulation	(83)	-0.09 (-1.07,1.21)	-0.35 (-1.32,0.98)	-0.63 (-1.61,0.71)
Electrostimulation + vaginal oestrogen cream 1.25mg/day	(133)	-0.07 (-0.75,0.63)	-0.23 (-0.9,0.48)	-0.63 (-1.35,0.13)
Electrostimulation	(80)	-0.03 (-0.48,0.43)	-0.31 (-0.8,0.13)	-0.67 (-1.26,-0.14)
ONO-8539 100mg b.i.d	(60)	-0.02 (-0.66,0.61)	-0.22 (-0.87,0.41)	-0.53 (-1.23,0.16)
Oxybutynin ER 15mg q.d	(9)	-0.01 (-1.17,0.83)	-0.22 (-1.41,0.63)	-0.54 (-1.74,0.35)
Netupitant 200mg q.d	(112)	-0.02 (-1.19,1.09)	-0.23 (-1.42,0.82)	-0.52 (-1.75,0.53)
Estradiol 25mg	(68)	0.01 (-0.37,0.37)	-0.21 (-0.67,0.2)	-0.53 (-1.03,-0.02)
Placebo	(1)	NA	NA	NA
Netupitant 100mg q.d	(111)	0.01 (-1.1,0.96)	-0.2 (-1.29,0.71)	-0.52 (-1.62,0.4)
PFMT	(84)	0.07 (-0.63,0.64)	-0.18 (-0.85,0.43)	-0.48 (-1.12,0.18)
Oxybutynin transdermal 1.3mg/day	(11)	0.07 (-0.46,0.6)	-0.14 (-0.67,0.38)	-0.45 (-1.06,0.11)
ZD0947IL 25mg/day	(58)	0.07 (-0.81,0.98)	-0.12 (-1.03,0.82)	-0.45 (-1.39,0.52)
Serlopitant 1mg q.d	(108)	0.08 (-0.45,0.66)	-0.14 (-0.64,0.38)	-0.44 (-1.05,0.17)
Netupitant 50mg q.d	(110)	0.13 (-1.01,1.11)	-0.1 (-1.19,0.87)	-0.4 (-1.54,0.57)
Bladder Training	(85)	0.15 (-0.37,0.66)	-0.07 (-0.58,0.46)	-0.4 (-0.91,0.19)
Tarafenacin 0.2mg q.d	(90)	0.15 (-0.55,0.83)	-0.06 (-0.77,0.64)	-0.38 (-1.15,0.38)
Oxybutynin ER 2.5mg q.d + Bladder training	(92)	0.22 (-0.83,1.31)	0 (-1.05,1.11)	-0.32 (-1.34,0.84)
Oxybutynin transdermal 2.6mg/day	(12)	0.22 (-0.3,0.74)	-0.03 (-0.55,0.5)	-0.32 (-0.93,0.29)
Oxybutynin ER (5-30mg) q.d	(22)	0.23 (-0.34,0.85)	0.03 (-0.56,0.67)	-0.29 (-0.89,0.38)
Resiniferatoxin 50nM	(67)	0.27 (-0.9,1.61)	0.04 (-1.13,1.36)	-0.26 (-1.48,1.07)
Vaginal oestrogen cream 1.25mg/day	(132)	0.29 (-0.24,0.81)	0.03 (-0.5,0.55)	-0.22 (-0.81,0.41)
Flavoxate chloride 200mg q.d	(64)	0.34 (-0.36,1.05)	0.12 (-0.62,0.88)	-0.2 (-0.95,0.61)
Oxybutynin IR 5mg b.i.d	(18)	0.35 (-0.25,0.95)	0.13 (-0.5,0.75)	-0.17 (-0.88,0.49)
Emepronium bromide 200mg q.d	(63)	0.37 (-0.28,1.05)	0.15 (-0.55,0.87)	-0.16 (-0.9,0.6)
ONO-8539 300mg b.i.d	(61)	0.45 (-0.21,1.14)	0.22 (-0.44,0.91)	-0.1 (-0.78,0.65)
Propantheline Bromide 15mg t.i.d	(113)	0.45 (-0.67,2.07)	0.22 (-0.88,1.89)	-0.09 (-1.26,1.73)
Oxybutynin ER 2.5mg q.d	(20)	0.53 (-0.33,1.49)	0.29 (-0.61,1.25)	-0.01 (-0.9,0.94)
Estradiol 1mg intravaginally	(127)	0.58 (-0.74,2.02)	0.36 (-0.95,1.8)	0.04 (-1.33,1.52)
Propiverine 60mg q.d	(119)	0.59 (-1.97,2.89)	0.36 (-2.19,2.69)	0.05 (-2.45,2.45)
ONO-8539 30mg b.i.d	(59)	0.56 (-0.04,1.2)	0.34 (-0.27,0.96)	0.04 (-0.63,0.75)
Sham Therapy	(3)	0.54 (-0.55,2)	0.35 (-0.75,1.82)	0.03 (-1.02,1.54)
Trospium IR 15mg t.i.d	(46)	0.6 (-0.32,1.46)	0.43 (-0.49,1.36)	0.08 (-0.82,1.04)
Oxybutynin IR (5-20mg)	(23)	0.68 (-0.66,1.94)	0.46 (-0.91,1.73)	0.16 (-1.24,1.46)
Oxybutynin ER 5-30mg/day + Behaviour therapy	(22)	0.96 (-0.08,2.17)	0.72 (-0.3,1.94)	0.44 (-0.62,1.63)
Control	(2)	0.99 (0.22,2.01)	0.82 (0.04,1.87)	0.47 (-0.31,1.52)
Reflexology	(71)	1.03 (0.25,2.15)	0.78 (-0.05,1.88)	0.48 (-0.36,1.61)
Naftopidil 25mg q.d	(114)	3.46 (1.53,5.44)	3.24 (1.29,5.2)	2.94 (0.95,4.99)
Solifenacin succinate 5mg q.d + Naftopidil 25mg q.d	(115)	5.26 (2.92,7.79)	5.04 (2.71,7.54)	4.72 (2.43,7.26)

^*†*^median relative to a placebo intervention

Adopting a multivariate approach changed the overall clinical decision for urgency episodes. In univariate analyses, electrostimulation with vaginal oestrogen cream 1.25mg/day appeared to be the most effective intervention for reducing urgency (see Additional file 5). Accounting for the correlation between outcomes using MVNMA, sacral nerve stimulation appeared to be the most promising intervention overall.

### Multivariate network meta-analysis incorporating class effects

Table 3 displays the estimated posterior median reduction and 95% credible intervals for change from baseline in incontinence, voiding, and urgency episodes obtained from multivariate network meta-analysis incorporating class effects. Sacral nerve stimulation appeared to be the most effective intervention for reducing incontinence, voiding and urgency episodes with an estimated posterior median reduction of -8 (95%CrI: -9.54, -6.27), -8.19 (95%CrI: -9.69,-6.49) and -8.49(95%CrI: -10.11,-6.78) episodes, relative to placebo, respectively.

**Table 3:** Estimated posterior median difference (and 95% credible intervals) in change from baseline for urinary incontinence, voiding and urgency episodes obtained from multivariate network meta-analysis incorporating class effects. *Continued*

**Treatment**	**Code**	**Incontinence episodes*†***	**Voiding episodes*†***	**Urgency episodes*†***
Sacral nerve stimulation	(81)	-8 (-9.54,-6.27)	-8.19 (-9.69,-6.49)	-8.49 (-10.11,-6.78)
OnaBoNT-A 200u trigone sparing	(73)	-2.04 (-3.09,-1.29)	-2.26 (-3.31,-1.51)	-2.55 (-3.6,-1.74)
Oxybutynin IR 2.5mg b.i.d + Salivary pastilles	(98)	-2.07 (-3.42,0.03)	-2.3 (-3.62,-0.22)	-2.64 (-3.85,-0.64)
Electrostimulation + PFMT + BT	(97)	-1.78 (-2.58,-1.06)	-1.97 (-2.8,-1.25)	-2.3 (-3.21,-1.58)
Solifenacin/trospium + placebo injection	(100)	-1.8 (-2.45,-0.79)	-2.01 (-2.67,-0.94)	-2.33 (-3.08,-1.22)
OnaBoNT-A 100u bladder base + trigone	(79)	-1.78 (-3.31,-0.92)	-2.01 (-3.54,-1.14)	-2.35 (-4,-1.36)
OnaBoNT-A 100u bladder body + trigone	(78)	-1.68 (-2.6,-0.92)	-1.89 (-2.85,-1.11)	-2.23 (-3.24,-1.4)
OnaBoNTA 100u trigone sparing	(72)	-1.66 (-2.05,-1.3)	-1.73 (-2.07,-1.41)	-2.14 (-2.66,-1.7)
Tolterodine ER 4mg q.d + Neurostimulation	(96)	-1.32 (-1.7,-0.95)	-1.57 (-2.05,-1.14)	-1.88 (-2.44,-1.43)
Estriol 1mg intravesically	(131)	-1.52 (-2.88,-0.31)	-1.76 (-3.07,-0.58)	-2.06 (-3.4,-0.91)
Trospium IR 15mg t.i.d + Physiotherapy	(91)	-1.16 (-1.95,-0.43)	-1.4 (-2.22,-0.62)	-1.74 (-2.58,-0.93)
Estradiol 3mg intravaginally	(128)	-0.87 (-2.13,1.08)	-1.08 (-2.31,0.82)	-1.41 (-2.64,0.53)
Solifenacin ER 10mg q.d	(30)	-0.82 (-1.06,-0.61)	-1.09 (-1.3,-0.9)	-1.37 (-1.74,-1.03)
Tolterodine ER 2mg b.i.d + Oestrogen 0.625mg 2xwk	(99)	-0.68 (-1.23,-0.22)	-0.89 (-1.48,-0.39)	-1.22 (-1.88,-0.66)
Tolterodine ER 4mg q.d + BT	(87)	-0.68 (-1.31,-0.07)	-0.88 (-1.61,-0.25)	-1.23 (-1.96,-0.57)
Pregabalin 150mg b.i.d + Tolterodine ER 4mg q.d	(102)	-0.85 (-1.42,-0.2)	-1.09 (-1.65,-0.46)	-1.41 (-2.05,-0.69)
Fesoterodine ER 8mg q.d	(26)	-0.69 (-0.87,-0.49)	-0.92 (-1.1,-0.72)	-1.25 (-1.6,-0.9)
Imidafenacin IR 0.25mg b.i.d	(37)	-0.72 (-1.32,-0.17)	-0.96 (-1.55,-0.34)	-1.28 (-1.98,-0.62)
Solifenacin ER (5mg-10mg) q.d	(31)	-0.68 (-0.9,-0.42)	-0.84 (-1.06,-0.61)	-1.2 (-1.54,-0.87)
Solifenacin ER 5mg - 15mg q.d	(34)	-0.64 (-1,-0.27)	-0.87 (-1.29,-0.45)	-1.2 (-1.66,-0.75)
Mirabegron 100mg b.i.d	(48)	-0.68 (-1.06,-0.4)	-0.87 (-1.27,-0.6)	-1.2 (-1.68,-0.88)
Solabegron 125mg b.i.d	(55)	-0.62 (-0.9,-0.34)	-0.84 (-1.12,-0.59)	-1.16 (-1.58,-0.79)
Propiverine ER 30mg b.i.d	(42)	-0.61 (-1.81,-0.1)	-0.85 (-2.06,-0.37)	-1.18 (-2.42,-0.6)
Darifenacin ER 30mg q.d	(38)	-0.59 (-1.15,-0.05)	-0.8 (-1.41,-0.2)	-1.15 (-1.73,-0.54)
Mirabegron ER 25mg q.d	(50)	-0.63 (-0.87,-0.42)	-0.85 (-1.05,-0.64)	-1.16 (-1.48,-0.84)
Mirabegron IR 150mg b.i.d	(49)	-0.61 (-0.91,-0.29)	-0.87 (-1.14,-0.55)	-1.18 (-1.53,-0.81)
Mirabegron ER 100mg q.d	(52)	-0.62 (-0.81,-0.44)	-0.8 (-0.96,-0.61)	-1.15 (-1.47,-0.87)
Mirabegron ER 200mg q.d	(53)	-0.62 (-0.91,-0.27)	-0.83 (-1.13,-0.47)	-1.17 (-1.53,-0.79)
Oxybutynin ER 10mg q.d	(8)	-0.58 (-1.09,-0.25)	-0.81 (-1.27,-0.47)	-1.14 (-1.57,-0.7)
Propiverine ER 30mg q.d	(42)	-0.56 (-1.05,-0.17)	-0.76 (-1.33,-0.37)	-1.08 (-1.72,-0.66)
Oxybutynin IR 3mg t.i.d	(19)	-0.6 (-0.94,-0.3)	-0.75 (-1.12,-0.45)	-1.09 (-1.56,-0.7)
Mirabegron ER 50mg q.d	(51)	-0.59 (-0.78,-0.42)	-0.84 (-0.99,-0.67)	-1.15 (-1.45,-0.84)
Solifenacin ER 5mg q.d	(29)	-0.6 (-0.8,-0.38)	-0.74 (-0.92,-0.58)	-1.14 (-1.48,-0.83)
Tolterodine IR 2mg b.i.d + BT	(93)	-0.57 (-1,0)	-0.86 (-1.28,-0.2)	-1.15 (-1.7,-0.47)
Cizolirtine Citrate 400mg b.i.d	(57)	-0.56 (-1.1,-0.05)	-0.8 (-1.37,-0.25)	-1.16 (-1.78,-0.56)
Trospium ER 60mg q.d	(44)	-0.55 (-0.92,-0.21)	-0.75 (-1.13,-0.41)	-1.08 (-1.59,-0.61)
Propiverine IR 45mg t.i.d	(118)	-0.52 (-1.14,0.1)	-0.73 (-1.35,-0.15)	-1.06 (-1.73,-0.39)
Tolterodine IR 2mg b.i.d + Pilocarpine 9mg b.i.d	(101)	-0.5 (-0.81,-0.23)	-0.74 (-1.11,-0.43)	-1.07 (-1.53,-0.68)
Fesoterodine ER 4mg q.d	(25)	-0.49 (-0.66,-0.32)	-0.7 (-0.86,-0.53)	-1.03 (-1.38,-0.72)
Pregabalin 150mg b.i.d	(62)	-0.5 (-1.08,0.12)	-0.75 (-1.3,-0.18)	-1.06 (-1.74,-0.35)
Darifenacin ER 15mg q.d	(40)	-0.46 (-0.83,0)	-0.65 (-1.05,-0.17)	-0.99 (-1.48,-0.47)
Propiverine IR 15mg b.i.d	(43)	-0.46 (-0.88,-0.1)	-0.62 (-1.07,-0.27)	-0.98 (-1.56,-0.58)
Tolterodine ER 4mg q.d	(4)	-0.5 (-0.6,-0.4)	-0.62 (-0.74,-0.52)	-0.99 (-1.29,-0.75)
Oxybutynin IR 5mg t.i.d	(7)	-0.45 (-0.78,-0.18)	-0.64 (-0.93,-0.36)	-0.98 (-1.37,-0.61)
Propiverine ER 20mg q.d	(41)	-0.42 (-0.6,-0.22)	-0.65 (-0.84,-0.48)	-0.98 (-1.35,-0.67)
Tolterodine IR 2mg b.i.d	(5)	-0.44 (-0.57,-0.3)	-0.67 (-0.82,-0.53)	-0.98 (-1.31,-0.7)
Propiverine ER 60mg q.d	(119)	-0.41 (-0.89,0.72)	-0.6 (-1.09,0.44)	-0.92 (-1.55,0.23)
Oxybutynin intravesically 5mg t.i.d	(14)	-0.41 (-0.97,-0.02)	-0.61 (-1.17,-0.23)	-0.96 (-1.52,-0.52)
Oxybutynin IR 2.5-5mg b.i.d	(24)	-0.46 (-0.8,-0.16)	-0.63 (-0.95,-0.38)	-1 (-1.36,-0.6)
Oxybutynin chloride topical gel 1g q.d	(13)	-0.43 (-0.78,-0.13)	-0.64 (-0.97,-0.35)	-0.99 (-1.35,-0.59)
Oxybutynin vaginal ring 6mg q.d	(17)	-0.39 (-0.78,0.01)	-0.63 (-1.01,-0.23)	-0.95 (-1.4,-0.44)
Tolterodine IR 2mg b.i.d + PFMT	(95)	-0.44 (-0.98,0.42)	-0.65 (-1.23,0.2)	-0.98 (-1.62,-0.2)
PFMT + BT	(89)	-0.38 (-0.83,0.03)	-0.66 (-1.16,-0.18)	-0.95 (-1.55,-0.44)
Tolterodine IR 1mg b.i.d	(6)	-0.39 (-0.64,-0.08)	-0.62 (-0.91,-0.31)	-0.94 (-1.35,-0.58)
Fesoterodine ER 4mg-8mg q.d	(27)	-0.37 (-0.59,-0.17)	-0.67 (-0.86,-0.46)	-0.96 (-1.33,-0.62)
Oxybutynin gel 84mg/day	(134)	-0.37 (-0.76,0)	-0.63 (-0.99,-0.28)	-0.96 (-1.33,-0.51)
Oxybutynin transdermal 3.9mg/day	(10)	-0.38 (-0.63,-0.14)	-0.61 (-0.86,-0.35)	-0.94 (-1.29,-0.58)
Oxybutynin vaginal ring 4mg q.d	(16)	-0.37 (-0.67,-0.03)	-0.56 (-0.87,-0.22)	-0.92 (-1.31,-0.48)
Imidafenacin 0.1mg b.i.d	(36)	-0.38 (-0.69,-0.1)	-0.55 (-0.81,-0.29)	-0.9 (-1.3,-0.5)
Terodiline 25mg b.i.d	(28)	-0.37 (-0.78,0.14)	-0.51 (-0.9,-0.05)	-0.88 (-1.44,-0.3)
Darifenacin ER 7.5mg q.d	(39)	-0.35 (-0.81,0.19)	-0.58 (-1.08,0.02)	-0.92 (-1.46,-0.29)
Oxybutynin gel 56mg/day	(135)	-0.35 (-0.69,0.06)	-0.52 (-0.82,-0.1)	-0.88 (-1.3,-0.38)
Oxbutynin patch 73.5mg	(15)	-0.34 (-0.57,-0.02)	-0.56 (-0.86,-0.15)	-0.89 (-1.27,-0.45)
Elocalcitol 75mg	(70)	-0.37 (-0.89,0.06)	-0.5 (-1.06,-0.07)	-0.89 (-1.57,-0.38)
Oxybutynin 20mg intravesically q.d	(106)	-0.33 (-0.75,0.42)	-0.55 (-0.95,0.27)	-0.9 (-1.34,-0.05)
Imidafenacin 0.05mg b.i.d	(35)	-0.32 (-0.76,0.23)	-0.57 (-1.03,0.03)	-0.9 (-1.42,-0.22)
Oxybutynin ER 15mg q.d	(9)	-0.31 (-0.78,0.12)	-0.53 (-0.98,-0.07)	-0.88 (-1.32,-0.31)
Oxybutynin IR 5-20mg	(23)	-0.29 (-0.67,0.26)	-0.52 (-0.9,0.07)	-0.85 (-1.32,-0.13)
Oxybutynin ER 5-30mg q.d	(22)	-0.3 (-0.63,0.02)	-0.5 (-0.88,-0.12)	-0.87 (-1.27,-0.39)
Trospium chloride IR 45mg t.i.d	(47)	-0.33 (-0.81,0.18)	-0.55 (-0.95,-0.12)	-0.89 (-1.38,-0.35)
Cizolirtine citrate 200mg b.i.d	(56)	-0.22 (-1.26,0.73)	-0.46 (-1.48,0.54)	-0.77 (-1.87,0.26)
Pelvic floor muscle training (PFMT)/Physiotherapy	(84)	-0.33 (-0.82,0.15)	-0.59 (-1.17,-0.01)	-0.89 (-1.54,-0.35)
Oxybutynin transdermal 1.3mg/day	(11)	-0.27 (-0.56,0.14)	-0.48 (-0.78,-0.04)	-0.82 (-1.26,-0.29)
Elocalcitol 150mg	(69)	-0.34 (-0.78,0.19)	-0.55 (-1.05,-0.04)	-0.86 (-1.48,-0.27)
Oxybutynin ER 2.5mg q.d	(20)	-0.27 (-0.68,0.36)	-0.54 (-0.95,0.15)	-0.86 (-1.33,-0.04)
Duloxetine 40mg b.i.d	(65)	-0.3 (-0.86,0.26)	-0.52 (-1.16,0.09)	-0.86 (-1.56,-0.16)
Bladder Training (BT)/ Behaviour Therapy	(85)	-0.26 (-0.56,0.05)	-0.45 (-0.8,-0.13)	-0.8 (-1.24,-0.4)
Solabegron IR 50mg b.i.d	(54)	-0.24 (-0.52,0.05)	-0.46 (-0.74,-0.18)	-0.79 (-1.22,-0.4)
Oxybutynin IR 2.5mg t.i.d	(21)	-0.19 (-0.45,0.1)	-0.58 (-0.99,-0.17)	-0.77 (-1.17,-0.34)
Oxybutynin transdermal 2.6mg/day	(12)	-0.18 (-0.52,0.35)	-0.43 (-0.75,0.16)	-0.77 (-1.2,-0.11)
Pregabalin 75mg b.i.d + Tolterodine ER 2mg q.d	(103)	-0.24 (-0.67,0.24)	-0.48 (-0.9,-0.04)	-0.78 (-1.33,-0.25)
Oxybutynin ER 2.5mg q.d + BT	(92)	-0.35 (-1.14,0.48)	-0.57 (-1.35,0.27)	-0.88 (-1.75,0.03)
Oxybutynin IR 5mg b.i.d	(18)	-0.15 (-0.49,0.35)	-0.39 (-0.78,0.15)	-0.75 (-1.19,-0.09)
Lipo-BoNTA 200U	(138)	-0.04 (-0.92,0.78)	-0.26 (-1.21,0.61)	-0.57 (-1.53,0.29)
Serlopitant 0.25mg q.d	(107)	-0.08 (-0.56,0.45)	-0.31 (-0.79,0.23)	-0.64 (-1.2,-0.01)
Serlopitant 4mg q.d	(109)	-0.07 (-0.61,0.44)	-0.3 (-0.81,0.24)	-0.63 (-1.21,0.01)
Tarafenacin 0.4mg q.d	(82)	-0.19 (-0.82,0.53)	-0.43 (-1.04,0.29)	-0.75 (-1.4,-0.02)
Electrostimulation + vaginal oestrogen cream 1.25mg/day	(133)	-0.08 (-0.77,0.61)	-0.22 (-0.91,0.5)	-0.68 (-1.51,0.16)
Electrostimulation	(80)	-0.05 (-0.48,0.37)	-0.35 (-0.89,0.15)	-0.76 (-1.44,-0.16)
Serlopitant 1mg q.d	(108)	0.06 (-0.48,0.61)	-0.15 (-0.63,0.39)	-0.5 (-1.08,0.15)
Estradiol 25mg	(68)	0.01 (-0.4,0.51)	-0.19 (-0.68,0.32)	-0.53 (-1.09,0.04)
Placebo	(1)	NA	NA	NA
Netupitant 200mg q.d	(112)	-0.09 (-0.98,0.74)	-0.3 (-1.23,0.51)	-0.63 (-1.54,0.2)
Netupitant 100mg q.d	(111)	-0.04 (-0.84,1.06)	-0.25 (-1.08,0.84)	-0.59 (-1.48,0.47)
Tarafenacin 0.2mg q.d	(90)	0.01 (-0.66,0.7)	-0.18 (-0.89,0.51)	-0.52 (-1.25,0.14)
Trospium IR 15mg t.i.d	(46)	-0.03 (-0.74,0.71)	-0.18 (-1.02,0.58)	-0.54 (-1.39,0.27)
ZD0947IL 25mg/day	(58)	0.07 (-0.61,0.87)	-0.1 (-0.84,0.67)	-0.42 (-1.28,0.24)
Netupitant 50mg q.d	(110)	0.06 (-0.61,0.81)	-0.14 (-0.83,0.51)	-0.48 (-1.33,0.22)
Electromagnetic stimulation	(125)	-1.25 (-4.03,0.42)	-1.47 (-4.25,0.17)	-1.85 (-4.57,-0.06)
Oxybutynin ER 5-30mg/day + Behaviour therapy	(22)	0.19 (-0.63,1.05)	-0.04 (-0.92,0.84)	-0.4 (-1.24,0.6)
ONO-8539 100mg b.i.d	(60)	0.15 (-0.43,0.77)	-0.04 (-0.62,0.55)	-0.37 (-1,0.3)
Percutaneous tibial nerve stimulation	(83)	-0.42 (-1.06,0.4)	-0.65 (-1.33,0.13)	-0.95 (-1.74,-0.22)
Vaginal oestrogen cream 1.25mg/day	(132)	0.28 (-0.22,0.75)	0 (-0.57,0.52)	-0.26 (-0.91,0.34)
Flavoxate chloride 200mg q.d	(64)	0.28 (-0.35,1.19)	0.05 (-0.59,0.97)	-0.29 (-0.93,0.64)
Resiniferatoxin 50nM	(67)	0.08 (-1.43,1.12)	-0.15 (-1.68,0.88)	-0.47 (-2.07,0.63)
Emepronium bromide ER 200mg q.d	(63)	0.36 (-0.65,1.17)	0.15 (-0.86,0.98)	-0.21 (-1.17,0.65)
ONO-8539 300mg b.i.d	(61)	0.43 (-0.11,1.07)	0.2 (-0.31,0.85)	-0.17 (-0.67,0.61)
Propantheline Bromide 15mg t.i.d	(113)	0.69 (-0.88,1.59)	0.46 (-0.99,1.35)	0.11 (-1.41,1.12)
Estradiol 1mg intravaginally	(127)	1.02 (-0.45,2.47)	0.82 (-0.61,2.26)	0.46 (-0.95,1.94)
ONO-8539 30mg b.i.d	(59)	0.58 (-0.1,1.2)	0.35 (-0.32,0.98)	0.02 (-0.67,0.7)
Control	(2)	0.49 (0,1.09)	0.33 (-0.1,0.94)	-0.02 (-0.62,0.6)
Reflexology	(71)	0.46 (-0.12,1.22)	0.22 (-0.45,0.97)	-0.06 (-0.79,0.7)
Sham Therapy	(3)	0.2 (-0.45,0.96)	0 (-0.65,0.73)	-0.34 (-1.08,0.34)
Naftopidil 25mg q.d	(114)	4.06 (2.41,5.02)	3.8 (2.21,4.79)	3.48 (1.81,4.49)
Solifenacin ER 5mg q.d + Naftopidil 25mg q.d	(115)	5.19 (3.67,7.37)	4.95 (3.47,7.15)	4.6 (3.22,6.91)

^*†*^median relative to a placebo intervention

Incorporating class effects in MVNMAs broadly increased the precision in treatment effect estimates (Table 3) compared to MVNMAs without incorporating class effects (Table 2). For example, for sacral nerve stimulation incorporating a hierarchical structure further increased precision in the treatment effect estimates by approximately 33%, 35%, and 28% for urinary incontinence, voiding and urgency episodes, respectively.

**Fig. 6:**
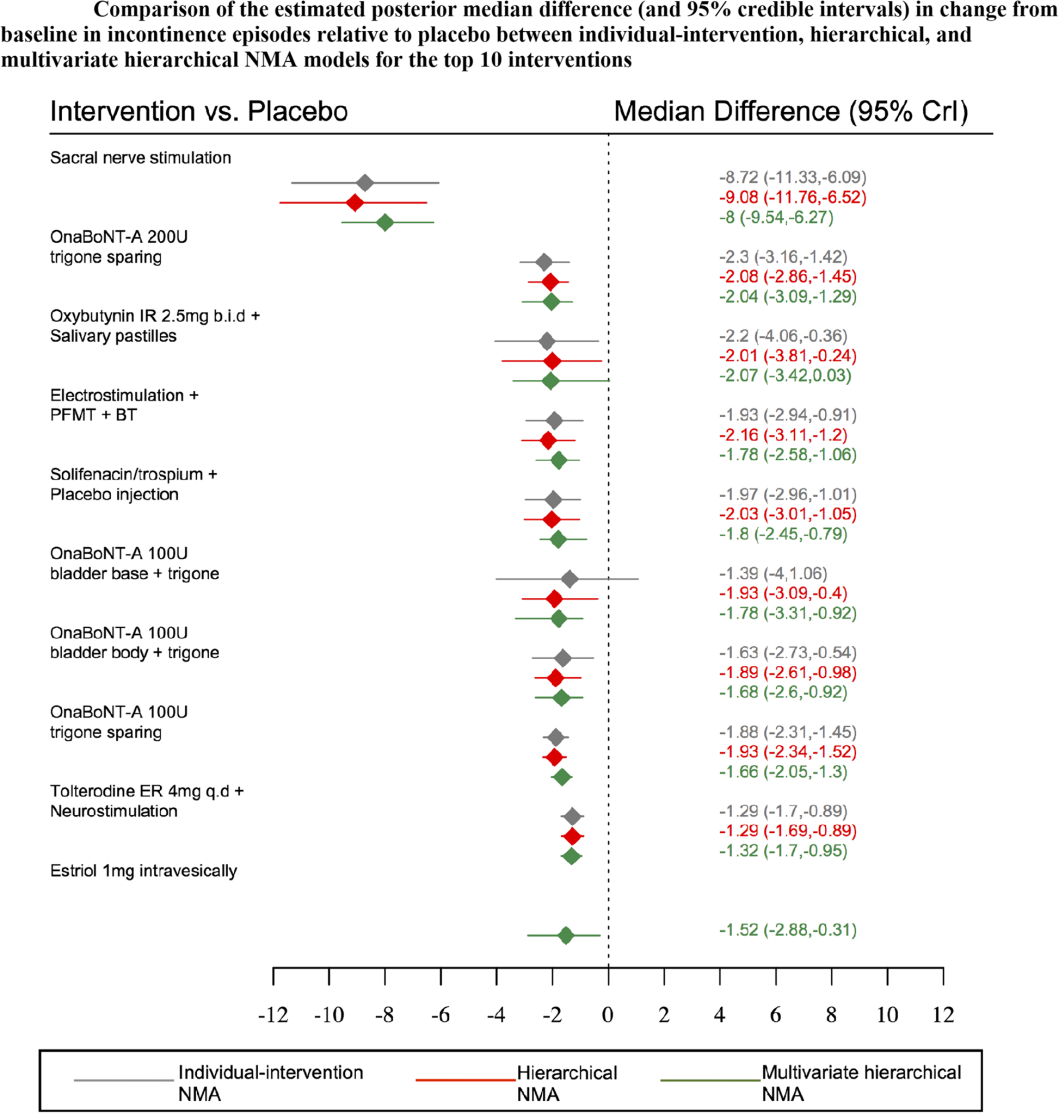
Comparison of the estimated posterior median difference (and 95% credible intervals) in change from baseline in incontinence episodes relative to placebo between individual-intervention, hierarchical, and multivariate hierarchical NMA models for the top 10 interventions

**Fig. 7:**
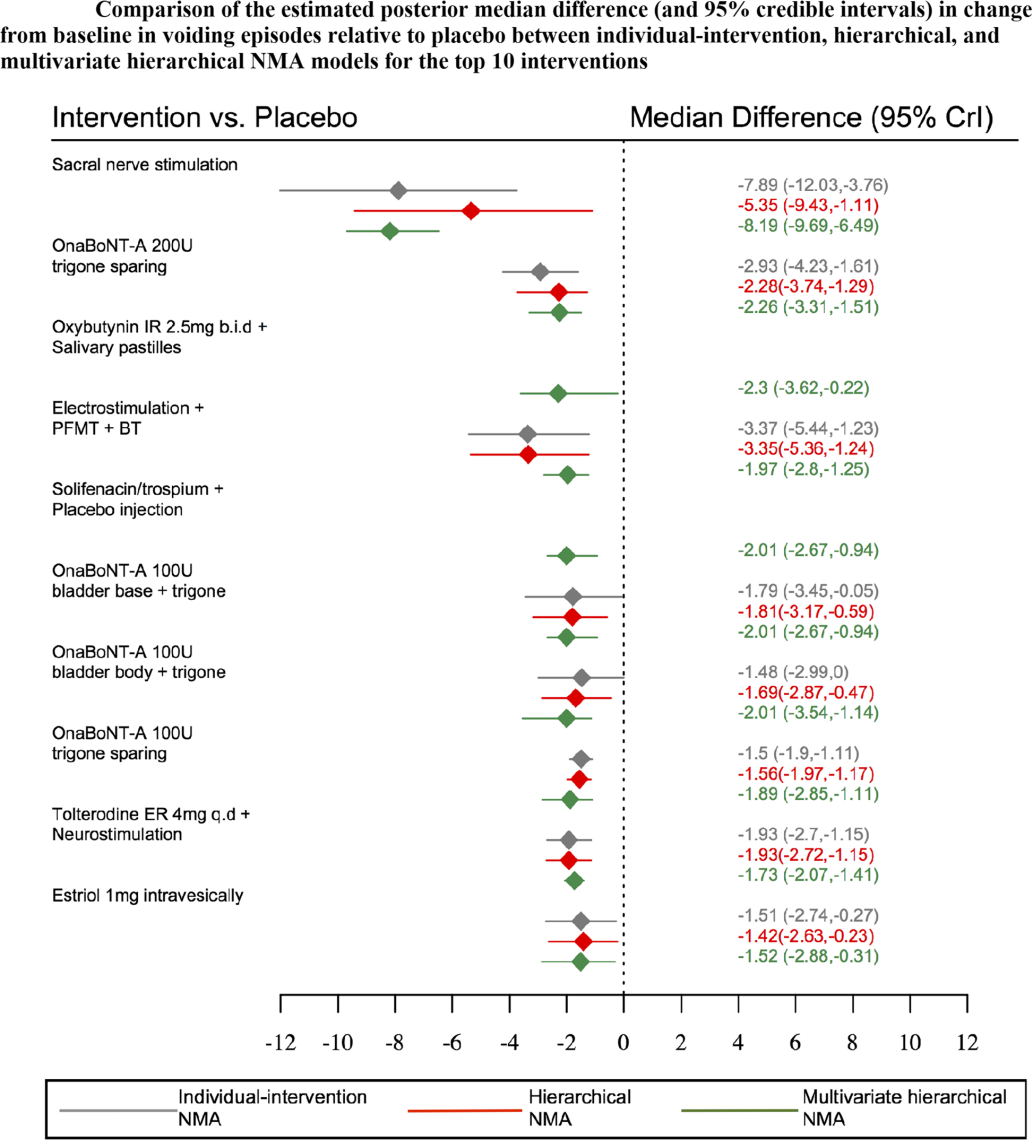
Comparison of the estimated posterior median difference (and 95% credible intervals) in change from baseline in voiding episodes relative to placebo between individual-intervention, hierarchical, and multivariate hierarchical NMA models for the top 10 interventions

**Fig. 8:**
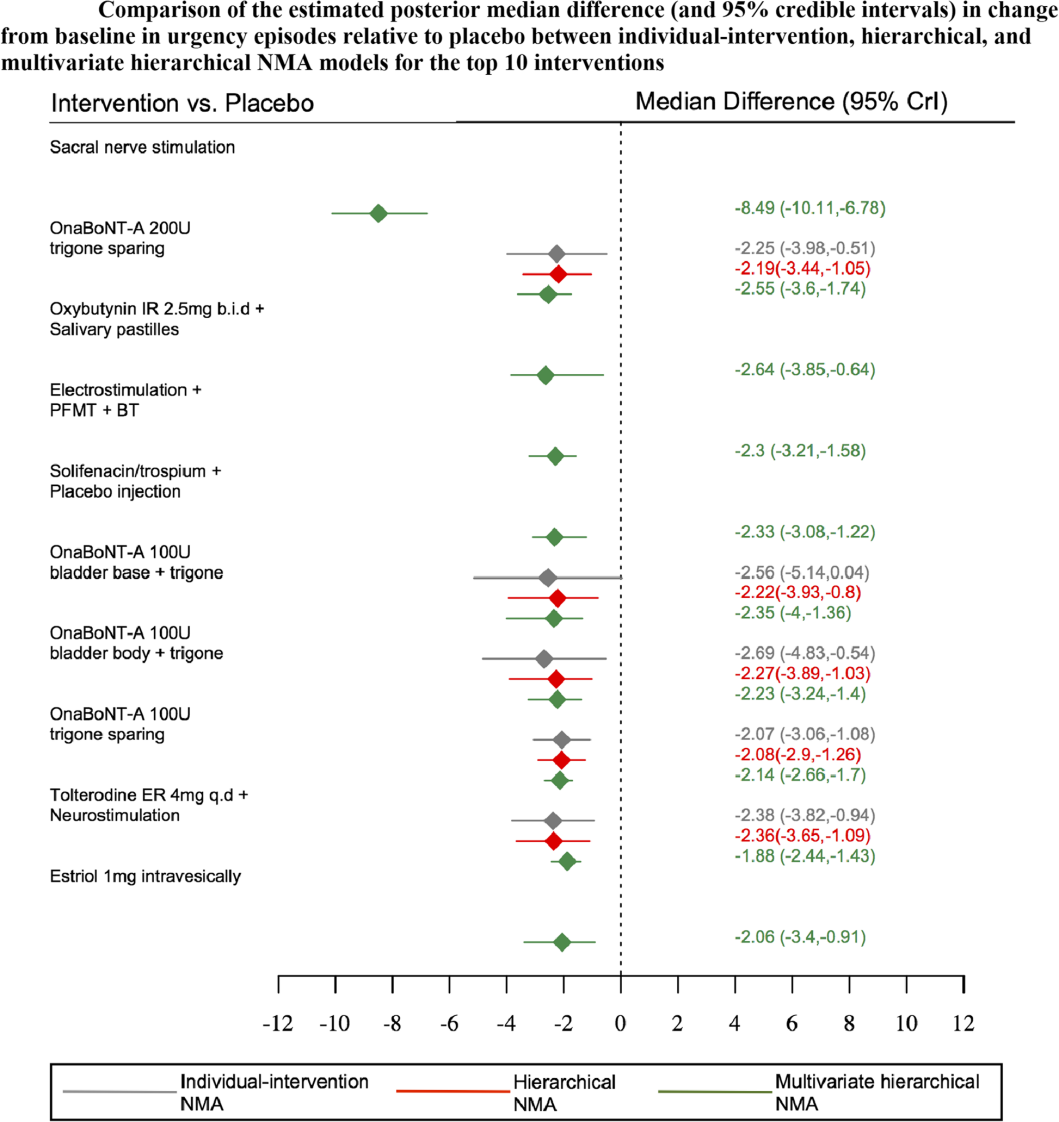
Comparison of the estimated posterior median difference (and 95% credible intervals) in change from baseline in urgency episodes relative to placebo between individual-intervention, hierarchical, and multivariate hierarchical NMA models for the top 10 interventions

Borrowing information between outcomes generally increased the precision in treatment effect estimates compared to univariate analyses (Figs. 6, 7, and 8). This finding was particularly apparent for sacral nerve stimulation, with which a multivariate hierarchical approach incorporating class effects increased precision in the estimated treatment effects by approximately 60% and 160% for urinary incontinence (Fig. 6) and voiding frequency (Fig. 7) respectively, compared to treatment effect estimates obtained from univariate NMAs (see additional file 5). For all interventions included in each of the univariate analyses, point estimates obtained from multivariate analyses were comparable with point estimates obtained from univariate analyses for all three outcomes (Figs. 6, 7, and 8).

### Treatment profiles

**Fig. 9:**
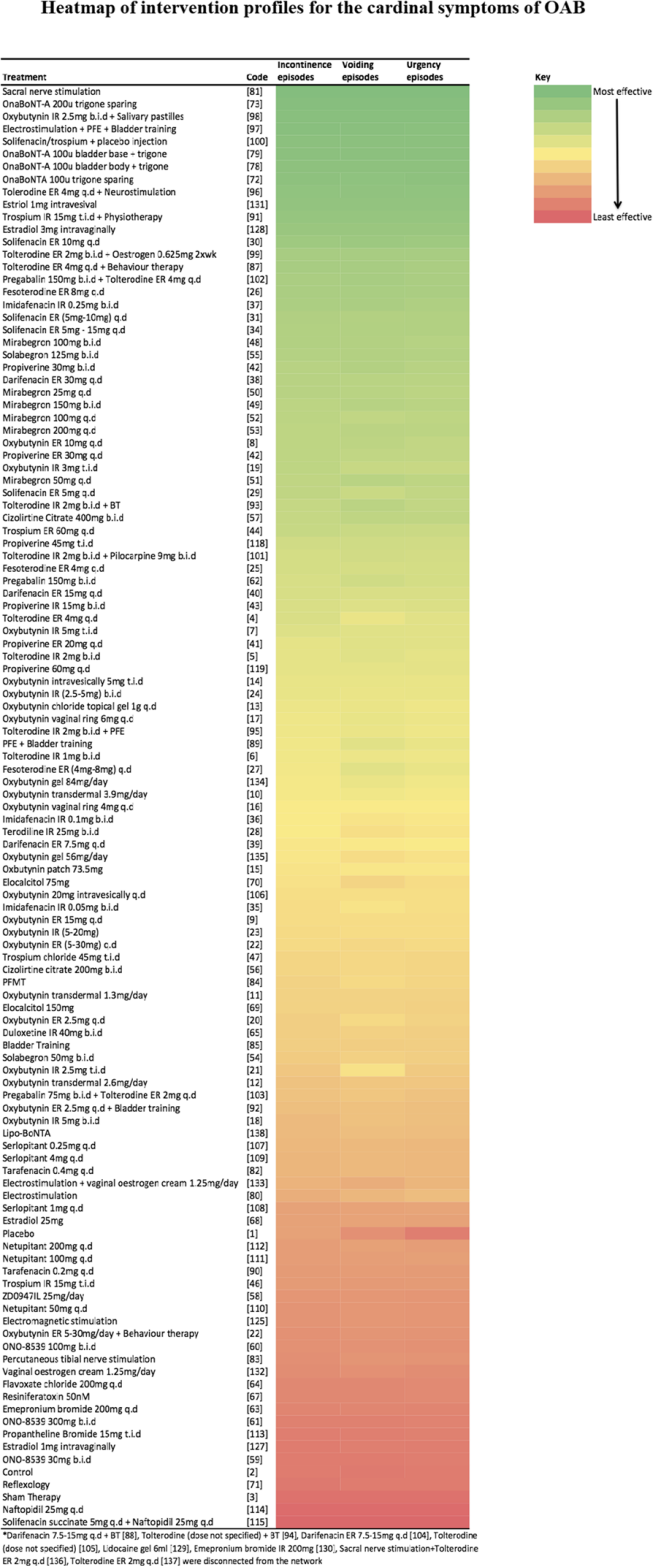
Heatmap of intervention profiles for the cardinal symptoms of OAB

Figure 9 illustrates the treatment profiles for all of the cardinal symptoms of OAB. Use of MVNMA allowed for the comparison of all interventions across all outcome measures, and thus completed the intervention profiles for efficacy outcomes. Generally, the treatment rankings were broadly similar to those obtained from univariate analyses (see Additional file 6). Sacral nerve stimulation appeared to be the most effective intervention across all three outcomes. Using multivariate analyses, estriol 1mg intravesically appeared to be amongst the top ten interventions (Fig. 9). Previously, estriol 1mg intravesically was only evaluated for voiding, and ranked amongst the top interventions (see Additional file 5). Borrowing information between outcomes allowed for estimation of treatment effects for both urinary incontinence and urgency episodes, and consequently estriol 1mg intravesically was ranked in tenth place across all outcome measures. However, the remaining top ten interventions remained unchanged.

### Convergence diagnostics

Convergence diagnostic plots for a small selection of basic parameters, *d*_1*j*_, the pooled effect estimate of intervention *j* relative to placebo, are given in Additional file 7. Both the between and within chain variability appeared to reach stability, and the ratio appeared to converge to 1. All plots appeared to show reducing autocorrelation with increased lag. History and trace plots took the appearance of random noise, with no obvious difference between multiple MCMC chains with very different starting values. Overall all diagnostic plots appeared to suggest that there was no evidence of non-convergence and both *d*_1*j*_ and *ζ* were estimated from samples which appeared to have a reasonable degree of mixing of the chains.

### Sensitivity analysis

Sensitivity analyses assessing different choices of prior distributions for the between-study standard deviations, the elements of the matrix ***V***^***1/2***^, and the variability of treatment effect profiles across outcomes, *ζ*, are given in Additional file 8. Different choices of prior distributions for variance parameters had very little impact in the treatment effect estimates for all three outcomes, and therefore the overall clinical decisions regarding interventions effectiveness remained the same. Thus suggesting that treatment effect estimates were robust to choice of prior distribution.

## Discussion

This paper extends the MVNMA framework described by Achana et al. [7], to incorporate the exchangeability of interventions belonging to the same class of interventions. This approach makes use of the correlations between multiple outcomes in order to predict and impute treatment effect estimates for missing data, and therefore, has the potential to limit the impact of outcome reporting bias, as well as, borrows strength between interventions belonging to the same class of treatment, which has the potential to increase precision in the treatment effect estimates.

In the OAB example, the datasets used in univariate analyses for each outcome independently, included 115, 119 and 60 studies evaluating 97, 100, and 54 interventions for incontinence, voiding, and urgency episodes, respectively. Despite urgency being documented as “the cardinal symptom" of OAB [25], it was sufficiently under-reported in the original trials, and consequently fewer interventions were able to be evaluated in univariate analyses. Adopting a multivariate approach, we were able to borrow information across outcomes and consequently included 143 studies evaluating all 115 interventions for the management of incontinence, voiding and urgency. Using this methodology completed the treatment profiles for all prominent symptoms of OAB, which in turn allows decision makers to make inferences regarding the potential treatment benefit for all interventions, across all salient outcomes. In doing so, sacral nerve stimulation appeared to be the most effective intervention for reducing incontinence, voiding and urgency episodes with an estimated posterior median reduction of -8 (95%CrI: -9.54, -6.27), -8.19 (95%CrI: -9.69,-6.49) and -8.49(95%CrI: -10.11,-6.78) episodes, relative to placebo, respectively. However, due to the limited number of studies and participants in which sacral nerve stimulation was evaluated, these results should be interpreted with caution.

Sacral nerve stimulation was not evaluated for urgency in the original trials and thus could not be assessed in univariate analyses. Using a multivariate approach therefore changed the overall clinical decision for the management of urgency, where electrostimulation in combination with vaginal oestrogen cream 1.25mg/day was found to be the most effective intervention from univariate analyses. Similarly, from univariate analyses, estriol 1mg intravesically appeared to be a promising intervention for voiding, but there was no data for all other outcomes. Using a multivariate approach, estriol 1mg intravesically ranked in the top 10 interventions for all three cardinal symptoms of OAB.

A key assumption of MVNMA is that the data is multivariately normally distributed. In situations with which we wish to synthesise multiple binomial outcomes, or a mixture of binomial and continuous outcomes, it is common to assume a normal approximation of binomial data on the log odds ratio (or log odds) scale [9]. This assumption has been shown to hold well when the proportions of events are close to 0.5, but poorly when the proportions of events are close to 0 or 1 [31]. In situations such as these more sophisticated methods, such as copula models, are required to appropriately capture the within-study variability using binomial data directly [32].

In this paper, we describe a missing data framework to incorporate estimates of uncertainty for trials that did not report any measure of variability in the mean treatment effects. It could be argued that trials reporting incomplete outcome data are at a higher risk of bias [33], and the impact of including such trials in meta-analyses should be thoroughly explored in sensitivity analyses.

A further assumption of MVNMA is that all data are assumed to be missing at random [17], which in the case of the OAB example may not be plausible [34]. It is likely that there is an element of selective reporting in the original trials, such that outcomes with which interventions perform particularly well are more likely to be reported [35]. In this situation, treatment effects may be exaggerated [17], though recent simulation studies suggest that a multivariate meta-analysis can lead to a more appropriate estimate of treatment effect in the presence of outcome reporting bias [5, 6] under a variety of missing data scenarios, including missing at random and missing not at random [6]. In order to obtain more accurate estimates of treatment effects for decision making, data are needed for all interventions, across all outcome measures. Following the Core Outcome Measures in Effectiveness Trials (COMET) initiative [36], there is a clear need to define a core outcome set (COS) for the future reporting of OAB trials [34].

Previous simulation studies in multivariate meta-analysis have shown that in situations when there are a large proportion of missing data, a multivariate approach results in increased borrowing of information and increased precision in treatment effect estimates [6, 16]. Thus, multivariate methods are of most use in scenarios with a larger proportion of missing data, and less use in scenarios with complete data. In a Bayesian framework, with non-informative prior distributions specified for hyper-parameters (as described in this paper), it has previously been shown that the total number of missing values must not exceed ((*J*−1)×*N*)−((*J*−1)+*N*), for *J* interventions and *N* outcomes. In situations with which the total number of missing values exceeds this number, the hyper-parameters will become unidentifiable and informative prior distributions will be required to improve model convergence [7].

To ameliorate the impact of outcome reporting bias, the correlation between outcomes were used to obtain a predictive value for missing data. In order to achieve this, an assumption of constant relative effectiveness across outcomes was assumed for the basic parameters of the pooled treatment effect estimates, *d*_(1*j*)*l*_ and $d_{(1_{1}j_{m})l}$, as described in Eqs. (6) and (10) for MVNMA and MVNMA incorporating class effects, respectively. If interest lies in the difference between active interventions, this may be a strong assumption as the outcome-specific effect, *γ*_*l*_, will cancel. For example, the relative treatment effects of intervention A relative to intervention B obtained from MVNMA are expressed in terms of the basic parameters such that *d*_*AB*_=(*d*_(1*B*)*l*_−*d*_(1*A*)*l*_)∼Normal(*α*_*B*_−*α*_*A*_,2*ζ*^2^). Thus, in these situations, alternative methods should be explored. An assumption of constant relative effectiveness across outcomes is of less importance if interest lies in the relative rankings of the interventions, as these are calculated based on the basic parameters, *d*_(1*j*)*l*_.

As with all random effects models, in situations when the deviation of treatment effect profiles across outcomes, *ζ*, is large, it may not be sensible to combine data across all outcomes. In this scenario, we encourage the use of sensitivity analyses to; i) assess the impact of potentially outlying observations in univariate and multivariate analyses, and ii) assess the difference in mean treatment effects, and variability in mean treatment effects across outcomes, using a series of univariate analyses. Reducing the model to a bivariate or univariate analysis in sensitivity analyses will allow investigation of the impact of outcomes on the magnitude of *ζ*, as well as the influence of missing outcome data on the robustness of the overall conclusions.

In this example, the outcome-specific effect, *γ*_*l*_, was assumed to be constant across all interventions. Further work could extend this model to incorporate the exchangeability of outcome-specific effects within classes of interventions, such that $\gamma _{l_{m}}\sim \textit {Normal}(\kappa _{m},\Psi _{m}^{2})$, where *κ*_*m*_ denotes the pooled outcome effect for the *m*^*th*^ class of interventions, and $\Psi _{m}^{2}$ denotes the class-specific between intervention variance. However this approach is likely to substantially increase the number of parameters to be estimated in the model and could lead to computational difficulties. Furthermore, if there is evidence to suggest that there is a disparity in outcome-specific effects between classes, the assumption of homogeneous between-study correlations is also unlikely to be satisfied and alternative parameterisations of the between-study covariance matrix would need to be considered.

One limitation of implementing MVNMA in WinBUGS, is the difficultly in calculating deviance statistics for the assessment of model fit and comparison. Residual deviance could be calculated by monitoring the estimated true treatment effects, ***θ***_***ij***_, for each study *i*=1,...,*n*_*s*_ and intervention *j*=1,...,*n*_*t*_, and calculating the difference of these true treatment effects relative to the observed treatment effects, ***Y***_***ij***_, using the following equation: total residual deviance = $\sum _{i=1,j=1}^{i=n_{s},j=n_{t}}(\boldsymbol {Y_{ij}}-\boldsymbol {\theta _{ij}})^{2}\boldsymbol {S_{ij}}$, where ***S***_***ij***_ denotes the treatment-specific within-study covariance matrix. In this paper, the within-study correlations were incorporated at the treatment-arm level in order to appropriately account for multi-arm trials [7], thus the within-study model was parametrised at the arm-level whereas the between-study model, with which ***θ***_***ij***_ is estimated, was parametrised at the study-level. This makes calculation of residual deviances more difficult, especially in the presence of large amounts of missing data. In the OAB example, there were a large number of observed data-points with intermittently missing data, therefore, such an approach would be both computational-, and time-intensive. Further work is needed to explore re-parameterisation of MVNMA models, and alternative methods in order to adequately assess model fit and comparison.

In this example, use of a MVNMA incorporating class effects was illustrated using the three cardinal symptoms of OAB (incontinence, voiding, and urgency). However, interest lies in both efficacy and safety outcomes. Incorporating additional outcomes results in an exponential increase in the number of parameters to be estimated in the model. This is particularly true for estimation of the parameters involved in both the within-study, and between-study covariance matrix. This substantial increase in the number of parameters can often result in computational difficulties for complex multivariate models such MVNMAs. Furthermore, estimating the within-study correlation structures can be particularly difficult for mixed [9] and binary outcomes [18]. This is because an analytic solution is not possible [18]. A further limitation of using MVNMA for imputing missing data with mixed outcomes, is the assumption that intervention effects are exchangeable across outcomes. This assumption may not be reasonable if the outcomes differ in an important way, e.g, if the outcomes were measured on different scales. For example, binary outcomes on a log-odds scale and continuous outcomes on a mean difference scale will differ in terms of the uncertainty with which they were estimated.

In this analysis, a homogeneity of correlations assumption was used to simplify the number of parameters in the model and to aid computation. In situations with which there are fewer interventions, and more information for each treatment comparison, it may be desirable to incorporate treatment-specific between-study correlations. It may also be desirable to incorporate treatment-specific within-study correlations, however, data for every pair of treatment comparisons may be difficult to obtain, and thus within-study correlations may be difficult to estimate. In this example, the within-study correlations were estimated from individual patient data obtained from the RELAX trial [37]. Uncertainty in estimating the within-study correlations needs to be further accounted for. For continuous outcomes that follow a multivariate normal distribution, it would be possible to obtain estimates of the within-study correlations directly from the covariance matrix [9]. Estimates of the within-study correlations, together with their uncertainty, could be incorporated in to the MVNMA model by applying prior distributions to the within-study correlation parameters, *pw*_*xy*_, using a bootstrapping method [9].

## Conclusions

Accounting for the correlation between outcomes, MVNMAs incorporating class effects allowed for the evaluation of all interventions across all outcome measures. In the OAB example, including this additional information changed the overall clinical conclusions. Estriol 1mg intravesically was ranked in the top 10 interventions for the management of all cardinal symptoms of OAB, and sacral nerve stimulation was found to be the most effective intervention for reducing urgency. Borrowing information across outcomes using MVNMAs generally increased the precision in the treatment effect estimates. This precision was further increased by incorporating a hierarchical structure where similarities between interventions that belong to the same class of interventions were also accounted for. Overall, MVNMAs can provide a flexible framework to model a mixture of outcomes and are of most use in situations when there is a large proportion of missing data, ameliorating the impact of outcome reporting bias. MVNMAs incorporating class effects, applied judiciously, have proven to be a useful methodology. These methods have the potential to aid health technology assessment decision making by increasing precision in treatment effect estimates, as well as allowing for the complete evaluation of all outcomes and interventions of interest for multi-morbid, or syndromic conditions.

## Supplementary information

**Additional file 1** Treatment codes.

**Additional file 2** Multi-arm correction.

**Additional file 3** Estimating between-study and within-study correlations.

**Additional file 4** WinBUGS code.

**Additional file 5** Univariate network meta-analyses results for change from baseline in incontinence, voiding and urgency episodes.

**Additional file 6** Treatment profiles obtained from univariate network meta-analyses for change from baseline in incontinence, voiding and urgency episodes.

**Additional file 7** Convergence diagnostics.

**Additional file 8** Sensitivity analyses.

## Data Availability

The dataset analysed during this study is available from the corresponding author on reasonable request.

## Abbreviations

CrI: Credible interval
HTA: Health technology assessment
MVNMA: Multivariate network meta-analysis
NMA: Network meta-analysis
OAB: Overactive bladder
