# Supplementary material for: Multivariate network meta-analysis incorporating class effects
Source: BMC Med Res Methodol. 2020 Jul 8;20:184. doi: 10.1186/s12874-020-01025-8 (PMC7341581; doi:10.1186/s12874-020-01025-8)
Supplement: Supplementary file 1 — Additional file 1 Treatment codes. [file 12874_2020_1025_MOESM1_ESM.pdf]

Additional file 1 – Treatment codes

Code	Intervention	Code	Intervention
[1]	Placebo	[71]	Reflexology
[2]	Control	[72]	OnaBoNT-A 100u trigone sparing
[3]	Sham therapy	[73]	OnaBoNT-A 200U trigone sparing
[4]	Tolterodine ER 4mg q.d	[74]	OnaBoNTA 50u trigone sparing
[5]	Tolterodine IR 2mg b.i.d	[75]	OnaBoNTA 150u trigone sparing
[6]	Tolterodine IR 1mg b.i.d	[76]	OnaBoNTA 300u trigone sparing
[7]	Oxybutynin IR 5mg t.i.d	[77]	Solifenacin ER 5 - 10mg q.d + BT
[8]	Oxybutynin ER 10mg q.d	[78]	OnaBoNT-A 100u bladder body + trigone
[9]	Oxybutynin ER 15mg q.d	[79]	OnaBoNT-A 100u bladder base + trigone
[10]	Oxybutynin trandermal 3.9mg/day	[80]	Electrostimulation
[11]	Oxybutynin transdermal 1.3mg/day	[81]	Sacral nerve stimulation
[12]	Oxybutynin transdermal 2.6mg/day	[82]	Tarafenacin 0.4mg q.d
[13]	Oxybutynin chloride topical gel 1g/day	[83]	Percutaneous tibial nerve stimulation
[14]	Oxybutynin intravesically 5mg t.i.d	[84]	Pelvic Floor Muscle Training (PFMT)/Physiotherapy
[15]	Oxbutynin patch 73.5mg	[85]	Bladder Training (BT)/Behaviour Therapy
[16]	Oxybutynin vaginal ring 4mg q.d	[86]	Oxybutynin ER 5-30mg q.d + BT
[17]	Oxybutynin vaginal ring 6mg q.d	[87]	Tolterodine ER 4mg q.d + BT
[18]	Oxybutynin IR 5mg b.i.d	[88]	Darifenacin 7.5 - 15mg q.d + BT
[19]	Oxybutynin IR 3mg t.i.d	[89]	PFMT + BT
[20]	Oxybutynin ER 2.5mg q.d	[90]	Tarafenacin 0.2mg q.d
[21]	Oxybutynin IR 2.5mg t.i.d	[91]	Trospium chloride IR 15mg t.i.d + Physiotherapy
[22]	Oxybutynin ER 5 - 30mg/day	[92]	Oxybutynin ER 2.5mg q.d + BT
[23]	Oxybutynin IR 5 - 20mg	[93]	Tolterodine IR 2mg b.i.d + BT
[24]	Oxybutynin IR 2.5 - 5mg b.i.d	[94]	Tolterodine + BT
[25]	Fesoterodine ER 4mg q.d	[95]	Tolterodine IR 2mg b.i.d + PFMT
[26]	Fesoterodine ER 8mg q.d	[96]	Tolterodine ER 4mg q.d + Neurostimulation
[27]	Fesoterodine ER 4 - 8mg q.d	[97]	Electrostimulation + PFMT + BT
[28]	Terodiline 25mg b.i.d	[98]	Oxybutynin IR 2.5mg b.i.d + Salivary pastilles
[29]	Solifenacin ER 5mg q.d	[99]	Tolterodine ER 2mg b.i.d + oestrogen 0.625mg 2xwk
[30]	Solifenacin ER 10mg q.d	[100]	Solifenacin/trospium + placebo injection
[31]	Solifenacin ER 5 - 10mg q.d	[101]	Tolterodine 2mg + Pilocarpine 9mg b.i.d
[32]	Solifenacin ER 2.5mg q.d	[102]	Pregabalin 150mg b.i.d + Tolterodine ER 4mg q.d
[33]	Solifenacin ER 20mg q.d	[103]	Pregabalin 75mg b.i.d + Tolterodine ER 2mg q.d
[34]	Solifenacin ER 5 - 15mg q.d	[104]	Darifenacin ER 7.5 - 15mg q.d
[35]	Imidafenacin 0.05mg b.i.d	[105]	Tolterodine
[36]	Imidafenacin 0.1mg b.i.d	[106]	Oxybutynin 20mg intravesically q.d
[37]	Imidafenacin 0.25mg b.i.d	[107]	Serlopitant 0.25mg q.d
[38]	Darifenacin ER 30mg q.d	[108]	Serlopitant 1mg q.d
[39]	Darifenacin ER 7.5mg q.d	[109]	Serlopitant 4mg q.d
[40]	Darifenacin ER 15mg q.d.	[110]	Netupitant 50mg q.d
[41]	Propiverine ER 20mg q.d	[111]	Netupitant 100mg q.d
[42]	Propiverine ER 30mg q.d	[112]	Netupitant 200mg q.d
[43]	Propiverine IR 15mg b.i.d	[113]	Propantheline Bromide 15mg t.i.d
[44]	Trospium chloride ER 60mg q.d	[114]	Naftopidil 25mg q.d
[45]	Trospium chloride IR 20mg b.i.d	[115]	Solifenacin ER 5mg q.d + Naftopidil 25mg q.d
[46]	Trospium chloride IR 15mg t.i.d	[116]	Propiverine IR 15mg t.i.d
[47]	Trospium chloride IR 45mg t.i.d	[117]	Propiverine IR 30mg b.i.d
[48]	Mirabegron IR 100mg b.i.d	[118]	Propiverine IR 45mg t.i.d
[49]	Mirabegron IR 150mg b.i.d	[119]	Propiverine ER 60mg q.d
[50]	Mirabegron ER 25mg q.d	[120]	Fesoterodine IR 4mg b.i.d
[51]	Mirabegron ER 50mg q.d	[121]	Fesoterodine IR 8mg b.id
[52]	Mirabegron ER 100mg q.d	[122]	Fesoterodine IR 12mg b.i.d
[53]	Mirabegron ER 200mg q.d	[123]	Trospium 30mg/day + Solifenacin 10mg/day (cyclic)
[54]	Solabegron IR 50mg b.i.d	[124]	Tolterodine IR 0.5mg b.i.d
[55]	Solabegron IR 125mg b.i.d	[125]	Electromagnetic stimulation
[56]	Cizolirtine citrate 200mg b.i.d	[126]	Trospium 30mg/day + Solifenacin 10mg/day (continuous)
[57]	Cizolirtine citrate 400mg b.i.d	[127]	Estradiol 1mg intravaginally
[58]	ZD0947IL 25mg/day	[128]	Estradiol 3mg intravaginally
[59]	ONO-8539 30mg b.i.d	[129]	Lidocaine gel 2x6ml
[60]	ONO-8539 100mg b.i.d	[130]	Emepronium Bromide IR 200mg t.i.d
[61]	ONO-8539 300mg b.i.d	[131]	Estriol 1mg intravesically
[62]	Pregabalin 150mg b.i.d	[132]	Vaginal oestrogen cream 1.25mg/day
[63]	Emepronium bromide ER 200mg q.d	[133]	Electrostimulation + vaginal oestrogen cream 1.25mg/day
[64]	Flavoxate chloride 200mg q.d	[134]	Oxybutynin gel 84mg/day
[65]	Duloxetine 40mg b.i.d	[135]	Oxybutynin gel 56mg/day
[66]	Duloxetine 60mg b.i.d	[136]	Sacral Nerve Stimulation + Tolterodine ER 2mg q.d
[67]	Resiniferatoxin 50nM	[137]	Tolterodine ER 2mg q.d
[68]	Estradiol 25mg	[138]	Lipo-BoNTA 200U
[69]	Elocalcitol 150mg	[139]	Trospium 60mg/day + Solifenacin 20mg/day (cyclic)
[70]	Elocalcitol 75mg	[140]	Tolterodine IR 4mg b.i.d
