# Supplementary material for: Multivariate network meta-analysis incorporating class effects
Source: BMC Med Res Methodol. 2020 Jul 8;20:184. doi: 10.1186/s12874-020-01025-8 (PMC7341581; doi:10.1186/s12874-020-01025-8)
Supplement: Supplementary file 2 — Additional file 2 Multi-arm correction. [file 12874_2020_1025_MOESM2_ESM.pdf]

Additional file 2 — Multi-arm correction

To account for the correlation between study-specific treatment comparisons for multi-arm trials, i.e. $k > 2$, the elements of $\boldsymbol{\delta_{i,bk}}$ are expressed in terms of the following marginal and conditional distributions:

$$\begin{pmatrix} \delta_{i,b2(1)} \\ \delta_{i,b2(2)} \\ \delta_{i,b2(3)} \end{pmatrix} \sim \text{MVN}\left(\begin{pmatrix} d_{t_{ib}t_{i2}(1)} = d_{1,t_{ik}(1)} - d_{1,t_{ib}(1)} \\ d_{t_{ib}t_{i2}(2)} = d_{1,t_{ik}(2)} - d_{1,t_{ib}(2)} \\ d_{t_{ib}t_{i2}(3)} = d_{1,t_{ik}(3)} - d_{1,t_{ib}(3)} \end{pmatrix}, \boldsymbol{\Sigma} \right)$$

where

$$\boldsymbol{\Sigma} = \begin{pmatrix} \sigma_1^2 & \rho_{12}\sigma_1\sigma_2 & \rho_{13}\sigma_1\sigma_3 \\ . & \sigma_2^2 & \rho_{23}\sigma_2\sigma_3 \\ . & . & \sigma_3^2 \end{pmatrix}$$

and for $k = 3 \ldots na_i$, the kth conditional distribution is defined by:

$$\begin{pmatrix} \delta_{i,bk(1)} \\ \delta_{i,bk(2)} \\ \delta_{i,bk(3)} \end{pmatrix} \mid \begin{pmatrix} \begin{pmatrix} \delta_{i,b2(1)} \\ \delta_{i,b2(2)} \\ \delta_{i,b2(3)} \end{pmatrix} \\ \vdots \\ \begin{pmatrix} \delta_{i,b(k-1)(1)} \\ \delta_{i,b(k-1)(2)} \\ \delta_{i,b(k-1)(3)} \end{pmatrix} \end{pmatrix} \sim \text{MVN}$$

$$\left(\begin{pmatrix} \left(d_{t_{ib}t_{ik}(1)} = d_{1,t_{ik}(1)} - d_{1,t_{ib}(1)}\right) + \frac{1}{k-1}\sum_{q=1}^{k-1}\left[\delta_{i,1q(1)} - \left(d_{1,t_{iq}(1)} - d_{1,t_{ib}(1)}\right)\right] \\ \left(d_{t_{ib}t_{ik}(2)} = d_{1,t_{ik}(2)} - d_{1,t_{ib}(2)}\right) + \frac{1}{k-1}\sum_{q=1}^{k-1}\left[\delta_{i,1q(2)} - \left(d_{1,t_{iq}(2)} - d_{1,t_{ib}(2)}\right)\right] \\ \left(d_{t_{ib}t_{ik}(3)} = d_{1,t_{ik}(3)} - d_{1,t_{ib}(3)}\right) + \frac{1}{k-1}\sum_{q=1}^{k-1}\left[\delta_{i,1q(3)} - \left(d_{1,t_{iq}(3)} - d_{1,t_{ib}(3)}\right)\right] \end{pmatrix}, \frac{k}{2(k-1)}\boldsymbol{\Sigma}_{3\times 3} \right)$$

where

$$\boldsymbol{\Sigma}_{3\times 3} = \begin{pmatrix} \begin{pmatrix} \sigma_1^2 & \rho_{12}\sigma_1\sigma_2 & \rho_{13}\sigma_1\sigma_3 \\ . & \sigma_2^2 & \rho_{23}\sigma_2\sigma_3 \\ . & . & \sigma_3^2 \end{pmatrix} & \frac{1}{2}\begin{pmatrix} \sigma_1^2 & \rho_{12}\sigma_1\sigma_2 & \rho_{13}\sigma_1\sigma_3 \\ . & \sigma_2^2 & \rho_{23}\sigma_2\sigma_3 \\ . & . & \sigma_3^2 \end{pmatrix} & \frac{1}{2}\begin{pmatrix} \sigma_1^2 & \rho_{12}\sigma_1\sigma_2 & \rho_{13}\sigma_1\sigma_3 \\ . & \sigma_2^2 & \rho_{23}\sigma_2\sigma_3 \\ . & . & \sigma_3^2 \end{pmatrix} \\ \frac{1}{2}\begin{pmatrix} \sigma_1^2 & \rho_{12}\sigma_1\sigma_2 & \rho_{13}\sigma_1\sigma_3 \\ . & \sigma_2^2 & \rho_{23}\sigma_2\sigma_3 \\ . & . & \sigma_3^2 \end{pmatrix} & \begin{pmatrix} \sigma_1^2 & \rho_{12}\sigma_1\sigma_2 & \rho_{13}\sigma_1\sigma_3 \\ . & \sigma_2^2 & \rho_{23}\sigma_2\sigma_3 \\ . & . & \sigma_3^2 \end{pmatrix} & \frac{1}{2}\begin{pmatrix} \sigma_1^2 & \rho_{12}\sigma_1\sigma_2 & \rho_{13}\sigma_1\sigma_3 \\ . & \sigma_2^2 & \rho_{23}\sigma_2\sigma_3 \\ . & . & \sigma_3^2 \end{pmatrix} \\ \frac{1}{2}\begin{pmatrix} \sigma_1^2 & \rho_{12}\sigma_1\sigma_2 & \rho_{13}\sigma_1\sigma_3 \\ . & \sigma_2^2 & \rho_{23}\sigma_2\sigma_3 \\ . & . & \sigma_3^2 \end{pmatrix} & \frac{1}{2}\begin{pmatrix} \sigma_1^2 & \rho_{12}\sigma_1\sigma_2 & \rho_{13}\sigma_1\sigma_3 \\ . & \sigma_2^2 & \rho_{23}\sigma_2\sigma_3 \\ . & . & \sigma_3^2 \end{pmatrix} & \begin{pmatrix} \sigma_1^2 & \rho_{12}\sigma_1\sigma_2 & \rho_{13}\sigma_1\sigma_3 \\ . & \sigma_2^2 & \rho_{23}\sigma_2\sigma_3 \\ . & . & \sigma_3^2 \end{pmatrix} \end{pmatrix}$$
