# Supplementary material for: Multivariate network meta-analysis incorporating class effects
Source: BMC Med Res Methodol. 2020 Jul 8;20:184. doi: 10.1186/s12874-020-01025-8 (PMC7341581; doi:10.1186/s12874-020-01025-8)
Supplement: Supplementary file 3 — Additional file 3 Estimating between-study and within-study correlations. [file 12874_2020_1025_MOESM3_ESM.pdf]

Additional file 3 — Estimating between-study and within-study correlations

The between-study variance-covariance matrix, $\mathbf{\Sigma}$ requires specification of a prior distribution which ensures that it is non-negative definite [38]. In a multivariate meta-analysis setting, the spherical parameterisation technique based on Cholesky decomposition [27] has previously been adopted [7, 18, 38, 39] using the following decomposition described by [40]:

$$\mathbf{\Sigma} = \boldsymbol{V}^{\mathbf{1/2}}\boldsymbol{R}\boldsymbol{V}^{\mathbf{1/2}}$$

This approach is used to express the between-study variance-covariance matrix, $\mathbf{\Sigma}$ in terms of a diagonal matrix of standard deviations, $\boldsymbol{V}^{\mathbf{1/2}}$, and positive-definite matrix of correlations, $\boldsymbol{R}$. Here, the elements of $\boldsymbol{V}^{\mathbf{1/2}}$ represent the between-study standard deviations of $\mathbf{\Sigma}$ and were assigned Uniform(0,2) prior distributions. $\boldsymbol{R}$ represents the correlation matrix where the diagonal elements are set to 1, and the off-diagonal elements contain the set of correlation coefficients. Estimating all between-study correlation parameters can contribute a large number of parameters to the covariance matrix which can result in computational difficulties [18]. Thus, reducing the number of correlation parameters is often desirable [18]. In addition to assuming homogeneous between-study standard deviations across treatment comparisons, which is a common assumption of NMAs [2], it is also possible to assume that the between-study correlations are equal [7, 18]. This approach assumes that if several, independent, multivariate meta-analyses were conducted on the same outcomes, each with a different set of k versus b treatment comparisons, then the between-study correlations, ρ_{xy}, would be the same across the different sets of treatment comparisons:

$$\boldsymbol{R} = \begin{bmatrix} 1 & \rho_{12} & \rho_{13} \\ . & 1 & \rho_{23} \\ . & . & 1 \end{bmatrix}$$

Using Cholesky decomposition, it has been shown that $\boldsymbol{R}$ can be written in terms of an upper triangular matrix, $\boldsymbol{L}$, [18, 39] such that:

$$\boldsymbol{R} = \boldsymbol{L}^{\boldsymbol{T}}\boldsymbol{L}$$

With this notation, the spherical parameterisation technique can be used to express $\boldsymbol{R}$ in terms of the elements of $\boldsymbol{L}$ using sine and cosine functions [39], such that:

$$\boldsymbol{L} = \begin{pmatrix} 1 & \cos(\varphi_{12}) & \cos(\varphi_{13}) \\ 0 & \sin(\varphi_{12}) & \sin(\varphi_{13})\cos(\varphi_{23}) \\ 0 & 0 & \sin(\varphi_{13})\sin(\varphi_{23}) \end{pmatrix}$$

To ensure that the elements of the correlation matrix $\boldsymbol{R}$ are constrained between (-1,1), and that positive semi-definiteness of the between-study variance-covariance matrix is satisfied, Uniform(0,π) prior distributions were specified for the spherical

parameters, φ_{xy}, where $\pi = 3.142$ [7, 39].

The within-study correlations are very rarely reported in clinical trials of multiple outcomes, and estimating these correlations can be difficult. In the MVNMA models described above, the treatment-specific within-study covariance matrices $\boldsymbol{S_{ij}}$ (for MVNMA) and $\boldsymbol{S_{ij_m}}$ (for MVNMA incorporating class effects) are assumed known, and thus the within-study correlations are also assumed known [17]. For the purpose of these analyses, within-study correlations were calculated using individual patient data obtained from the RELAX trial [37]. To obtain within-study correlations, Pearson correlation coefficients were calculated between the outcomes of interest [7], using patient reported bladder diaries documenting urinary incontinence, voiding and urgency episodes. To incorporate the uncertainty in estimating the within-study correlations, prior distributions could be specified on the parameters ρw_{xy} using a bootstrapping method [9]. Assuming that all outcomes follow a common multivariate normal distribution, it would be possible to directly obtain within-study correlations from the covariance matrix [9]. However, to aid model computation in this example, within study correlations were assumed known.
