# Supplementary material for: Multivariate network meta-analysis incorporating class effects
Source: BMC Med Res Methodol. 2020 Jul 8;20:184. doi: 10.1186/s12874-020-01025-8 (PMC7341581; doi:10.1186/s12874-020-01025-8)
Supplement: Supplementary file 4 — Additional file 4 WinBUGS code. [file 12874_2020_1025_MOESM4_ESM.pdf]

Additional file 4 — WinBUGS code

```
Model {

# Generating missing SEs
#======================
	for(i in 1:N1){
	for (m in 1:no){

change_var[i,m] <- ((pow(b_sd_star[i,m],2) + pow(f_sd_star[i,m],2) -
2*rho.star[i,m]*b_sd_star[i,m]*f_sd_star[i,m])*equals(ind_c_miss[i,m],1)) +
(pow(c_sd[i,m],2)*(equals(ind_c_miss[i,m],0)))

change_sd[i,m] <- sqrt(change_var[i,m])
se[i,m] <- change_sd[i,m]/sqrt(numinclanalysis[i,m])

b_sd_star[i,m] <- 0*equals(ind_b_miss[i,m],1) +
(b_sd[i,m])*(equals(ind_b_miss[i,m],0))

b_sd[i,m] ~ dunif(0,15)
f_sd[i,m] ~ dunif(0, 15)
c_sd[i,m] ~ dunif(0,25)

z[i,m]~ dnorm(z.star[i,m],z.prec[m])
rho.star[i,m]<- (exp(2*z[i,m])-1)/(exp(2*z[i,m])+1)
}

f_sd_star[i,1] <- (-0.011+(0.835*b_sd[i,1]))*(equals(ind_f_miss[i,1],1)) +
(f_sd[i,1])*(equals(ind_f_miss[i,1],0))
f_sd_star[i,2] <- (1.44+(0.42*b_sd[i,2]))*(equals(ind_f_miss[i,2],1)) +
(f_sd[i,2])*(equals(ind_f_miss[i,2],0))
f_sd_star[i,3] <- (0.161+(0.832*b_sd[i,3]))*(equals(ind_f_miss[i,3],1)) +
(f_sd[i,3])*(equals(ind_f_miss[i,3],0))

z.star[i,1] ~ dnorm(0.67, 12.76)
z.star[i,2] ~ dnorm(0.74, 10.41)
z.star[i,3] ~ dnorm(0.51, 10.41)
}

z.se[1] <- 1/(sqrt(49-3))
z.prec[1]<- pow(z.se[1],-2)
z.se[2] <- 1/(sqrt(44-3))
z.prec[2] <- pow(z.se[2],-2)
z.se[3] <- 1/(sqrt(21-3))
z.prec[3] <- pow(z.se[2],-2)
```

```
#Likelihood for arm level data
#====================
for(i in 1:N1){
#tmp1[i] <- studyid[i]                          # study id not used in the model
# multivariate likelihood
y[i,1:3] ~ dmnorm(mean.y[study[i],arm[i],1:3],omega[i,,])
omega[i,1:3,1:3] <- inverse(cov.mat[i,,])              # within-study precision matrix

    #elements of within-study covariance matrix
    cov.mat[i,1,1] <-  pow(se[i,1],2)
    cov.mat[i,2,2] <-  pow(se[i,2],2)
    cov.mat[i,3,3] <-  pow(se[i,3],2)
    cov.mat[i,1,2] <-  se[i,1]*se[i,2]*0.4564
    cov.mat[i,1,3] <-  se[i,1]*se[i,3]*0.6178
    cov.mat[i,2,3] <-  se[i,2]*se[i,3]*0.6763
    cov.mat[i,2,1] <-  cov.mat[i,1,2]
    cov.mat[i,3,1] <-  cov.mat[i,1,3]
    cov.mat[i,3,2] <-  cov.mat[i,2,3]
              }

for(j in 1:ns){
   for(k in 1:na2[j]) {
      for(m in 1:no){
mean.y[j,k,m] <- mu[j,m] + delta[j,k,m]         # define study-specific treatment effects
      }
   }
 }

 #Random effects between-study model
 #===============================
for(j in 1:ns)   {
    tmp3[j] <- s[j]

for(m in 1:no)          {
      delta[j,1,m] <-0                      #delta in control arm set to zero for all outcomes
      w[j,1,m] <-0                        #multi-arm adjustment in control group set to zero
       }

for(k in 2:na2[j])       {
#trial specific treatment effects drawn from multivariate normal distribution
        delta[j,k,1:no] ~ dmnorm(md[j,k,1:no],precBK[j,k,1:no,1:no])
  for(m in 1:no){
md[j,k,m] <-  (d[m,t[j,k]] - d[m,t[j,1]])+ sw[j,k,m]            #consistency equations
w[j,k,m] <- delta[j,k,m] - (d[m,t[j,k]] - d[m,t[j,1]])           #multi-arm adjustment
sw[j,k,m] <- sum(w[j,1:k-1,m])/(k-1)
```

```
    for(mm in 1:no)    {
                          precBK[j,k,m,mm] <- prec[m,mm]*2*(k-1)/k
                     }
 }}}

# Reference treatment effect set to zero
 d[1,1] <- 0
 d[2,1] <- 0
 d[3,1] <- 0

 # Between-study covariance matrix
 prec[1:no,1:no] <- inverse(sigma[,])
 sd.se~ dunif(0, 2)
 #prec.se.star~dgamma(0.01,0.01)
 #sd.se<-1/sqrt(prec.se.star)
 #sd.se~dnorm(0,1)I(0,)

  for(m in 1:no) {
    prec.se[m] <- pow(sd.se,-2)
    sigma[m,m] <- pow(sd[m],2)
    sd[m] ~ dunif(0, 2)
  for(j in 1:ns){
      mu[j, m] ~ dnorm(0,0.001)
         }}

#spherical parameterization
      pi <- 3.1415
    for(i in 1:2) {
       for(j in (i+1):no) {
          sigma[i,j] <- rho[i,j]*sd[i]*sd[j]
          sigma[j,i] <- sigma[i,j]
          g[j,i] <- 0
          a[i,j] ~ dunif(0, pi)
          rho[i,j] <- inprod(g[,i], g[,j])
    }}

 g[1,1] <- 1
 g[1,2] <- cos(a[1,2])
 g[2,2] <- sin(a[1,2])
 g[1,3] <- cos(a[1,3])
 g[2,3] <- sin(a[1,3])*cos(a[2,3])
 g[3,3] <- sin(a[1,3])*sin(a[2,3])

# Borrowing information across outcomes
#===========================================
for(k in 2: nt){
```

```
for(m in 1:no) {
	meanD[m,k-1] <- alpha[k-1] + gamma[m]		#outcome and treatment effects
	d[m,k] ~ dnorm(meanD[m,k-1], prec.btw)}}			#treatment effects

for(m in 1:no) {gamma[m] ~ dnorm(0, 0.01) }
#for(k in 1:(nt-1)) {alpha[k] ~ dnorm(0, 0.001) }
for(k in 1:2){alpha[k]~ dnorm(D.d[1], D.d.prec[1])}
for(k in 3:5){alpha[k]~ dnorm(D.d[2], D.d.prec[2])}
for(k in 6:23){alpha[k]~ dnorm(D.d[3], D.d.prec[3])}
for(k in 24:26){alpha[k]~ dnorm(D.d[4], D.d.prec[4])}
alpha[27]~ dnorm(D.d[5], D.d.prec[5])
for(k in 28:30){alpha[k]~ dnorm(D.d[6], D.d.prec[6])}
for(k in 31:32){alpha[k]~ dnorm(D.d[7], D.d.prec[7])}
alpha[33]~ dnorm(D.d[6], D.d.prec[6])
for(k in 34:36){alpha[k]~ dnorm(D.d[8], D.d.prec[8])}
for(k in 37:39){alpha[k]~ dnorm(D.d[9], D.d.prec[9])}
for(k in 40:42){alpha[k]~ dnorm(D.d[10], D.d.prec[10])}
alpha[43]~ dnorm(D.d[11], D.d.prec[11])
alpha[44]~ dnorm(D.d[12], D.d.prec[12])
for(k in 45:46){alpha[k]~ dnorm(D.d[11], D.d.prec[11])}
for(k in 47:52){alpha[k]~ dnorm(D.d[13], D.d.prec[13])}
for(k in 53:54){alpha[k]~ dnorm(D.d[14], D.d.prec[14])}
for(k in 55:56){alpha[k]~ dnorm(D.d[15], D.d.prec[15])}
alpha[57]~ dnorm(D.d[16], D.d.prec[16])
for(k in 58:60){alpha[k]~ dnorm(D.d[17], D.d.prec[17])}
alpha[61]~ dnorm(D.d[18], D.d.prec[18])
alpha[62]~ dnorm(D.d[19], D.d.prec[19])
alpha[63]~ dnorm(D.d[20], D.d.prec[20])
alpha[64]~ dnorm(D.d[21], D.d.prec[21])
alpha[65]~ dnorm(D.d[3], D.d.prec[3])
alpha[66]~ dnorm(D.d[22], D.d.prec[22])
alpha[67]~ dnorm(D.d[23], D.d.prec[23])
for(k in 68:69){alpha[k]~ dnorm(D.d[24], D.d.prec[24])}
alpha[70]~ dnorm(D.d[25], D.d.prec[25])
for(k in 71:72){alpha[k]~ dnorm(D.d[26], D.d.prec[26])}
alpha[73]~ dnorm(D.d[3], D.d.prec[3])
alpha[74]~ dnorm(D.d[27], D.d.prec[27])
alpha[75]~ dnorm(D.d[28], D.d.prec[28])
alpha[76]~ dnorm(D.d[29], D.d.prec[29])
for(k in 77:78){alpha[k]~ dnorm(D.d[26], D.d.prec[26])}
alpha[79]~ dnorm(D.d[30], D.d.prec[30])
alpha[80]~ dnorm(D.d[31], D.d.prec[31])
alpha[81]~ dnorm(D.d[23], D.d.prec[23])
alpha[82]~ dnorm(D.d[32], D.d.prec[32])
alpha[83]~ dnorm(D.d[33], D.d.prec[33])
alpha[84]~ dnorm(D.d[34], D.d.prec[34])
```

```
alpha[85]~ dnorm(D.d[35], D.d.prec[35])
alpha[86]~ dnorm(D.d[36], D.d.prec[36])
alpha[87]~ dnorm(D.d[23], D.d.prec[23])
alpha[88]~ dnorm(D.d[37], D.d.prec[37])
alpha[89]~ dnorm(D.d[38], D.d.prec[38])
alpha[90]~ dnorm(D.d[39], D.d.prec[39])
alpha[91]~ dnorm(D.d[35], D.d.prec[35])
alpha[92]~ dnorm(D.d[36], D.d.prec[36])
alpha[93]~ dnorm(D.d[10], D.d.prec[10])
alpha[94]~ dnorm(D.d[40], D.d.prec[40])
alpha[95]~ dnorm(D.d[41], D.d.prec[41])
alpha[96]~ dnorm(D.d[42], D.d.prec[42])
alpha[97]~ dnorm(D.d[43], D.d.prec[43])
alpha[98]~ dnorm(D.d[44], D.d.prec[44])
alpha[99]~ dnorm(D.d[45], D.d.prec[45])
alpha[100]~ dnorm(D.d[46], D.d.prec[46])
for(k in 101:102){alpha[k]~ dnorm(D.d[47], D.d.prec[47])}
for(k in 103:104){alpha[k]~ dnorm(D.d[10], D.d.prec[10])}
alpha[105]~ dnorm(D.d[3], D.d.prec[3])
for(k in 106:108){alpha[k]~ dnorm(D.d[48], D.d.prec[48])}
for(k in 109:111){alpha[k]~ dnorm(D.d[49], D.d.prec[49])}
alpha[112]~ dnorm(D.d[50], D.d.prec[50])
alpha[113]~ dnorm(D.d[51], D.d.prec[51])
alpha[114]~ dnorm(D.d[52], D.d.prec[52])

for(i in 1:52){
D.d[i] ~ dnorm(0, 0.001)
D.d.prec[i]<- pow(D.d.sd[i],-2)
D.d.sd[i] ~ dunif(0,2)}

 prec.btw <- pow(sd.btw,-2)
 sd.btw ~ dunif(0, 2)
#prec.btw <- pow(sd.btw,-2)
#sd.btw ~ dnorm(0,1)I(0,)
#prec.btw ~ dgamma(0.001,0.001)
#sd.btw <-1/sqrt(prec.btw)

for (m in 1:no) {
for (c in 1:(nt-1)) {
        for (k in (c+1):nt) {
                diff[m,c,k] <- (d[m,k] - d[m,c] )}}
for (k in 1:nt) {
        rk[m,k] <- rank(d[m,],k)

        best[m,k] <- equals(rk[m,k],1)}
        }}}
```
