# Supplementary material for: Multivariate network meta-analysis incorporating class effects
Source: BMC Med Res Methodol. 2020 Jul 8;20:184. doi: 10.1186/s12874-020-01025-8 (PMC7341581; doi:10.1186/s12874-020-01025-8)
Supplement: Supplementary file 5 — Additional file 5 Univariate network meta-analyses results for change from baseline in incontinence, voiding and urgency episodes. [file 12874_2020_1025_MOESM5_ESM.pdf]

Additional file 5 — Univariate network meta-analyses results for change from baseline in incontinence, voiding and urgency episodes

Table 1: Estimated posterior median difference (and 95% credible interval) in change from baseline for incontinence episodes relative to placebo obtained from univariate network meta-analysis

**Treatment**	**Code**	**Median difference† (95% CrI)**	**Rank (95% CrI)**	**p(Best)**
Sacral nerve stimulation	[81]	-8.72 (-11.33,-6.09)	1 (1,1)	1.00
OnaBoNT-A 200U trigone sparing	[73]	-2.3 (-3.16,-1.42)	3 (2,9)	0.00
Oxybutynin IR 2.5mg b.i.d + salivary pastilles	[98]	-2.2 (-4.06,-0.36)	4 (2,52)	0.00
Solifenacin/trospium + placebo injection	[100]	-1.97 (-2.96,-1.01)	5 (2,14)	0.00
Electrostimulation + PFMT + BT	[97]	-1.93 (-2.94,-0.91)	5 (2,17)	0.00
OnaBoNT-A 100u trigone sparing	[72]	-1.88 (-2.31,-1.45)	6 (3,9)	0.00
OnaBoNT-A 100u bladder body + trigone	[78]	-1.63 (-2.73,-0.54)	7 (2,38)	0.00
OnaBoNT-A 100u bladder base + trigone	[79]	-1.39 (-4,1.06)	9 (2,95)	0.00
Tolterodine ER 4mg q.d + Neurostimulation	[96]	-1.29 (-1.7,-0.89)	10 (6,19)	0.00
Oxybutynin intravesically 5mg t.i.d	[14]	-1.19 (-2.49,0.1)	11 (3,77)	0.00
Mirabegron IR 100mg b.i.d	[48]	-1.12 (-1.98,-0.25)	12 (5,59)	0.00
Oxybutynin ER 10mg q.d	[8]	-0.98 (-1.55,-0.42)	15 (7,48)	0.00
Oxybutynin IR 3mg t.i.d	[19]	-0.9 (-1.28,-0.52)	18 (9,41)	0.00
Solifenacin ER 10mg q.d	[30]	-0.88 (-1.14,-0.63)	18 (11,33)	0.00
Imidafenacin 0.25mg b.i.d	[37]	-0.82 (-1.43,-0.21)	21 (8,61)	0.00
Propiverine ER 30mg q.d	[42]	-0.79 (-1.31,-0.28)	23 (9,58)	0.00
Tolterodine ER 4mg q.d + BT	[87]	-0.78 (-1.45,-0.12)	23 (8,67)	0.00
Trospium chloride IR 15mg t.i.d + Physiotherapy	[91]	-0.78 (-1.71,0.16)	23 (7,77)	0.00
Oxybutynin IR 2.5 - 5mg b.i.d	[24]	-0.75 (-1.45,-0.01)	25 (8,72)	0.00
Darifenacin ER 30mg q.d	[38]	-0.74 (-1.36,-0.12)	25 (9,67)	0.00
Tolterodine ER 2mg b.i.d + Oestrogen 0.625mg twice/week	[99]	-0.75 (-1.24,-0.25)	25 (10,60)	0.00
Solifenacin ER 5 - 10mg q.d	[31]	-0.73 (-1,-0.45)	26 (14,46)	0.00
Solifenacin ER 5mg q.d	[29]	-0.71 (-0.96,-0.46)	27 (15,46)	0.00
Oxybutynin IR 5mg t.i.d	[7]	-0.71 (-1.18,-0.25)	27 (11,60)	0.00
Fesoterodine ER 8mg q.d	[26]	-0.69 (-0.89,-0.5)	28 (17,43)	0.00
Oxybutynin gel 56mg/day	[135]	-0.69 (-1.59,0.18)	28 (7,80)	0.00
Mirabegron ER 25mg q.d	[50]	-0.67 (-0.99,-0.35)	29 (15,53)	0.00
Terodiline 25mg b.i.d	[28]	-0.66 (-1.21,-0.1)	30 (11,68)	0.00
Trospium chloride ER 60mg q.d	[44]	-0.66 (-1.09,-0.24)	30 (12,61)	0.00
Cizolirtine citrate 400mg b.i.d	[57]	-0.63 (-1.17,-0.08)	32 (11,68)	0.00
Mirabegron ER 100mg q.d	[52]	-0.62 (-0.83,-0.41)	33 (20,50)	0.00
Solifenacin ER 5 - 15mg q.d	[34]	-0.61 (-1.11,-0.1)	34 (12,68)	0.00
Solabegron IR 125mg b.i.d	[55]	-0.6 (-0.94,-0.26)	34 (16,59)	0.00
Elocalcitol 75mg	[70]	-0.6 (-1.2,0.01)	34 (11,73)	0.00
Pregabalin 150mg b.i.d + Tolterodine ER 4mg q.d	[102]	-0.6 (-1.66,0.46)	34 (7,88)	0.00
Propiverine IR 15mg b.i.d	[43]	-0.57 (-1,-0.16)	36 (15,65)	0.00
Mirabegron ER 200mg q.d	[53]	-0.57 (-1.13,-0.01)	36 (12,72)	0.00
Mirabegron ER 50mg q.d	[51]	-0.57 (-0.76,-0.38)	37 (24,52)	0.00
Darifenacin ER 15mg q.d.	[40]	-0.51 (-0.97,-0.05)	41 (16,71)	0.00
Mirabegron IR 150mg b.i.d	[49]	-0.51 (-1.57,0.54)	41 (8,90)	0.00
Oxybutynin chloride topical gel 1g/day	[13]	-0.5 (-1.02,0.02)	42 (14,74)	0.00
Tolterodine ER 4mg q.d	[4]	-0.51 (-0.61,-0.4)	42 (32,52)	0.00
Fesoterodine ER 4mg q.d	[25]	-0.47 (-0.66,-0.29)	44 (30,59)	0.00
Tolterodine 2mg + Pilocarpine 9mg b.i.d	[101]	-0.48 (-0.79,-0.17)	44 (22,65)	0.00
Tolterodine IR 2mg b.i.d	[5]	-0.45 (-0.6,-0.3)	46 (34,58)	0.00
Tolterodine IR 2mg b.i.d + PFMT	[95]	-0.45 (-1.07,0.18)	46 (13,81)	0.00
Oxybutynin vaginal ring 4mg q.d	[16]	-0.44 (-1.11,0.23)	47 (12,82)	0.00
Oxybutynin vaginal ring 6mg q.d	[17]	-0.43 (-1.08,0.22)	48 (13,82)	0.00
Propiverine ER 20mg q.d	[41]	-0.41 (-0.61,-0.2)	49 (33,64)	0.00
Imidafenacin 0.1mg b.i.d	[36]	-0.4 (-0.7,-0.11)	50 (27,68)	0.00
Cizolirtine citrate 200mg b.i.d	[56]	-0.4 (-1.83,1.03)	50 (6,95)	0.00
Elocalcitol 150mg	[69]	-0.4 (-1.03,0.22)	50 (14,82)	0.00
Oxybutynin trandermal 3.9mg/day	[10]	-0.33 (-0.67,0)	54 (29,73)	0.00
Tolterodine IR 1mg b.i.d	[6]	-0.33 (-0.69,0.02)	54 (28,75)	0.00
Oxbutynin patch 73.5mg	[15]	-0.31 (-0.67,0.05)	56 (29,76)	0.00

Table 1: Estimated posterior median difference (and 95% credible interval) in change from baseline for incontinence episodes relative to placebo obtained from univariate network meta-analysis (cont.)

Fesoterodine ER 4 - 8mg q.d	[27]	-0.28 (-0.52,-0.05)	57 (39,72)	0.00
Oxybutynin gel 84mg/day	[134]	-0.29 (-1.13,0.52)	57 (13,90)	0.00
Darifenacin ER 7.5mg q.d	[39]	-0.27 (-0.84,0.3)	58 (21,85)	0.00
Oxybutynin ER 15mg q.d	[9]	-0.28 (-1.41,0.86)	58 (9,94)	0.00
Duloxetine 40mg b.i.d	[65]	-0.26 (-0.75,0.25)	59 (24,83)	0.00
PFMT + BT	[89]	-0.26 (-0.87,0.37)	59 (20,83)	0.00
Imidafenacin 0.05mg b.i.d	[35]	-0.24 (-0.77,0.27)	60 (24,84)	0.00
Solabegron IR 50mg b.i.d	[54]	-0.2 (-0.54,0.14)	62 (39,80)	0.00
Tolterodine IR 2mg b.i.d + BT	[93]	-0.14 (-0.81,0.52)	65 (22,90)	0.00
Tarafenacin 0.4mg q.d	[82]	-0.13 (-0.85,0.61)	66 (20,91)	0.00
ONO-8539 100mg b.i.d	[60]	-0.11 (-0.85,0.63)	67 (20,91)	0.00
Oxybutynin IR 2.5mg t.i.d	[21]	-0.08 (-0.39,0.23)	68 (50,83)	0.00
ZD0947IL 25mg/day	[58]	-0.1 (-0.94,0.76)	68 (17,93)	0.00
Pelvic Floor Muscle Training (PFMT)/Physiotherapy	[84]	-0.08 (-0.72,0.55)	68 (28,88)	0.00
Lipo-BoNTA 200U	[138]	-0.06 (-0.96,0.83)	69 (16,94)	0.00
Oxybutynin transdermal 1.3mg/day	[11]	-0.03 (-0.66,0.61)	71 (30,91)	0.00
Placebo	[1]	NA	72 (65,81)	0.00
Estradiol 25mg	[68]	0 (-0.38,0.38)	72 (51,87)	0.00
Pregabalin 75mg b.i.d + Tolterodine ER 2mg q.d	[103]	0 (-0.57,0.56)	72 (37,91)	0.00
Pregabalin 150mg b.i.d	[62]	0 (-0.88,0.9)	73 (19,95)	0.00
Bladder Training (BT)/Behaviour Therapy	[85]	0.02 (-0.59,0.62)	73 (37,88)	0.00
Oxybutynin ER 5 - 30mg/day	[22]	0.09 (-0.62,0.78)	76 (35,91)	0.00
Electrostimulation + vaginal oestrogen cream 1.25mg/day	[133]	0.07 (-0.45,0.59)	76 (46,91)	0.00
Electrostimulation	[80]	0.08 (-0.28,0.44)	76 (58,88)	0.00
Tarafenacin 0.2mg q.d	[90]	0.11 (-0.63,0.86)	77 (33,94)	0.00
Percutaneous tibial nerve stimulation	[83]	0.18 (-1.16,1.54)	80 (12,96)	0.00
Oxybutynin ER 2.5mg q.d + BT	[92]	0.22 (-0.94,1.4)	81 (17,96)	0.00
Oxybutynin transdermal 2.6mg/day	[12]	0.29 (-0.37,0.95)	84 (53,95)	0.00
Oxybutynin IR 5mg b.i.d	[18]	0.35 (-0.3,0.99)	85 (57,96)	0.00
Control	[2]	0.33 (-0.67,1.31)	85 (31,96)	0.00
Emepronium bromide ER 200mg q.d	[63]	0.34 (-0.33,1.01)	85 (54,96)	0.00
Flavoxate chloride 200mg q.d	[64]	0.33 (-0.35,1.01)	85 (53,96)	0.00
Reflexology	[71]	0.33 (-0.76,1.41)	85 (25,96)	0.00
Vaginal oestrogen cream 1.25mg/day	[132]	0.38 (-0.15,0.91)	86 (65,95)	0.00
Oxybutynin IR 5 - 20mg	[23]	0.46 (-0.9,1.84)	88 (18,97)	0.00
Oxybutynin ER 2.5mg q.d	[20]	0.52 (-0.5,1.55)	89 (44,97)	0.00
Trospium chloride IR 15mg t.i.d	[46]	0.52 (-0.41,1.41)	89 (51,97)	0.00
ONO-8539 300mg b.i.d	[61]	0.51 (-0.23,1.25)	89 (61,97)	0.00
ONO-8539 30mg b.i.d	[59]	0.58 (-0.18,1.33)	90 (63,97)	0.00
Resiniferatoxin 50nM	[67]	0.58 (-1.08,2.28)	90 (13,97)	0.00
Sham therapy	[3]	0.83 (-0.62,2.27)	94 (34,97)	0.00
Oxybutynin ER 5-30mg q.d + BT	[86]	0.92 (-0.31,2.12)	94 (58,97)	0.00

† median relative to a placebo intervention.
Rank denotes the posterior median estimate (95% credible interval). Ranks are calculated as the average treatment rank over all iterations and ranked according to the probability that each treatment is the best overall. p(Best) denotes the probability that the intervention in question is the best. The probability best is calculated based on the number of iterations for which the intervention is ranked in first place.

Table 2: Estimated posterior median difference (and 95% credible interval) in change from baseline for voiding episodes relative to placebo obtained from univariate network meta-analysis

**Treatment**	**Code**	**Median difference† (95% CrI)**	**Rank (95% CrI)**	**p(Best)**
Sacral nerve stimulation	[81]	-7.89 (-12.03,-3.76)	1 (1,2)	0.96
Electrostimulation + PFMT + BT	[97]	-3.37 (-5.44,-1.23)	3 (2,20)	0.02
Oxybutynin IR 2.5mg t.i.d	[21]	-3.12 (-4.25,-2.01)	4 (2,9)	0.01
OnaBoNT-A 200U trigone sparing	[73]	-2.93 (-4.23,-1.61)	4 (2,14)	0.00
PFMT + BT	[89]	-2.16 (-4.1,-0.22)	8 (3,71)	0.00
Estradiol 3mg intravaginally	[128]	-1.87 (-3.8,-0.01)	10 (2,80)	0.00
Tolterodine ER 4mg q.d + Neurostimulation	[96]	-1.93 (-2.7,-1.15)	10 (4,24)	0.00
OnaBoNT-A 100u bladder base + trigone	[79]	-1.79 (-3.45,-0.05)	11 (3,79)	0.00
Oxybutynin intravesically 5mg t.i.d	[14]	-1.73 (-4.63,1.12)	12 (2,95)	0.01
Cizolirtine citrate 400mg b.i.d	[57]	-1.6 (-2.97,-0.3)	14 (4,70)	0.00
Propiverine IR 30mg b.i.d	[117]	-1.54 (-3.78,0.64)	15 (2,91)	0.00
Estriol 1mg intravesically	[131]	-1.51 (-2.74,-0.27)	15 (5,71)	0.00
OnaBoNT-A 100U trigone sparing	[72]	-1.5 (-1.9,-1.11)	15 (9,26)	0.00
OnaBoNT-A 100u bladder body + trigone	[78]	-1.48 (-2.99,0)	16 (4,80)	0.00
Lipo-BoNTA 200U	[138]	-1.44 (-3.09,0.1)	17 (4,83)	0.00
Reflexology	[71]	-1.4 (-3.31,0.49)	17 (3,89)	0.00
Tolterodine IR 2mg b.i.d + BT	[93]	-1.41 (-2.45,-0.42)	17 (6,63)	0.00
Oxybutynin ER 10mg q.d	[8]	-1.33 (-1.99,-0.69)	19 (8,48)	0.00
Imidafenacin 0.25mg b.i.d	[37]	-1.25 (-2.06,-0.43)	21 (8,65)	0.00
Mirabegron IR 150mg b.i.d	[49]	-1.2 (-2.13,-0.29)	22 (8,71)	0.00
Solifenacin ER 10mg q.d	[30]	-1.16 (-1.41,-0.93)	23 (15,34)	0.00
Mirabegron IR 100mg b.i.d	[48]	-1.18 (-1.98,-0.39)	23 (9,67)	0.00
Electrostimulation	[80]	-1.15 (-1.86,-0.47)	24 (10,61)	0.00
Electrostimulation + vaginal oestrogen cream 1.25mg/day	[133]	-1.15 (-2.02,-0.31)	24 (9,69)	0.00
Pregabalin 150mg b.i.d + Tolterodine ER 4mg q.d	[102]	-1.11 (-1.81,-0.42)	25 (10,65)	0.00
Oxybutynin ER 2.5mg q.d + BT	[92]	-1.07 (-3.04,0.81)	26 (4,93)	0.00
Oxybutynin ER 2.5mg q.d	[20]	-1.05 (-2.67,0.58)	27 (6,91)	0.00
Oxybutynin vaginal ring 6mg q.d	[17]	-1 (-1.74,-0.26)	29 (11,72)	0.00
Fesoterodine ER 8mg q.d	[26]	-1.01 (-1.22,-0.8)	29 (19,42)	0.00
Solabegron IR 125mg b.i.d	[55]	-0.9 (-1.19,-0.61)	34 (20,55)	0.00
Mirabegron ER 50mg q.d	[51]	-0.8 (-1.01,-0.6)	39 (26,56)	0.00
Pregabalin 150mg b.i.d	[62]	-0.81 (-1.35,-0.27)	39 (17,72)	0.00
Tolterodine 2mg + Pilocarpine 9mg b.i.d	[101]	-0.8 (-1.29,-0.32)	39 (19,70)	0.00
Oxybutynin IR 3mg t.i.d	[19]	-0.8 (-1.33,-0.26)	40 (18,73)	0.00
Solifenacin ER 5-10mg q.d	[31]	-0.79 (-1.08,-0.51)	40 (24,62)	0.00
Trospium chloride ER 60mg q.d	[44]	-0.77 (-1.17,-0.38)	41 (22,68)	0.00
Mirabegron ER 25mg q.d	[50]	-0.76 (-1.1,-0.43)	42 (24,66)	0.00
Oxybutynin IR 2.5 - 5mg b.i.d	[24]	-0.73 (-1.39,-0.1)	44 (18,77)	0.00
Fesoterodine ER 4mg q.d	[25]	-0.73 (-0.92,-0.54)	44 (31,60)	0.00
Pelvic Floor Muscle Training (PFMT)/Physiotherapy	[84]	-0.71 (-2.14,0.77)	46 (9,93)	0.00
Oxybutynin chloride topical gel 1g/day	[13]	-0.7 (-1.21,-0.19)	47 (21,75)	0.00
Oxybutynin vaginal ring 4mg q.d	[16]	-0.7 (-1.4,0)	47 (17,81)	0.00
Fesoterodine ER 4 - 8mg q.d	[27]	-0.69 (-0.91,-0.48)	47 (31,64)	0.00
Solifenacin ER 5mg q.d	[29]	-0.69 (-0.92,-0.5)	47 (31,63)	0.00
Propiverine ER 20mg q.d	[41]	-0.69 (-0.92,-0.47)	47 (31,64)	0.00
Mirabegron ER 100mg q.d	[52]	-0.7 (-0.94,-0.46)	47 (30,65)	0.00
Oxybutynin gel 84mg/day	[134]	-0.7 (-1.35,-0.05)	47 (18,79)	0.00
Tolterodine IR 2mg b.i.d	[5]	-0.67 (-0.86,-0.5)	49 (35,62)	0.00
Tarafenacin 0.4mg q.d	[82]	-0.66 (-1.55,0.23)	50 (14,86)	0.00
Oxybutynin IR 5mg t.i.d	[7]	-0.65 (-1.09,-0.2)	51 (25,74)	0.00
Vaginal oestrogen cream 1.25mg/day	[132]	-0.65 (-1.5,0.17)	51 (16,86)	0.00
Tolterodine IR 1mg b.i.d	[6]	-0.63 (-1.11, -0.16)	52 (24,76)	0.00
Imidafenacin 0.05mg b.i.d	[35]	-0.62 (-1.26,0.02)	53 (20,82)	0.00
Tolterodine ER 4mg q.d	[4]	-0.62 (-0.74,-0.51)	53 (42,64)	0.00
Darifenacin ER 7.5mg q.d	[39]	-0.6 (-2.15,0.95)	54 (8,95)	0.00

Table 2: Estimated posterior median difference (and 95% credible interval) in change from baseline for voiding episodes relative to placebo obtained from univariate network meta-analysis (cont.)

Trospium chloride IR 45mg t.i.d	[47]	-0.6 (-1.35,0.12)	54 (19,84)	0.00
Oxybutynin trandermal 3.9mg/day	[10]	-0.57 (-0.96,-0.18)	56 (30,76)	0.00
Pregabalin 75mg b.i.d + Tolterodine ER 2mg q.d	[103]	-0.51 (-1.05,0.03)	60 (27,82)	0.00
Imidafenacin 0.1mg b.i.d	[36]	-0.5 (-0.82,-0.18)	61 (38,76)	0.00
Solabegron IR 50mg b.i.d	[54]	-0.5 (-0.79,-0.21)	61 (39,75)	0.00
Propiverine IR 45mg t.i.d	[118]	-0.46 (-2.59,1.61)	63 (6,96)	0.00
Bladder Training (BT)/Behaviour Therapy	[85]	-0.43 (-1.42,0.49)	64 (20,89)	0.00
Darifenacin ER 15mg q.d	[40]	-0.4 (-1.25,0.45)	65 (20,91)	0.00
Serlopitant 4mg q.d	[109]	-0.41 (-0.9,0.08)	65 (33,84)	0.00
Serlopitant 0.25mg q.d	[107]	-0.41 (-0.9,0.08)	65 (33,84)	0.00
Trospium chloride IR 15mg t.i.d + Physiotherapy	[91]	-0.4 (-3.71,2.64)	66 (3,98)	0.00
Terodiline 25mg b.i.d	[28]	-0.34 (-0.81,0.11)	68 (38,85)	0.00
Propiverine IR 15mg b.i.d	[43]	-0.35 (-1.23,0.56)	68 (22,91)	0.00
Netupitant 200mg q.d	[112]	-0.32 (-1.47,0.85)	69 (16,94)	0.00
Oxybutynin 20mg intravesically q.d	[106]	-0.31 (-1.64,1.02)	70 (13,95)	0.00
Elocalcitol 150mg	[69]	-0.29 (-1.07,0.49)	70 (26,91)	0.00
Oxybutynin gel 56mg/day	[135]	-0.3 (-0.98,0.37)	70 (30,90)	0.00
Control	[2]	-0.27 (-1.74,1.22)	71 (14,96)	0.00
Oxybutynin ER 5-30mg q.d + BT	[86]	-0.22 (-2.32,1.87)	73 (8,97)	0.00
Oxybutynin ER 5 - 30mg/day	[22]	-0.23 (-1.47,1.04)	73 (18,95)	0.00
Cizolirtine citrate 200mg b.i.d	[56]	-0.21 (-1.7,1.26)	73 (12,96)	0.00
Netupitant 100mg q.d	[111]	-0.19 (-1.27,0.93)	74 (20,95)	0.00
Tarafenacin 0.2mg q.d	[90]	-0.15 (-1.07,0.76)	75 (27,94)	0.00
Serlopitant 1mg q.d	[108]	-0.11 (-0.61,0.38)	77 (54,90)	0.00
Elocalcitol 75mg	[70]	-0.1 (-0.85,0.64)	77 (36,93)	0.00
Oxybutynin transdermal 1.3mg/day	[11]	-0.08 (-0.73,0.58)	78 (45,92)	0.00
Oxybutynin transdermal 2.6mg/day	[12]	-0.08 (-0.7,0.55)	78 (47,92)	0.00
Netupitant 50mg q.d	[110]	-0.09 (-1.19,0.99)	78 (23,95)	0.00
Percutaneous tibial nerve stimulation	[83]	-0.08 (-1.41,1.13)	78 (18,95)	0.00
Electromagnetic stimulation	[125]	-0.07 (-2.09,1.94)	78 (9,97)	0.00
ONO-8539 100mg b.i.d	[60]	-0.05 (-0.8,0.71)	79 (40,93)	0.00
Oxybutynin ER 15mg q.d	[9]	-0.04 (-1.22,1.16)	79 (21,96)	0.00
Placebo	[1]	NA	81 (73,87)	0.00
Resiniferatoxin 50nM	[67]	0.06 (-1.22,1.41)	82 (22,97)	0.00
Oxybutynin IR 5mg b.i.d	[18]	0.12 (-0.88,1.13)	84 (35,96)	0.00
ONO-8539 300mg b.i.d	[61]	0.17 (-0.59,0.93)	85 (55,95)	0.00
Propantheline Bromide 15mg t.i.d	[113]	0.33 (-0.84,1.45)	88 (38,97)	0.00
Propiverine ER 60mg q.d	[119]	0.45 (-1.78,2.69)	90 (13,98)	0.00
ONO-8539 30mg b.i.d	[59]	0.47 (-0.27,1.19)	90 (71,96)	0.00
Estradiol 1mg intravaginally	[127]	0.49 (-1.18,2.17)	91 (23,97)	0.00
ZD0947IL 25mg/day	[58]	0.71 (-0.58,2.03)	93 (56,97)	0.00
Sham therapy	[3]	0.94 (-0.53,2.35)	95 (61,98)	0.00
Naftopidil 25mg q.d	[114]	3.24 (0.7,5.57)	98 (93,100)	0.00
Trospium chloride IR 15mg t.i.d	[46]	4.59 (1.8,7.32)	99 (98,100)	0.00
Solifenacin ER 5mg q.d + Naftopidil 25mg q.d	[115]	5.19 (2.57,7.7)	100 (98,100)	0.00

† median relative to a placebo intervention.

Rank denotes the posterior median estimate (95% credible interval). Ranks are calculated as the average treatment rank over all iterations and ranked according to the probability that each treatment is the best overall. p(Best) denotes the probability that the intervention in question is the best. The probability best is calculated based on the number of iterations for which the intervention is ranked in first place.

Table 3: Estimated posterior median difference (and 95% credible interval) in change from baseline for urgency episodes relative to placebo obtained from univariate network meta-analysis

**Treatment**	**Code**	**Median difference† (95%CrI)**	**Rank (95%CrI)**	**p(Best)**
Electrostimulation + vaginal oestrogen cream 1.25mg/day	[133]	-6.94 (-8.65,-5.23)	1 (1,1)	0.99
Electrostimulation	[80]	-4.84 (-5.94,-3.74)	2 (2,4)	0.00
Vaginal oestrogen cream 1.25mg/day	[132]	-3.34 (-5.06,-1.62)	4 (3,15)	0.00
OnaBoNT-A 100u bladder body + trigone	[78]	-2.69 (-4.83,-0.54)	6 (2,36)	0.00
OnaBoNT-A 100u bladder base + trigone	[79]	-2.56 (-5.14,0.04)	7 (2,46)	0.00
Cizolirtine citrate 400mg b.i.d	[57]	-2.62 (-4.66,-0.59)	7 (2,35)	0.00
Percutaneous tibial nerve stimulation	[83]	-2.5 (-4.37,-0.64)	7 (3,34)	0.00
Tolterodine ER 4mg q.d + Neurostimulation	[96]	-2.38 (-3.82,-0.94)	8 (3,27)	0.00
OnaBoNT-A 200U trigone sparing	[73]	-2.25 (-3.98,-0.51)	9 (3,37)	0.00
OnaBoNT-A 100u trigone sparing	[72]	-2.07 (-3.06,-1.08)	10 (5,25)	0.00
Oxybutynin IR 2.5mg t.i.d	[21]	-1.94 (-3.02,-0.82)	11 (5,31)	0.00
Darifenacin ER 7.5mg q.d	[39]	-1.68 (-3.34,-0.02)	14 (4,45)	0.00
Imidafenacin 0.25mg b.i.d	[37]	-1.48 (-3.08,0.12)	16 (5,47)	0.00
Fesoterodine ER 8mg q.d	[26]	-1.47 (-2.03,-0.9)	17 (10,29)	0.00
Lipo-BoNTA 200U	[138]	-1.33 (-3.59,0.94)	19 (4,53)	0.00
Solifenacin ER 10mg q.d	[30]	-1.34 (-2.02,-0.69)	19 (10,33)	0.00
Fesoterodine ER 4 - 8mg q.d	[27]	-1.23 (-2.01,-0.46)	21 (10,39)	0.00
Mirabegron IR 150mg b.i.d	[49]	-1.15 (-2.8,0.5)	22 (6,51)	0.00
Mirabegron IR 100mg b.i.d	[48]	-1.14 (-2.64,0.37)	23 (7,50)	0.00
Solifenacin ER 5mg q.d	[29]	-1.07 (-1.7,-0.49)	24 (14,38)	0.00
Oxybutynin trandermal 3.9mg/day	[10]	-1.02 (-2.38,0.34)	25 (8,50)	0.00
Fesoterodine ER 4mg q.d	[25]	-1.05 (-1.55,-0.53)	25 (15,38)	0.00
Solifenacin ER 5 - 10mg q.d	[31]	-0.9 (-1.68,-0.13)	28 (14,44)	0.00
Propiverine ER 20mg q.d	[41]	-0.9 (-1.58,-0.23)	28 (15,43)	0.00
Propiverine IR 15mg b.i.d	[43]	-0.88 (-2.79,0.99)	29 (6,53)	0.00
Tolterodine IR 2mg b.i.d + BT	[93]	-0.77 (-2.42,0.85)	31 (8,51)	0.00
Pregabalin 150mg b.i.d	[62]	-0.78 (-2.02,0.45)	31 (11,50)	0.00
Tolterodine ER 4mg q.d	[4]	-0.77 (-1.16,-0.39)	31 (22,41)	0.00
Pregabalin 150mg b.i.d + Tolterodine ER 4mg q.d	[102]	-0.79 (-2.2,0.62)	31 (9,51)	0.00
Tarafenacin 0.4mg q.d	[82]	-0.74 (-2.53,1.02)	32 (7,53)	0.00
Elocalcitol 75mg	[70]	-0.7 (-2.17,0.77)	33 (9,52)	0.00
Imidafenacin 0.05mg b.i.d	[35]	-0.69 (-2.11,0.72)	33 (10,52)	0.00
Imidafenacin 0.1mg b.i.d	[36]	-0.72 (-1.58,0.13)	33 (15,48)	0.00
Mirabegron ER 100mg q.d	[52]	-0.69 (-1.4,0.01)	33 (18,46)	0.00
Cizolirtine citrate 200mg b.i.d	[56]	-0.65 (-2.48,1.16)	34 (8,53)	0.00
Mirabegron ER 50mg q.d	[51]	-0.67 (-1.24,-0.1)	34 (20,45)	0.00
Tolterodine IR 2mg b.i.d	[5]	-0.66 (-1.64,0.29)	34 (16,47)	0.00
Netupitant 100mg q.d	[111]	-0.54 (-1.93,0.86)	37 (11,52)	0.00
Elocalcitol 150mg	[69]	-0.4 (-1.86,1.07)	39 (12,53)	0.00
Mirabegron ER 25mg q.d	[50]	-0.41 (-1.37,0.54)	39 (18,51)	0.00
Oxybutynin ER 2.5mg q.d + BT	[92]	-0.38 (-3.06,2.27)	40 (5,54)	0.00
Tarafenacin 0.2mg q.d	[90]	-0.39 (-2.14,1.34)	40 (10,54)	0.00
Tolterodine IR 2mg b.i.d + PFMT	[95]	-0.36 (-2.08,1.33)	40 (11,54)	0.00
Darifenacin ER 15mg q.d.	[40]	-0.37 (-1.82,1.09)	40 (12,53)	0.00
Pregabalin 75mg b.i.d + Tolterodine ER 2mg q.d	[103]	-0.38 (-1.69,0.93)	40 (14,53)	0.00
Oxybutynin ER 2.5mg q.d	[20]	-0.19 (-2.82,2.46)	43 (6,54)	0.00
Bladder Training (BT)/Behaviour Therapy	[85]	-0.17 (-1.84,1.5)	43 (14,53)	0.00
ONO-8539 300mg b.i.d	[61]	-0.19 (-1.65,1.25)	43 (15,53)	0.00
Netupitant 50mg q.d	[110]	-0.17 (-1.55,1.22)	43 (16,54)	0.00
ZD0947IL 25mg/day	[58]	-0.09 (-2.04,1.87)	44 (11,54)	0.00
Netupitant 200mg q.d	[112]	-0.08 (-1.47,1.31)	45 (17,54)	0.00
Placebo	[1]	NA	46 (40,51)	0.00
ONO-8539 100mg b.i.d	[60]	0.06 (-1.39,1.51)	47 (18,54)	0.00
ONO-8539 30mg b.i.d	[59]	0.87 (-0.58,2.3)	53 (36,54)	0.00

† median relative to a placebo intervention.

Rank denotes the posterior median estimate (95% credible interval). Ranks are calculated as the average treatment rank over all iterations and ranked according to the probability that each treatment is the best overall. p(Best) denotes the probability that the intervention in question is the best. The probability best is calculated based on the number of iterations for which the intervention is ranked in first place.
