# Supplementary material for: Multivariate network meta-analysis incorporating class effects
Source: BMC Med Res Methodol. 2020 Jul 8;20:184. doi: 10.1186/s12874-020-01025-8 (PMC7341581; doi:10.1186/s12874-020-01025-8)
Supplement: Supplementary file 6 — Additional file 6 Treatment profiles obtained from univariate network meta-analyses for change from baseline in incontinence, voiding and urgency episodes. [file 12874_2020_1025_MOESM6_ESM.pdf]

Additional file 6 — Treatment profiles obtained from univariate network meta-analyses for change from baseline in incontinence, voiding and urgency episodes

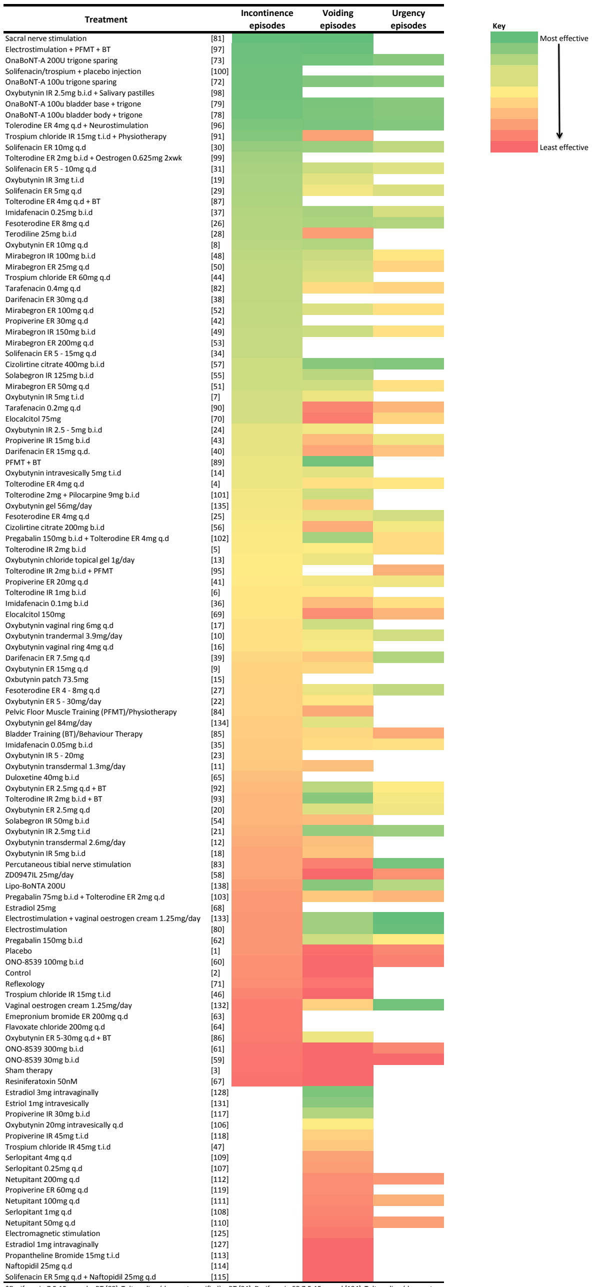

*Darifenacin 7.5-15mg q.d + BT [88], Tolterodine (dose not specified) + BT [94], Darifenacin ER 7.5-15mg q.d [104], Tolterodine (dose not Sacral nerve stimulation + Tolterodine ER 2mg q.d [136], Tolterodine ER 2mg q.d [137] were disconnected from the network
