# Supplementary material for: Multivariate network meta-analysis incorporating class effects
Source: BMC Med Res Methodol. 2020 Jul 8;20:184. doi: 10.1186/s12874-020-01025-8 (PMC7341581; doi:10.1186/s12874-020-01025-8)

Additional file 7 — Convergence diagnostics

Figure 1: Brooks-Gelman-Rubin plots obtained from multivariate hierarchical network meta-analysis evaluating the mean change from baseline in urinary incontinence, voiding and urgency episodes

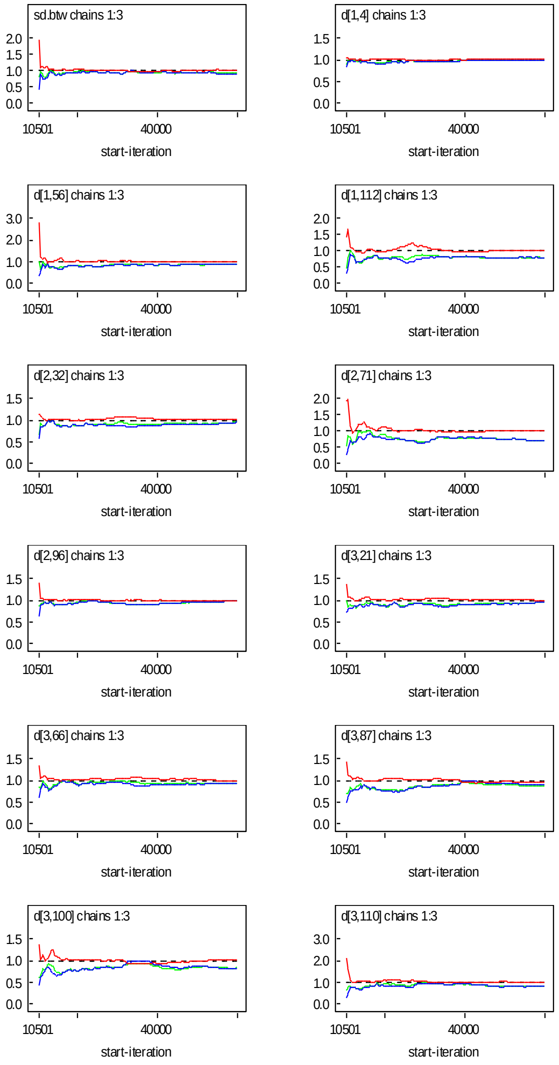

Figure 2: Autocorrelation plots obtained from multivariate hierarchical network meta-analysis evaluating the mean change from baseline in urinary incontinence, voiding and urgency episodes

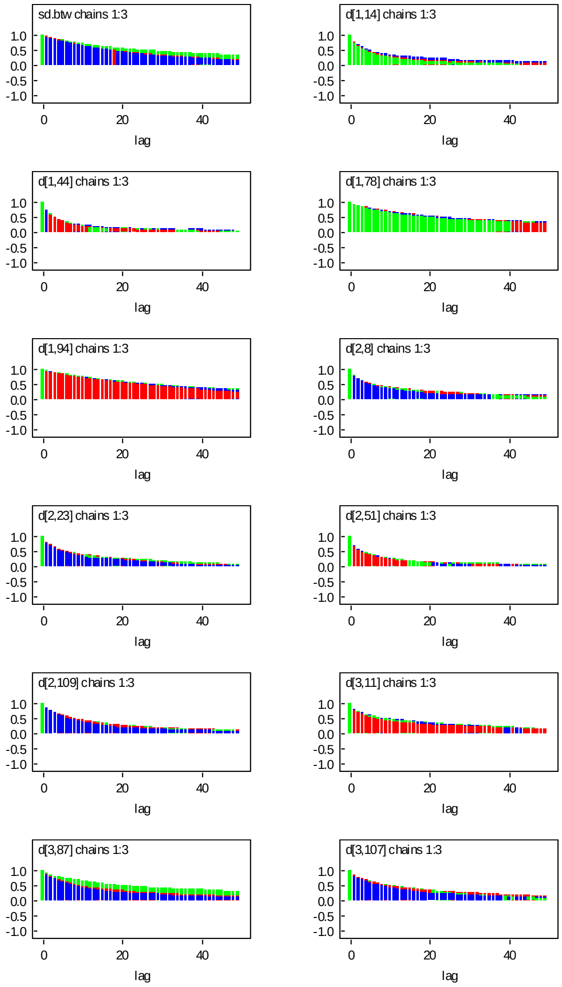

Figure 3: History and trace plots obtained from multivariate hierarchical network meta-analysis evaluating the mean change from baseline in urinary incontinence, voiding and urgency episodes

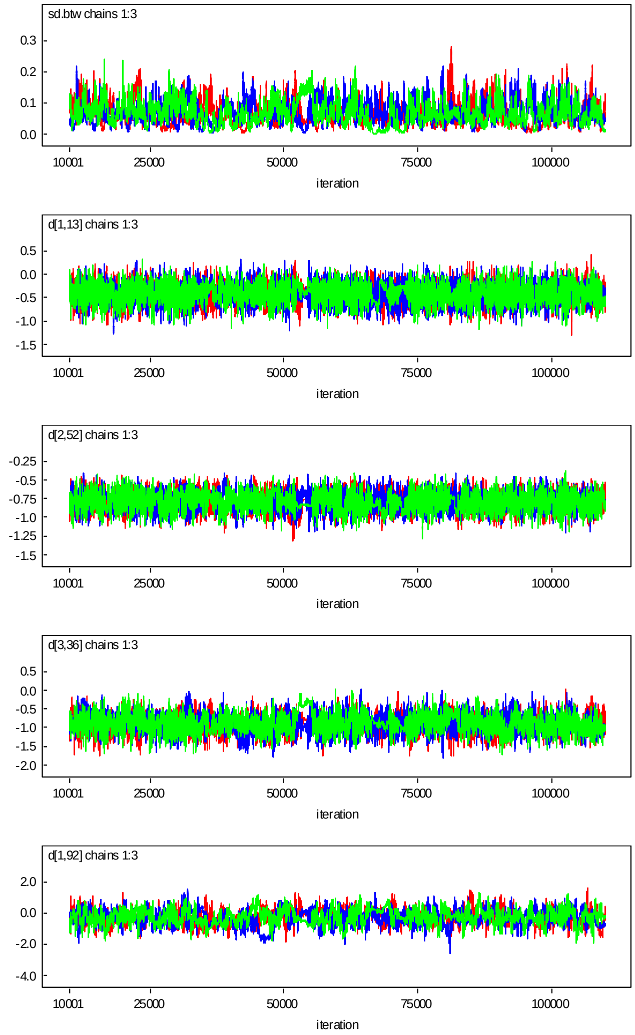

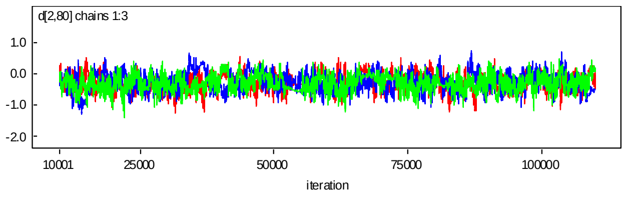
d[2,80] chains 1:3
1.0
0.0
-1.0
-2.0
10001
25000
50000
75000
100000
iteration

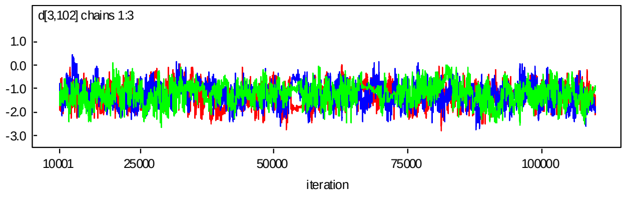
d[3,102] chains 1:3
1.0
0.0
-1.0
-2.0
-3.0
10001
25000
50000
75000
100000
iteration

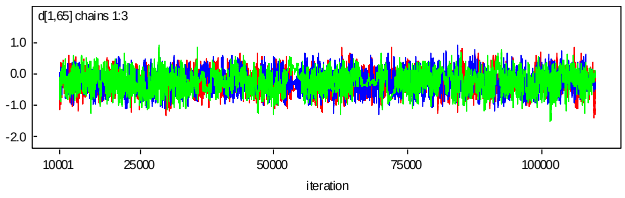
d[1,65] chains 1:3
1.0
0.0
-1.0
-2.0
10001
25000
50000
75000
100000
iteration

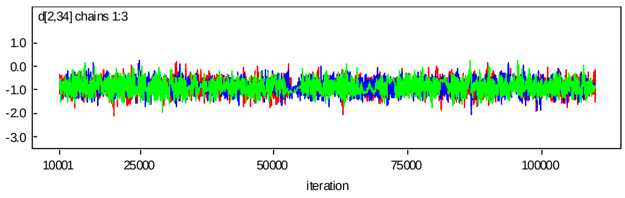
d[2,34] chains 1:3
1.0
0.0
-1.0
-2.0
-3.0
10001
25000
50000
75000
100000
iteration

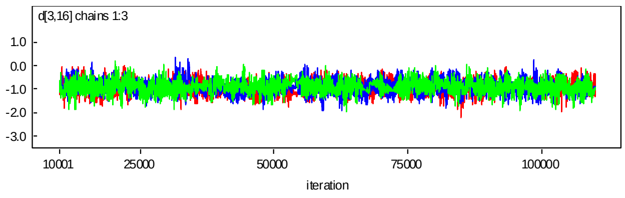
d[3,16] chains 1:3
1.0
0.0
-1.0
-2.0
-3.0
10001
25000
50000
75000
100000
iteration

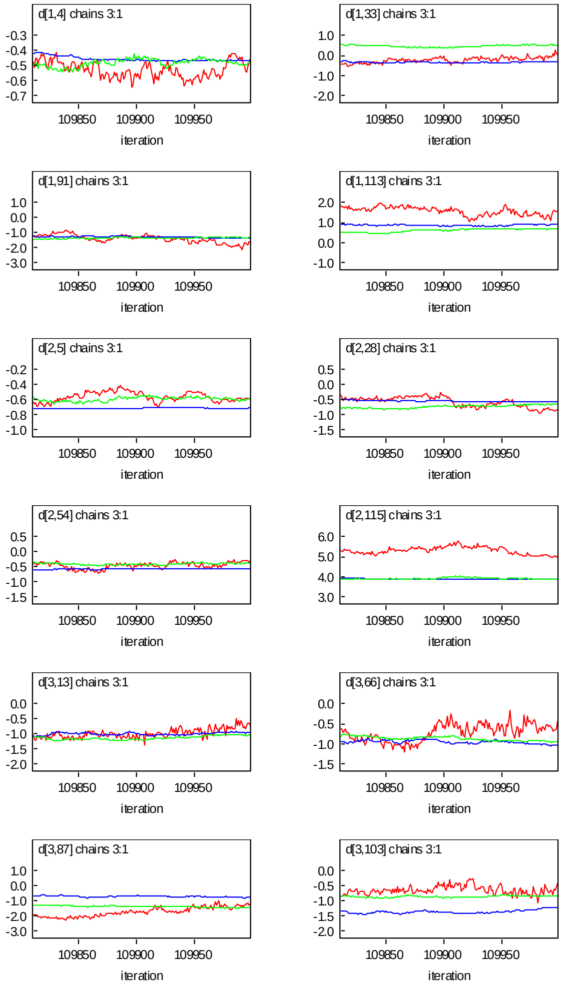
d[1,4] chains 3:1
d[1,33] chains 3:1
d[1,91] chains 3:1
d[1,113] chains 3:1
d[2,5] chains 3:1
d[2,28] chains 3:1
d[2,54] chains 3:1
d[2,115] chains 3:1
d[3,13] chains 3:1
d[3,66] chains 3:1
d[3,87] chains 3:1
d[3,103] chains 3:1
109850
109900
109950
iteration

Figure 4: Density plots obtained from multivariate hierarchical network meta-analysis evaluating the mean change from baseline in urinary incontinence, voiding and urgency episodes

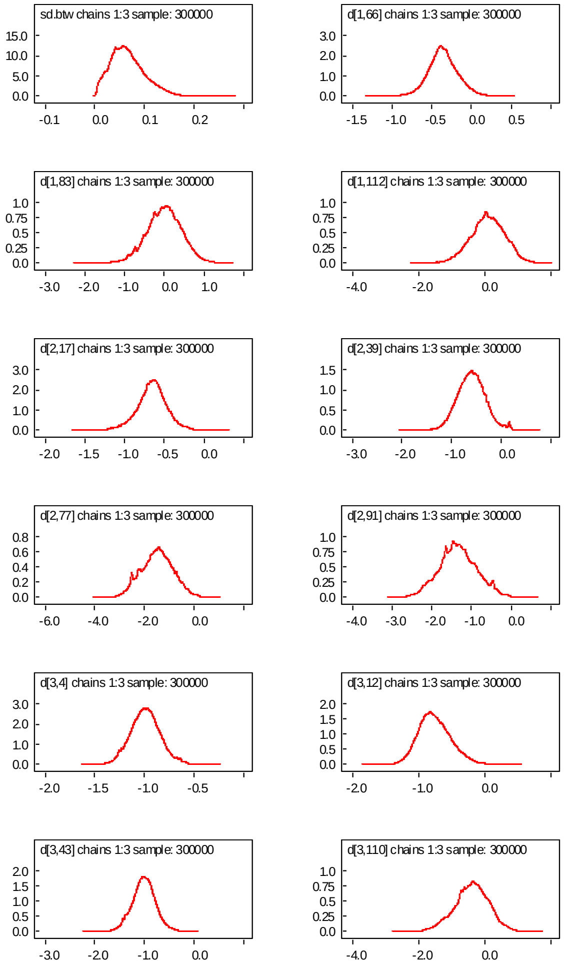

Supplement: Supplementary file 7 — Additional file 7 Convergence diagnostics. [file 12874_2020_1025_MOESM7_ESM.pdf]
