# Supplementary material for: Multivariate network meta-analysis incorporating class effects
Source: BMC Med Res Methodol. 2020 Jul 8;20:184. doi: 10.1186/s12874-020-01025-8 (PMC7341581; doi:10.1186/s12874-020-01025-8)
Supplement: Supplementary file 8 — Additional file 8 Sensitivity analyses. [file 12874_2020_1025_MOESM8_ESM.pdf]

Additional file 8 — Sensitivity analyses

Table 1: Sensitivity analyses assessing the impact in change from baseline in incontinence episodes for different choices of prior distribution on variance parameters for multivariate hierarchical network meta-analysis

**Treatment**	**Code**	**Median difference† (95%CrI)**	**Median difference†† (95%CrI)**	**Median difference§ (95%CrI)**	**Median difference§§ (95%CrI)**
Sacral nerve stimulation	[81]	-8.58 (-10.95,-6.13)	-7.87 (-9.75,-6.08)	-8.53 (-10.46,-5.77)	-7.95 (-10.38,-5.62)
OnaBoNT-A 200u trigone sparing	[73]	-2.09 (-2.98,-1.42)	-2 (-2.95,-1.39)	-1.98 (-3.09,-1.39)	-2.03 (-2.96,-1.42)
Oxybutynin IR 2.5mg b.i.d + Salivary pastilles	[98]	-1.87 (-3.9,0.08)	-2.08 (-3.69,-0.06)	-1.99 (-3.6,-0.26)	-1.65 (-3.37,0.36)
Electrostimulation + PFE + Bladder training	[97]	-1.99 (-2.8,-1.06)	-1.82 (-2.79,-0.94)	-1.93 (-2.86,-0.67)	-1.86 (-2.83,-0.93)
Solifenacin/trospium + placebo injection	[100]	-1.78 (-2.86,-0.87)	-1.67 (-2.63,-0.78)	-1.7 (-2.68,-0.75)	-1.71 (-2.66,-0.86)
OnaBoNT-A 100u bladder base + trigone	[79]	-1.74 (-3.11,-0.17)	-1.67 (-3.27,-0.47)	-1.74 (-2.82,-0.79)	-1.77 (-2.88,-0.96)
OnaBoNT-A 100u bladder body + trigone	[78]	-1.65 (-2.44,-0.84)	-1.54 (-2.42,-0.87)	-1.65 (-2.33,-0.82)	-1.64 (-2.35,-0.85)
OnaBoNTA 100u trigone sparing	[72]	-1.69 (-2.17,-1.31)	-1.58 (-2.06,-1.28)	-1.62 (-2.05,-1.25)	-1.64 (-2.03,-1.29)
Tolerodine ER 4mg q.d + Neurostimulation	[96]	-1.33 (-1.75,-0.92)	-1.32 (-1.74,-0.95)	-1.36 (-1.77,-0.94)	-1.33 (-1.73,-0.93)
Estriol 1mg intravesival	[131]	-1.21 (-4.51,16.41)	-1.51 (-12.74,-0.35)	-1.12 (-2.41,-0.06)	-1.24 (-2.52,-0.02)
Trospium IR 15mg t.i.d + Physiotherapy	[91]	-1.07 (-1.95,-0.07)	-0.94 (-1.98,-0.04)	-1.02 (-1.86,-0.12)	-1.07 (-1.92,-0.25)
Estradiol 3mg intravaginally	[128]	-1.25 (-17.22,0.4)	-0.87 (-3.19,1.97)	-0.84 (-2.65,0.65)	-1.13 (-2.95,0.22)
Solifenacin ER 10mg q.d	[30]	-0.84 (-1.07,-0.62)	-0.86 (-1.06,-0.63)	-0.85 (-1.07,-0.63)	-0.84 (-1.09,-0.62)
Tolterodine ER 2mg b.i.d + Oestrogen 0.625mg 2xwk	[99]	-0.74 (-1.24,-0.24)	-0.78 (-1.26,-0.26)	-0.7 (-1.21,-0.22)	-0.74 (-1.24,-0.22)
Tolterodine ER 4mg q.d + Behaviour therapy	[87]	-0.69 (-1.35,-0.1)	-0.67 (-1.34,-0.01)	-0.7 (-1.35,-0.15)	-0.71 (-1.32,-0.1)
Pregabalin 150mg b.i.d + Tolterodine ER 4mg q.d	[102]	-0.68 (-1.46,0.09)	-0.84 (-1.46,-0.02)	-0.73 (-1.44,-0.11)	-0.73 (-1.44,-0.09)
Fesoterodine ER 8mg q.d	[26]	-0.69 (-0.88,-0.51)	-0.69 (-0.88,-0.51)	-0.7 (-0.89,-0.52)	-0.69 (-0.88,-0.5)
Imidafenacin IR 0.25mg b.i.d	[37]	-0.76 (-1.34,-0.22)	-0.76 (-1.29,-0.21)	-0.75 (-1.27,-0.26)	-0.75 (-1.34,-0.23)
Solifenacin ER (5mg-10mg) q.d	[31]	-0.68 (-0.92,-0.44)	-0.67 (-0.91,-0.45)	-0.68 (-0.9,-0.45)	-0.67 (-0.89,-0.45)
Solifenacin ER 5mg - 15mg q.d	[34]	-0.65 (-1.05,-0.23)	-0.65 (-1.06,-0.22)	-0.66 (-1.03,-0.26)	-0.65 (-1.01,-0.23)
Mirabegron 100mg b.i.d	[48]	-0.68 (-1.55,-0.37)	-0.66 (-1.34,-0.39)	-0.65 (-1.04,-0.39)	-0.65 (-1.09,-0.39)
Solabegron 125mg b.i.d	[55]	-0.63 (-0.92,-0.32)	-0.63 (-0.9,-0.34)	-0.63 (-0.9,-0.34)	-0.63 (-0.91,-0.34)
Propiverine ER 30mg q.d	[42]	-0.62 (-1.15,-0.2)	-0.64 (-1.21,-0.21)	-0.57 (-1.08,-0.25)	-0.58 (-1.14,-0.22)
Darifenacin ER 30mg q.d	[38]	-0.64 (-1.25,-0.07)	-0.65 (-1.22,-0.15)	-0.61 (-1.2,-0.11)	-0.66 (-1.28,-0.13)
Mirabegron 25mg q.d	[50]	-0.63 (-0.9,-0.4)	-0.62 (-0.86,-0.41)	-0.63 (-0.86,-0.41)	-0.62 (-0.86,-0.41)
Mirabegron 150mg b.i.d	[49]	-0.6 (-1.16,0.08)	-0.62 (-1.05,-0.11)	-0.61 (-0.97,-0.31)	-0.61 (-1.02,-0.31)
Mirabegron 100mg q.d	[52]	-0.61 (-0.79,-0.43)	-0.6 (-0.78,-0.43)	-0.6 (-0.78,-0.43)	-0.6 (-0.78,-0.43)
Mirabegron 200mg q.d	[53]	-0.6 (-0.97,-0.19)	-0.61 (-0.92,-0.27)	-0.61 (-0.9,-0.3)	-0.6 (-0.89,-0.31)
Oxybutynin ER 10mg q.d	[8]	-0.68 (-1.33,-0.27)	-0.64 (-1.22,-0.28)	-0.61 (-1.13,-0.26)	-0.63 (-1.07,-0.27)
Propiverine 30mg b.i.d	[42]	-0.69 (-20.04,0.22)	-0.81 (-14.1,-0.16)	-0.58 (-1.76,-0.09)	-0.64 (-2.03,-0.1)
Oxybutynin IR 3mg t.i.d	[19]	-0.66 (-1.14,-0.32)	-0.61 (-1.06,-0.33)	-0.6 (-0.96,-0.31)	-0.62 (-1,-0.32)
Mirabegron 50mg q.d	[51]	-0.58 (-0.74,-0.41)	-0.58 (-0.74,-0.42)	-0.58 (-0.75,-0.42)	-0.58 (-0.75,-0.42)
Solifenacin ER 5mg q.d	[29]	-0.6 (-0.82,-0.39)	-0.59 (-0.8,-0.4)	-0.58 (-0.79,-0.41)	-0.6 (-0.79,-0.39)
Tolterodine IR 2mg b.i.d + BT	[93]	-0.47 (-1.06,0.25)	-0.51 (-1.1,0.19)	-0.51 (-1.01,-0.03)	-0.54 (-1.03,-0.04)
Cizolirtine Citrate 400mg b.i.d	[57]	-0.54 (-1.1,-0.01)	-0.6 (-1.08,-0.08)	-0.5 (-1.06,0)	-0.54 (-1.04,0.01)
Trospium ER 60mg q.d	[44]	-0.58 (-0.97,-0.19)	-0.55 (-0.93,-0.22)	-0.56 (-0.92,-0.2)	-0.56 (-0.91,-0.21)
Propiverine 45mg t.i.d	[118]	-0.44 (-4.47,7.68)	-0.43 (-1.67,7.71)	-0.47 (-1.27,0.21)	-0.49 (-1.45,0.41)
Tolterodine IR 2mg b.i.d + Pilocarpine 9mg b.i.d	[101]	-0.49 (-0.8,-0.18)	-0.49 (-0.8,-0.2)	-0.51 (-0.79,-0.21)	-0.5 (-0.81,-0.21)
Fesoterodine ER 4mg q.d	[25]	-0.5 (-0.66,-0.33)	-0.5 (-0.67,-0.34)	-0.5 (-0.66,-0.34)	-0.5 (-0.66,-0.33)
Pregabalin 150mg b.i.d	[62]	-0.43 (-0.97,0.53)	-0.48 (-1.02,0.45)	-0.47 (-1.06,0.05)	-0.49 (-1.08,0.1)
Darifenacin ER 15mg q.d	[40]	-0.49 (-0.91,-0.03)	-0.5 (-0.91,-0.11)	-0.5 (-0.91,-0.08)	-0.5 (-0.93,-0.08)
Propiverine IR 15mg b.i.d	[43]	-0.48 (-0.87,-0.12)	-0.52 (-0.86,-0.19)	-0.48 (-0.83,-0.15)	-0.48 (-0.85,-0.14)
Tolterodine ER 4mg q.d	[4]	-0.49 (-0.6,-0.39)	-0.49 (-0.59,-0.38)	-0.49 (-0.59,-0.39)	-0.49 (-0.59,-0.39)
Oxybutynin IR 5mg t.i.d	[7]	-0.48 (-0.87,-0.19)	-0.5 (-0.82,-0.22)	-0.46 (-0.75,-0.19)	-0.47 (-0.75,-0.2)
Propiverine ER 20mg q.d	[41]	-0.42 (-0.61,-0.23)	-0.44 (-0.62,-0.24)	-0.43 (-0.61,-0.25)	-0.43 (-0.61,-0.25)
Tolterodine IR 2mg b.i.d	[5]	-0.44 (-0.57,-0.31)	-0.43 (-0.57,-0.31)	-0.44 (-0.56,-0.31)	-0.44 (-0.57,-0.31)
Propiverine 60mg q.d	[119]	-0.24 (-0.91,48.73)	-0.41 (-1.07,25.03)	-0.41 (-1.07,0.93)	-0.39 (-0.99,0.75)
Oxybutynin intravesically 5mg t.i.d	[14]	-0.49 (-1.85,-0.03)	-0.44 (-1.61,0)	-0.45 (-1.04,-0.01)	-0.44 (-0.99,-0.03)
Oxybutynin IR (2.5-5mg) b.i.d	[24]	-0.47 (-1.3,-0.05)	-0.42 (-1.01,-0.08)	-0.44 (-0.86,-0.05)	-0.43 (-0.79,-0.09)
Oxybutynin chloride topical gel 1g q.d	[13]	-0.43 (-0.86,-0.08)	-0.43 (-0.79,-0.11)	-0.41 (-0.73,-0.1)	-0.41 (-0.75,-0.11)
Oxybutynin vaginal ring 6mg q.d	[17]	-0.41 (-0.88,0.01)	-0.42 (-0.81,-0.03)	-0.42 (-0.83,-0.07)	-0.41 (-0.78,-0.06)
Tolterodine IR 2mg b.i.d + PFE	[95]	-0.43 (-1.01,0.18)	-0.4 (-1.13,0.18)	-0.4 (-1.03,0.29)	-0.44 (-1.04,0.23)
PFMT + BT	[89]	-0.41 (-0.91,0.21)	-0.37 (-0.9,0.31)	-0.38 (-0.94,0.24)	-0.38 (-0.92,0.16)
Tolterodine IR 1mg b.i.d	[6]	-0.39 (-0.65,-0.08)	-0.39 (-0.62,-0.1)	-0.41 (-0.63,-0.07)	-0.4 (-0.63,-0.11)
Fesoterodine ER (4mg-8mg) q.d	[27]	-0.37 (-0.59,-0.13)	-0.4 (-0.59,-0.14)	-0.39 (-0.59,-0.18)	-0.4 (-0.59,-0.17)
Oxybutynin gel 84mg/day	[134]	-0.37 (-0.85,0.19)	-0.34 (-0.79,0.06)	-0.38 (-0.73,-0.01)	-0.38 (-0.71,0)
Oxybutynin transdermal 3.9mg/day	[10]	-0.38 (-0.65,-0.09)	-0.37 (-0.63,-0.12)	-0.39 (-0.63,-0.14)	-0.39 (-0.64,-0.14)

Table 1: Sensitivity analyses assessing the impact in change from baseline in incontinence episodes for different choices of prior distribution on variance parameters for multivariate hierarchical network meta-analysis (cont.)

Oxybutynin vaginal ring 4mg q.d	[16]	-0.38 (-0.86,0.05)	-0.36 (-0.79,0.03)	-0.38 (-0.74,-0.03)	-0.37 (-0.72,0.01)
Imidafenacin IR 0.1mg b.i.d	[36]	-0.38 (-0.64,-0.12)	-0.37 (-0.64,-0.1)	-0.37 (-0.63,-0.12)	-0.38 (-0.63,-0.13)
Terodiline IR 25mg b.i.d	[28]	-0.41 (-1,0.06)	-0.36 (-1.01,0.07)	-0.37 (-0.77,0.04)	-0.37 (-0.81,0.08)
Darifenacin ER 7.5mg q.d	[39]	-0.34 (-0.85,0.19)	-0.39 (-0.87,0.15)	-0.36 (-0.81,0.22)	-0.39 (-0.89,0.1)
Oxybutynin gel 56mg/day	[135]	-0.4 (-1.17,0.07)	-0.37 (-1.12,0.06)	-0.36 (-0.74,0.02)	-0.36 (-0.73,0.05)
Oxbutynin patch 73.5mg	[15]	-0.33 (-0.62,-0.02)	-0.35 (-0.6,-0.05)	-0.34 (-0.61,-0.03)	-0.34 (-0.61,-0.06)
Elocalcitol 75mg	[70]	-0.38 (-1,0.2)	-0.42 (-0.94,0.14)	-0.31 (-0.88,0.21)	-0.37 (-0.91,0.16)
Oxybutynin 20mg intravesically q.d	[106]	-0.38 (-20.48,6.04)	-0.32 (-2.66,5.3)	-0.33 (-0.75,0.14)	-0.36 (-0.89,0.14)
Imidafenacin IR 0.05mg b.i.d	[35]	-0.32 (-0.8,0.16)	-0.29 (-0.71,0.12)	-0.33 (-0.73,0.12)	-0.34 (-0.77,0.09)
Oxybutynin ER 15mg q.d	[9]	-0.3 (-0.82,0.47)	-0.28 (-0.76,0.32)	-0.31 (-0.69,0.19)	-0.29 (-0.67,0.18)
Oxybutynin IR (5-20mg)	[23]	-0.27 (-0.71,1.34)	-0.28 (-0.7,1.3)	-0.32 (-0.71,0.28)	-0.31 (-0.72,0.25)
Oxybutynin ER (5-30mg) q.d	[22]	-0.27 (-0.62,0.56)	-0.26 (-0.61,0.61)	-0.29 (-0.63,0.16)	-0.31 (-0.63,0.13)
Trospium chloride 45mg t.i.d	[47]	-0.22 (-0.81,64.6)	-0.23 (-0.74,39.26)	-0.3 (-0.81,0.3)	-0.3 (-0.81,0.25)
Cizolirtine citrate 200mg b.i.d	[56]	-0.32 (-1.37,0.84)	-0.39 (-1.27,1.04)	-0.25 (-1.28,0.93)	-0.24 (-1.17,0.83)
PFMT	[84]	-0.27 (-0.83,0.41)	-0.18 (-0.82,0.39)	-0.25 (-0.83,0.33)	-0.28 (-0.84,0.29)
Oxybutynin transdermal 1.3mg/day	[11]	-0.25 (-0.66,0.35)	-0.28 (-0.6,0.28)	-0.25 (-0.58,0.19)	-0.27 (-0.6,0.16)
Elocalcitol 150mg	[69]	-0.29 (-0.89,0.32)	-0.32 (-0.85,0.2)	-0.24 (-0.8,0.28)	-0.32 (-0.87,0.22)
Oxybutynin ER 2.5mg q.d	[20]	-0.2 (-0.61,1.17)	-0.24 (-0.69,1.04)	-0.27 (-0.65,0.28)	-0.25 (-0.61,0.3)
Duloxetine IR 40mg b.i.d	[65]	-0.26 (-0.78,0.24)	-0.27 (-0.77,0.21)	-0.24 (-0.8,0.29)	-0.24 (-0.77,0.26)
Bladder Training	[85]	-0.23 (-0.63,0.45)	-0.21 (-0.64,0.47)	-0.24 (-0.62,0.17)	-0.25 (-0.63,0.16)
Solabegron 50mg b.i.d	[54]	-0.24 (-0.55,0.06)	-0.27 (-0.52,0.04)	-0.26 (-0.55,0.03)	-0.25 (-0.53,0.03)
Oxybutynin IR 2.5mg t.i.d	[21]	-0.17 (-0.47,0.19)	-0.2 (-0.47,0.14)	-0.21 (-0.49,0.09)	-0.21 (-0.48,0.14)
Oxybutynin transdermal 2.6mg/day	[12]	-0.14 (-0.52,0.64)	-0.17 (-0.52,0.53)	-0.19 (-0.52,0.33)	-0.19 (-0.52,0.31)
Pregabalin 75mg b.i.d + Tolterodine ER 2mg q.d	[103]	-0.16 (-0.67,0.35)	-0.18 (-0.67,0.31)	-0.22 (-0.68,0.22)	-0.22 (-0.71,0.31)
Oxybutynin ER 2.5mg q.d + Bladder training	[92]	-0.14 (-1.02,0.94)	-0.21 (-1.03,0.97)	-0.29 (-1.4,0.55)	-0.16 (-1.05,0.81)
Oxybutynin IR 5mg b.i.d	[18]	-0.11 (-0.5,0.7)	-0.12 (-0.56,0.59)	-0.16 (-0.51,0.39)	-0.15 (-0.52,0.42)
Lipo-BoNTA	[138]	-0.15 (-1.02,0.75)	-0.12 (-1.05,0.84)	-0.15 (-0.98,0.61)	-0.18 (-1.02,0.75)
Serlopitant 0.25mg q.d	[107]	-0.05 (-0.58,31.41)	-0.19 (-1.84,2.8)	-0.09 (-0.62,0.35)	-0.14 (-0.7,0.35)
Serlopitant 4mg q.d	[109]	-0.11 (-4.43,15.12)	-0.19 (-13.84,0.31)	-0.1 (-0.67,0.34)	-0.14 (-0.69,0.35)
Tarafenacin 0.4mg q.d	[82]	-0.19 (-0.84,0.44)	-0.23 (-1.1,0.4)	-0.2 (-0.81,0.4)	-0.14 (-0.75,0.43)
Electrostimulation + vaginal oestrogen cream 1.25mg/day	[133]	-0.02 (-0.7,0.67)	-0.05 (-0.69,0.61)	-0.1 (-0.76,0.59)	-0.1 (-0.78,0.67)
Electrostimulation	[80]	0 (-0.48,0.51)	-0.05 (-0.51,0.44)	-0.08 (-0.52,0.39)	-0.08 (-0.53,0.43)
Serlopitant 1mg q.d	[108]	0.08 (-0.51,24.38)	0 (-11.79,5.32)	0.05 (-0.45,0.63)	0.01 (-0.5,0.56)
Estradiol 25mg	[68]	0 (-0.39,0.4)	0.01 (-0.38,0.37)	-0.01 (-0.41,0.4)	-0.01 (-0.4,0.38)
Placebo	[1]	NA	NA	NA	NA
Netupitant 200mg q.d	[112]	-0.09 (-21.62,1.02)	0.08 (-46.75,1.27)	0.04 (-0.91,1.13)	-0.06 (-1.13,0.97)
Netupitant 100mg q.d	[111]	0.17 (-13.54,7.35)	0.23 (-1.46,3.13)	0.11 (-0.89,1.06)	-0.06 (-1.13,1.02)
Tarafenacin 0.2mg q.d	[90]	0.05 (-0.57,0.72)	0.02 (-0.56,0.7)	-0.01 (-0.62,0.64)	0.09 (-0.5,0.68)
Trospium IR 15mg t.i.d	[46]	0.09 (-0.69,1.15)	0.1 (-0.64,1.13)	0.1 (-0.61,1.11)	0.02 (-0.65,0.88)
ZD0947IL 25mg/day	[58]	0.06 (-0.79,0.95)	0.05 (-0.81,1.1)	0.11 (-0.69,0.98)	0.14 (-0.65,1.11)
Netupitant 50mg q.d	[110]	0.26 (-6.15,14.21)	0.1 (-25.33,1.2)	0.11 (-0.89,1.27)	0.04 (-0.95,1.11)
Electromagnetic stimulation	[125]	-0.02 (-2.12,29.45)	0.14 (-1.99,48.32)	-0.28 (-1.82,1.51)	-0.39 (-1.87,1.47)
Oxybutynin ER 5-30mg/day + Behaviour therapy	[22]	0.36 (-0.51,1.7)	0.47 (-0.57,1.8)	0.37 (-0.62,1.25)	0.28 (-0.59,1.31)
ONO-8539 100mg b.i.d	[60]	0.13 (-0.65,0.73)	0.12 (-0.56,0.77)	0.11 (-0.6,0.79)	0.13 (-0.51,0.72)
Percutaneous tibial nerve stimulation	[83]	-0.03 (-1.07,0.91)	0.06 (-1.04,0.84)	0.02 (-0.86,0.92)	0.06 (-0.88,0.93)
Vaginal oestrogen cream 1.25mg/day	[132]	0.31 (-0.23,0.9)	0.24 (-0.3,0.83)	0.23 (-0.28,0.75)	0.25 (-0.33,0.79)
Flavoxate chloride 200mg q.d	[64]	0.35 (-0.32,1.02)	0.36 (-0.35,1.12)	0.36 (-0.32,1.06)	0.36 (-0.34,1.02)
Resiniferatoxin 50nM	[67]	0.38 (-0.82,2.02)	0.43 (-1.08,1.46)	0.43 (-0.63,1.61)	0.37 (-0.64,1.67)
Emepronium bromide 200mg q.d	[63]	0.38 (-0.26,1.02)	0.39 (-0.34,1.03)	0.36 (-0.31,1.2)	0.35 (-0.34,1.06)
ONO-8539 300mg b.i.d	[61]	0.48 (-0.15,1.08)	0.48 (-0.16,1.01)	0.42 (-0.16,0.99)	0.43 (-0.15,1)
Propantheline Bromide 15mg t.i.d	[113]	0.38 (-9.42,1.6)	0.34 (-31.19,1.48)	0.62 (-0.53,1.92)	0.49 (-0.61,1.63)
Estradiol 1mg intravaginally	[127]	0.46 (-52.47,2.32)	0.42 (-18.4,2.01)	0.6 (-0.76,1.91)	0.51 (-0.89,1.9)
ONO-8539 30mg b.i.d	[59]	0.51 (-0.12,1.14)	0.46 (-0.1,1.07)	0.5 (-0.13,1.08)	0.5 (-0.11,1.11)
Control	[2]	0.52 (-0.22,1.44)	0.68 (-0.11,1.47)	0.58 (-0.16,1.29)	0.59 (-0.28,1.3)
Reflexology	[71]	0.54 (-0.31,1.53)	0.72 (-0.17,1.62)	0.62 (-0.22,1.39)	0.62 (-0.3,1.41)
Sham Therapy	[3]	0.59 (-0.59,1.6)	0.66 (-0.51,1.54)	0.68 (-0.31,1.64)	0.71 (-0.28,1.64)
Naftopidil 25mg q.d	[114]	3.38 (-43.89,6.7)	3.95 (0.79,12.92)	3.55 (1.11,6.03)	3.86 (2.21,5.77)
Solifenacin succinate 5mg q.d + Naftopidil 25mg q.d	[115]	6.41 (0.96,25.19)	6.08 (3.56,28.61)	5.15 (2.89,7.53)	5.95 (4.1,8.26)

† Elements of $\boldsymbol{V^{1/2}}$ based on a Gamma(0.001,0.001) prior distribution on the precision scale

†† Elements of $\boldsymbol{V^{1/2}}$ based on a Half-Normal(0,1)I(0,) prior distribution on the standard deviation scale

§ Deviance of treatment effect profiles across outcomes, τ, based on a Gamma(0.01,0.01) prior distribution on the precision scale

§§ Deviance of treatment effect profiles across outcomes, τ, based on a Half-Normal(0,1)I(0,) prior distribution on the standard deviation scale

Table 2: Sensitivity analyses assessing the impact in change from baseline in voiding episodes for different choices of prior distribution on variance parameters for multivariate hierarchical network meta-analysis

**Treatment**	**Code**	**Median difference† (95%CrI)**	**Median difference†† (95%CrI)**	**Median difference§ (95%CrI)**	**Median difference§§ (95%CrI)**
Sacral nerve stimulation	[81]	-8.62 (-11.32,-3.88)	-8.07 (-9.87,-5.77)	-8.75 (-10.67,-5.98)	-8.16 (-10.62,-5.81)
OnaBoNT-A 200u trigone sparing	[73]	-2.34 (-3.74,-1.63)	-2.22 (-3.6,-1.6)	-2.19 (-3.32,-1.61)	-2.25 (-3.18,-1.61)
Oxybutynin IR 2.5mg b.i.d + Salivary pastilles	[98]	-2.06 (-10.46,2.26)	-2.38 (-15.9,-0.26)	-2.21 (-3.83,-0.47)	-1.86 (-3.64,0.15)
Electrostimulation + PFE + Bladder training	[97]	-2.3 (-4.23,-1.33)	-2.09 (-3.64,-1.18)	-2.13 (-3.07,-0.88)	-2.05 (-3.02,-1.13)
Solifenacin/trospium + placebo injection	[100]	-1.93 (-12.38,6.14)	-1.91 (-11.17,-0.96)	-1.91 (-2.93,-0.94)	-1.92 (-2.93,-1.07)
OnaBoNT-A 100u bladder base + trigone	[79]	-1.96 (-3.15,-0.72)	-1.88 (-3,-0.67)	-1.96 (-3.02,-1.02)	-1.99 (-3.1,-1.19)
OnaBoNT-A 100u bladder body + trigone	[78]	-1.83 (-2.66,-0.65)	-1.74 (-2.68,-0.97)	-1.85 (-2.55,-1.04)	-1.86 (-2.57,-1.05)
OnaBoNTA 100u trigone sparing	[72]	-1.72 (-2.09,-1.23)	-1.7 (-2.04,-1.32)	-1.75 (-2.14,-1.4)	-1.77 (-2.1,-1.42)
Tolerodine ER 4mg q.d + Neurostimulation	[96]	-1.61 (-2.4,-1.12)	-1.58 (-2.36,-1.16)	-1.6 (-2.03,-1.14)	-1.56 (-2.03,-1.13)
Estriol 1mg intravesival	[131]	-1.48 (-2.79,-0.36)	-1.65 (-3.09,-0.54)	-1.34 (-2.58,-0.29)	-1.45 (-2.72,-0.25)
Trospium IR 15mg t.i.d + Physiotherapy	[91]	-1.29 (-2.24,2.5)	-1.14 (-2.28,1.74)	-1.26 (-2.13,-0.33)	-1.33 (-2.18,-0.46)
Estradiol 3mg intravaginally	[128]	-1.38 (-3.31,0.15)	-1.19 (-3.15,0.9)	-1.05 (-2.87,0.47)	-1.35 (-3.17,-0.03)
Solifenacin ER 10mg q.d	[30]	-1.11 (-1.35,-0.9)	-1.11 (-1.33,-0.9)	-1.1 (-1.31,-0.89)	-1.1 (-1.33,-0.88)
Tolterodine ER 2mg b.i.d + Oestrogen 0.625mg 2xwk	[99]	-0.96 (-5.9,6)	-0.97 (-1.51,20.96)	-0.92 (-1.47,-0.4)	-0.96 (-1.49,-0.39)
Tolterodine ER 4mg q.d + Behaviour therapy	[87]	-1 (-13.65,-0.32)	-0.93 (-16.8,-0.22)	-0.91 (-1.6,-0.33)	-0.93 (-1.57,-0.29)
Pregabalin 150mg b.i.d + Tolterodine ER 4mg q.d	[102]	-0.97 (-1.69,-0.33)	-1.1 (-1.66,-0.45)	-0.96 (-1.66,-0.35)	-0.96 (-1.66,-0.33)
Fesoterodine ER 8mg q.d	[26]	-0.93 (-1.14,-0.73)	-0.91 (-1.11,-0.73)	-0.92 (-1.11,-0.74)	-0.92 (-1.1,-0.73)
Imidafenacin IR 0.25mg b.i.d	[37]	-1.02 (-1.74,-0.43)	-0.99 (-1.69,-0.44)	-0.98 (-1.5,-0.47)	-0.97 (-1.59,-0.45)
Solifenacin ER (5mg-10mg) q.d	[31]	-0.84 (-1.07,-0.56)	-0.85 (-1.07,-0.61)	-0.86 (-1.09,-0.64)	-0.85 (-1.07,-0.62)
Solifenacin ER 5mg - 15mg q.d	[34]	-0.93 (-20.61,-0.45)	-0.84 (-1.3,4.3)	-0.87 (-1.28,-0.45)	-0.87 (-1.26,-0.43)
Mirabegron 100mg b.i.d	[48]	-0.88 (-1.62,-0.56)	-0.87 (-1.44,-0.59)	-0.85 (-1.25,-0.6)	-0.86 (-1.3,-0.6)
Solabegron 125mg b.i.d	[55]	-0.87 (-1.18,-0.57)	-0.86 (-1.13,-0.59)	-0.85 (-1.11,-0.57)	-0.86 (-1.14,-0.57)
Propiverine ER 30mg q.d	[42]	-0.75 (-2.14,10.02)	-0.78 (-1.37,11.6)	-0.77 (-1.34,-0.44)	-0.78 (-1.4,-0.4)
Darifenacin ER 30mg q.d	[38]	-0.9 (-16.62,-0.26)	-0.87 (-14.62,-0.32)	-0.81 (-1.44,-0.28)	-0.87 (-1.5,-0.31)
Mirabegron 25mg q.d	[50]	-0.83 (-1.07,-0.57)	-0.83 (-1.06,-0.59)	-0.84 (-1.07,-0.63)	-0.83 (-1.08,-0.61)
Mirabegron 150mg b.i.d	[49]	-0.86 (-1.63,-0.49)	-0.87 (-1.53,-0.56)	-0.84 (-1.21,-0.56)	-0.85 (-1.26,-0.56)
Mirabegron 100mg q.d	[52]	-0.78 (-0.96,-0.56)	-0.78 (-0.95,-0.56)	-0.79 (-0.97,-0.61)	-0.79 (-0.97,-0.61)
Mirabegron 200mg q.d	[53]	-0.78 (-1.13,11.62)	-0.81 (-1.11,19.81)	-0.82 (-1.16,-0.51)	-0.82 (-1.14,-0.51)
Oxybutynin ER 10mg q.d	[8]	-0.9 (-1.69,-0.48)	-0.86 (-1.53,-0.49)	-0.83 (-1.35,-0.47)	-0.85 (-1.3,-0.48)
Propiverine 30mg b.i.d	[42]	-0.88 (-3.05,0)	-1 (-2.71,-0.31)	-0.8 (-1.97,-0.32)	-0.88 (-2.24,-0.33)
Oxybutynin IR 3mg t.i.d	[19]	-0.8 (-1.25,-0.43)	-0.77 (-1.19,-0.47)	-0.78 (-1.16,-0.47)	-0.79 (-1.21,-0.48)
Mirabegron 50mg q.d	[51]	-0.82 (-0.99,-0.64)	-0.82 (-0.98,-0.65)	-0.82 (-0.99,-0.66)	-0.82 (-0.99,-0.66)
Solifenacin ER 5mg q.d	[29]	-0.75 (-0.95,-0.57)	-0.76 (-0.94,-0.58)	-0.75 (-0.93,-0.58)	-0.75 (-0.93,-0.57)
Tolterodine IR 2mg b.i.d + BT	[93]	-0.85 (-2.22,-0.25)	-0.81 (-2.16,-0.17)	-0.76 (-1.29,-0.27)	-0.79 (-1.33,-0.28)
Cizolirtine Citrate 400mg b.i.d	[57]	-0.82 (-2.55,-0.14)	-0.86 (-2.12,-0.29)	-0.73 (-1.34,-0.21)	-0.78 (-1.32,-0.2)
Trospium ER 60mg q.d	[44]	-0.77 (-1.13,-0.38)	-0.74 (-1.11,-0.42)	-0.76 (-1.12,-0.41)	-0.76 (-1.1,-0.41)
Propiverine 45mg t.i.d	[118]	-0.66 (-2.01,0.78)	-0.67 (-1.92,0.37)	-0.69 (-1.46,-0.02)	-0.7 (-1.65,0.18)
Tolterodine IR 2mg b.i.d + Pilocarpine 9mg b.i.d	[101]	-0.73 (-1.15,-0.36)	-0.73 (-1.14,-0.4)	-0.73 (-1.04,-0.41)	-0.73 (-1.07,-0.41)
Fesoterodine ER 4mg q.d	[25]	-0.71 (-0.87,-0.53)	-0.71 (-0.87,-0.55)	-0.7 (-0.86,-0.55)	-0.71 (-0.86,-0.55)
Pregabalin 150mg b.i.d	[62]	-0.75 (-1.26,-0.23)	-0.76 (-1.24,-0.25)	-0.71 (-1.27,-0.23)	-0.73 (-1.3,-0.18)
Darifenacin ER 15mg q.d	[40]	-0.67 (-1.23,-0.05)	-0.7 (-1.15,-0.2)	-0.71 (-1.14,-0.25)	-0.7 (-1.15,-0.25)
Propiverine IR 15mg b.i.d	[43]	-0.62 (-1.02,0.32)	-0.69 (-1.06,-0.11)	-0.66 (-1.04,-0.3)	-0.67 (-1.06,-0.27)
Tolterodine ER 4mg q.d	[4]	-0.63 (-0.74,-0.5)	-0.64 (-0.74,-0.52)	-0.63 (-0.73,-0.53)	-0.63 (-0.73,-0.52)
Oxybutynin IR 5mg t.i.d	[7]	-0.65 (-0.98,-0.33)	-0.68 (-0.99,-0.37)	-0.66 (-0.95,-0.39)	-0.66 (-0.95,-0.4)
Propiverine ER 20mg q.d	[41]	-0.67 (-0.88,-0.48)	-0.67 (-0.86,-0.49)	-0.66 (-0.85,-0.49)	-0.67 (-0.85,-0.5)
Tolterodine IR 2mg b.i.d	[5]	-0.67 (-0.83,-0.52)	-0.66 (-0.82,-0.53)	-0.66 (-0.81,-0.53)	-0.67 (-0.81,-0.52)
Propiverine 60mg q.d	[119]	-0.49 (-1.29,1.75)	-0.64 (-1.35,1.11)	-0.61 (-1.27,0.7)	-0.59 (-1.2,0.54)
Oxybutynin intravesically 5mg t.i.d	[14]	-0.69 (-3.27,-0.03)	-0.64 (-2.05,-0.09)	-0.66 (-1.26,-0.21)	-0.64 (-1.21,-0.22)
Oxybutynin IR (2.5-5mg) b.i.d	[24]	-0.63 (-1.05,-0.22)	-0.62 (-1.02,-0.24)	-0.64 (-1.04,-0.25)	-0.62 (-0.98,-0.28)
Oxybutynin chloride topical gel 1g q.d	[13]	-0.65 (-1.07,-0.3)	-0.64 (-1,-0.32)	-0.63 (-0.95,-0.31)	-0.63 (-0.95,-0.33)
Oxybutynin vaginal ring 6mg q.d	[17]	-0.69 (-1.49,-0.28)	-0.68 (-1.24,-0.34)	-0.65 (-1.06,-0.28)	-0.65 (-1.04,-0.29)
Tolterodine IR 2mg b.i.d + PFE	[95]	-0.72 (-21.4,-0.01)	-0.58 (-1.36,18.18)	-0.62 (-1.27,0.12)	-0.65 (-1.28,0.05)
PFMT + BT	[89]	-0.78 (-2.92,-0.16)	-0.7 (-2.35,-0.1)	-0.63 (-1.26,0.02)	-0.64 (-1.22,-0.05)
Tolterodine IR 1mg b.i.d	[6]	-0.63 (-0.92,-0.29)	-0.62 (-0.9,-0.32)	-0.63 (-0.87,-0.26)	-0.62 (-0.88,-0.33)
Fesoterodine ER (4mg-8mg) q.d	[27]	-0.67 (-0.87,-0.46)	-0.66 (-0.86,-0.47)	-0.65 (-0.85,-0.46)	-0.66 (-0.85,-0.46)
Oxybutynin gel 84mg/day	[134]	-0.64 (-1.18,-0.22)	-0.61 (-1.06,-0.25)	-0.62 (-0.98,-0.25)	-0.63 (-0.97,-0.26)
Oxybutynin transdermal 3.9mg/day	[10]	-0.6 (-0.9,-0.29)	-0.59 (-0.89,-0.32)	-0.61 (-0.85,-0.36)	-0.61 (-0.87,-0.35)

Table 2: Sensitivity analyses assessing the impact in change from baseline in voiding episodes for different choices of prior distribution on variance parameters for multivariate hierarchical network meta-analysis (cont.)

Oxybutynin vaginal ring 4mg q.d	[16]	-0.59 (-1.15,-0.15)	-0.58 (-1.04,-0.2)	-0.59 (-0.94,-0.24)	-0.58 (-0.93,-0.2)
Imidafenacin IR 0.1mg b.i.d	[36]	-0.56 (-0.83,-0.29)	-0.55 (-0.83,-0.28)	-0.56 (-0.81,-0.32)	-0.56 (-0.81,-0.32)
Terodiline IR 25mg b.i.d	[28]	-0.5 (-0.97,-0.03)	-0.51 (-1.03,-0.05)	-0.54 (-0.93,-0.13)	-0.53 (-0.95,-0.09)
Darifenacin ER 7.5mg q.d	[39]	-0.58 (-1.44,0.23)	-0.61 (-1.14,0.13)	-0.58 (-1.05,0.03)	-0.61 (-1.15,-0.1)
Oxybutynin gel 56mg/day	[135]	-0.52 (-0.92,0.07)	-0.52 (-0.87,0.03)	-0.53 (-0.89,-0.15)	-0.53 (-0.88,-0.13)
Oxbutynin patch 73.5mg	[15]	-0.6 (-19.79,-0.16)	-0.54 (-0.85,9.54)	-0.56 (-0.86,-0.2)	-0.56 (-0.88,-0.24)
Elocalcitol 75mg	[70]	-0.44 (-1.05,0.37)	-0.54 (-1.06,0.22)	-0.48 (-1.05,0.05)	-0.54 (-1.09,0)
Oxybutynin 20mg intravesically q.d	[106]	-0.55 (-1.34,0.44)	-0.51 (-1.12,0.26)	-0.55 (-0.96,-0.07)	-0.58 (-1.1,-0.07)
Imidafenacin IR 0.05mg b.i.d	[35]	-0.59 (-1.14,-0.1)	-0.54 (-1,-0.14)	-0.56 (-0.98,-0.1)	-0.58 (-1.01,-0.13)
Oxybutynin ER 15mg q.d	[9]	-0.49 (-0.96,0.61)	-0.49 (-0.91,0.28)	-0.52 (-0.9,-0.01)	-0.5 (-0.89,-0.02)
Oxybutynin IR (5-20mg)	[23]	-0.56 (-6.21,6)	-0.49 (-0.92,13.33)	-0.54 (-0.94,0.09)	-0.53 (-0.97,0.05)
Oxybutynin ER (5-30mg) q.d	[22]	-0.5 (-0.93,0.5)	-0.47 (-0.91,0.27)	-0.49 (-0.84,-0.02)	-0.51 (-0.84,-0.04)
Trospium chloride 45mg t.i.d	[47]	-0.53 (-1.06,0.07)	-0.49 (-0.99,0.02)	-0.51 (-1,0.04)	-0.51 (-1,0)
Cizolirtine citrate 200mg b.i.d	[56]	-0.44 (-1.41,0.81)	-0.58 (-1.44,0.86)	-0.45 (-1.49,0.71)	-0.45 (-1.39,0.63)
PFMT	[84]	-0.53 (-1.14,0.62)	-0.43 (-1.12,0.3)	-0.48 (-1.11,0.13)	-0.52 (-1.12,0.1)
Oxybutynin transdermal 1.3mg/day	[11]	-0.45 (-0.83,0.24)	-0.48 (-0.8,0.13)	-0.45 (-0.8,-0.01)	-0.48 (-0.81,-0.04)
Elocalcitol 150mg	[69]	-0.46 (-1.09,0.3)	-0.52 (-1.06,0.12)	-0.46 (-1.04,0.08)	-0.54 (-1.09,0.01)
Oxybutynin ER 2.5mg q.d	[20]	-0.54 (-1.73,0.11)	-0.52 (-1.7,-0.02)	-0.52 (-0.92,0.02)	-0.5 (-0.89,0.06)
Duloxetine IR 40mg b.i.d	[65]	-0.57 (-18.72,0.04)	-0.53 (-8.7,0.06)	-0.45 (-1.05,0.11)	-0.45 (-1.03,0.08)
Bladder Training	[85]	-0.5 (-0.96,0.17)	-0.45 (-0.97,0.09)	-0.45 (-0.86,-0.02)	-0.47 (-0.87,-0.02)
Solabegron 50mg b.i.d	[54]	-0.49 (-0.82,-0.19)	-0.5 (-0.75,-0.21)	-0.48 (-0.76,-0.2)	-0.48 (-0.75,-0.19)
Oxybutynin IR 2.5mg t.i.d	[21]	-0.6 (-2.49,-0.21)	-0.57 (-2.28,-0.2)	-0.52 (-0.92,-0.15)	-0.54 (-0.92,-0.13)
Oxybutynin transdermal 2.6mg/day	[12]	-0.39 (-0.76,0.26)	-0.41 (-0.76,0.17)	-0.41 (-0.76,0.11)	-0.42 (-0.76,0.09)
Pregabalin 75mg b.i.d + Tolterodine ER 2mg q.d	[103]	-0.45 (-1.01,0)	-0.43 (-0.94,-0.01)	-0.45 (-0.91,-0.03)	-0.46 (-0.93,0.07)
Oxybutynin ER 2.5mg q.d + Bladder training	[92]	-0.48 (-2.07,0.44)	-0.49 (-1.73,0.65)	-0.5 (-1.57,0.34)	-0.37 (-1.29,0.61)
Oxybutynin IR 5mg b.i.d	[18]	-0.36 (-0.81,0.7)	-0.33 (-0.81,0.48)	-0.38 (-0.76,0.18)	-0.39 (-0.77,0.2)
Lipo-BoNTA	[138]	-0.5 (-2.43,0.49)	-0.44 (-2.07,0.6)	-0.39 (-1.25,0.38)	-0.42 (-1.26,0.52)
Serlopitant 0.25mg q.d	[107]	-0.35 (-0.81,0.12)	-0.41 (-0.82,0.06)	-0.31 (-0.8,0.1)	-0.37 (-0.87,0.11)
Serlopitant 4mg q.d	[109]	-0.36 (-0.83,0.09)	-0.38 (-0.83,0.1)	-0.33 (-0.84,0.09)	-0.36 (-0.87,0.1)
Tarafenacin 0.4mg q.d	[82]	-0.47 (-1.38,0.17)	-0.48 (-1.32,0.14)	-0.43 (-1.04,0.16)	-0.37 (-0.99,0.2)
Electrostimulation + vaginal oestrogen cream 1.25mg/day	[133]	-0.35 (-1.59,0.47)	-0.32 (-1.59,0.42)	-0.27 (-0.93,0.46)	-0.26 (-0.94,0.52)
Electrostimulation	[80]	-0.42 (-1.49,0.1)	-0.4 (-1.53,0.08)	-0.35 (-0.78,0.12)	-0.35 (-0.82,0.18)
Serlopitant 1mg q.d	[108]	-0.18 (-0.64,0.39)	-0.21 (-0.61,0.27)	-0.15 (-0.62,0.39)	-0.2 (-0.67,0.31)
Estradiol 25mg	[68]	-0.29 (-26.23,0.22)	-0.22 (-23.51,0.26)	-0.22 (-0.67,0.25)	-0.22 (-0.67,0.21)
Placebo	[1]	NA	NA	NA	NA
Netupitant 200mg q.d	[112]	-0.18 (-1.24,0.8)	-0.08 (-1.06,1.05)	-0.17 (-1.12,0.89)	-0.28 (-1.33,0.73)
Netupitant 100mg q.d	[111]	-0.09 (-1.14,0.91)	-0.03 (-1.08,0.91)	-0.1 (-1.08,0.82)	-0.27 (-1.31,0.79)
Tarafenacin 0.2mg q.d	[90]	-0.16 (-0.93,0.53)	-0.18 (-0.79,0.5)	-0.22 (-0.84,0.44)	-0.12 (-0.71,0.49)
Trospium IR 15mg t.i.d	[46]	-0.04 (-0.88,6.44)	-0.07 (-0.83,7.02)	-0.08 (-0.8,0.97)	-0.14 (-0.84,0.73)
ZD0947IL 25mg/day	[58]	0 (-0.88,1.65)	-0.08 (-1,1.09)	-0.08 (-0.88,0.8)	-0.04 (-0.86,0.93)
Netupitant 50mg q.d	[110]	-0.03 (-1.08,0.96)	-0.05 (-0.96,0.99)	-0.1 (-1.08,1.04)	-0.18 (-1.14,0.88)
Electromagnetic stimulation	[125]	-0.42 (-2.34,1.35)	-0.23 (-2.3,1.44)	-0.5 (-2.05,1.28)	-0.61 (-2.09,1.24)
Oxybutynin ER 5-30mg/day + Behaviour therapy	[22]	0.01 (-1.33,1.29)	0.15 (-1.08,1.33)	0.15 (-0.86,1.04)	0.05 (-0.87,1.11)
ONO-8539 100mg b.i.d	[60]	-0.03 (-0.76,0.6)	-0.05 (-0.69,0.59)	-0.09 (-0.79,0.61)	-0.07 (-0.71,0.52)
Percutaneous tibial nerve stimulation	[83]	-0.31 (-1.29,0.61)	-0.2 (-1.32,0.6)	-0.22 (-1.1,0.66)	-0.18 (-1.18,0.68)
Vaginal oestrogen cream 1.25mg/day	[132]	-0.07 (-1.1,0.54)	-0.09 (-1.13,0.46)	-0.01 (-0.53,0.5)	0 (-0.6,0.55)
Flavoxate chloride 200mg q.d	[64]	0.16 (-8.81,13.16)	0.18 (-0.65,59.98)	0.15 (-0.57,0.89)	0.14 (-0.61,0.85)
Resiniferatoxin 50nM	[67]	0.12 (-1.03,1.68)	0.2 (-1.52,1.14)	0.21 (-0.82,1.4)	0.14 (-0.86,1.45)
Emepronium bromide 200mg q.d	[63]	0.29 (-0.48,31.41)	0.21 (-0.6,42.29)	0.14 (-0.57,1.01)	0.14 (-0.6,0.87)
ONO-8539 300mg b.i.d	[61]	0.24 (-0.41,0.83)	0.24 (-0.42,0.74)	0.19 (-0.39,0.77)	0.2 (-0.37,0.78)
Propantheline Bromide 15mg t.i.d	[113]	0.28 (-0.71,1.28)	0.21 (-0.94,1.32)	0.41 (-0.74,1.7)	0.27 (-0.8,1.4)
Estradiol 1mg intravaginally	[127]	0.45 (-1.24,2.12)	0.28 (-0.8,1.78)	0.39 (-0.94,1.7)	0.3 (-1.1,1.68)
ONO-8539 30mg b.i.d	[59]	0.3 (-0.31,0.97)	0.24 (-0.3,0.86)	0.29 (-0.35,0.86)	0.28 (-0.32,0.88)
Control	[2]	0.43 (-0.37,2.26)	0.54 (-0.38,1.5)	0.43 (-0.36,1.13)	0.43 (-0.48,1.15)
Reflexology	[71]	0.24 (-1.01,1.49)	0.38 (-0.89,1.36)	0.37 (-0.51,1.17)	0.37 (-0.59,1.18)
Sham Therapy	[3]	0.46 (-0.57,1.53)	0.48 (-0.55,1.52)	0.49 (-0.51,1.45)	0.53 (-0.48,1.45)
Naftopidil 25mg q.d	[114]	3.36 (0.71,6.49)	3.59 (1.16,5.47)	3.33 (0.9,5.81)	3.64 (2,5.54)
Solifenacin succinate 5mg q.d + Naftopidil 25mg q.d	[115]	5.71 (3.19,8.53)	5.63 (3.29,8.72)	4.93 (2.67,7.3)	5.73 (3.91,8.03)

† Elements of $\boldsymbol{V}^{1/2}$ based on a Gamma(0.001,0.001) prior distribution on the precision scale
†† Elements of $\boldsymbol{V}^{1/2}$ based on a Half-Normal(0,1)I(0,) prior distribution on the standard deviation scale
§ Deviance of treatment effect profiles across outcomes, τ, based on a Gamma(0.01,0.01) prior distribution on the precision scale
§§ Deviance of treatment effect profiles across outcomes, τ, based on a Half-Normal(0,1)I(0,) prior distribution on the standard deviation scale

Table 3: Sensitivity analyses assessing the impact in change from baseline in urgency episodes for different choices of prior distribution on variance parameters for multivariate hierarchical network meta-analysis

**Treatment**	**Code**	**Median difference† (95%CrI)**	**Median difference†† (95%CrI)**	**Median difference§ (95%CrI)**	**Median difference§§ (95%CrI)**
Sacral nerve stimulation	[81]	-9.25 (-17.35,-4.97)	-8.35 (-10.22,3.64)	-9.05 (-11.04,-6.31)	-8.46 (-10.95,-6.07)
OnaBoNT-A 200u trigone sparing	[73]	-2.52 (-3.52,-1.14)	-2.5 (-3.53,-1.48)	-2.49 (-3.7,-1.85)	-2.55 (-3.5,-1.82)
Oxybutynin IR 2.5mg b.i.d + Salivary pastilles	[98]	-2.17 (-6.43,23.36)	-2.58 (-6.49,3.42)	-2.55 (-4.21,-0.79)	-2.15 (-3.99,-0.29)
Electrostimulation + PFE + Bladder training	[97]	-2.65 (-56.88,-1.63)	-2.39 (-15.44,-0.1)	-2.45 (-3.4,-1.25)	-2.39 (-3.34,-1.4)
Solifenacin/trospium + placebo injection	[100]	-2.19 (-5.89,49.25)	-2.22 (-15.6,1.93)	-2.22 (-3.28,-1.26)	-2.22 (-3.27,-1.35)
OnaBoNT-A 100u bladder base + trigone	[79]	-2.32 (-3.98,-1.05)	-2.22 (-3.67,-1.04)	-2.27 (-3.36,-1.34)	-2.3 (-3.41,-1.49)
OnaBoNT-A 100u bladder body + trigone	[78]	-2.23 (-3.9,-1.18)	-2.13 (-3.47,-1.36)	-2.16 (-2.96,-1.37)	-2.17 (-2.9,-1.34)
OnaBoNTA 100u trigone sparing	[72]	-2.14 (-2.74,-1.47)	-2.08 (-2.61,-1.61)	-2.12 (-2.6,-1.69)	-2.13 (-2.59,-1.71)
Tolerodine ER 4mg q.d + Neurostimulation	[96]	-1.92 (-3.4,-1.32)	-1.92 (-3.08,-1.42)	-1.91 (-2.4,-1.39)	-1.86 (-2.38,-1.36)
Estriol 1mg intravesival	[131]	-1.92 (-52.53,3.51)	-2.07 (-19.29,-0.75)	-1.66 (-2.96,-0.58)	-1.76 (-3.07,-0.48)
Trospium IR 15mg t.i.d + Physiotherapy	[91]	-1.82 (-20.29,-0.63)	-1.54 (-4.23,7.33)	-1.56 (-2.46,-0.58)	-1.61 (-2.52,-0.72)
Estradiol 3mg intravaginally	[128]	-1.76 (-30.65,-0.09)	-1.42 (-3.42,2.6)	-1.39 (-3.19,0.26)	-1.68 (-3.53,-0.3)
Solifenacin ER 10mg q.d	[30]	-1.37 (-1.84,-0.9)	-1.41 (-1.82,-0.98)	-1.38 (-1.73,-1.01)	-1.36 (-1.76,-1)
Tolterodine ER 2mg b.i.d + Oestrogen 0.625mg 2xwk	[99]	-1.25 (-6.62,10.38)	-1.31 (-2.01,15)	-1.23 (-1.84,-0.66)	-1.28 (-1.84,-0.62)
Tolterodine ER 4mg q.d + Behaviour therapy	[87]	-1.16 (-3.88,34.4)	-1.22 (-5.55,2.71)	-1.24 (-1.92,-0.59)	-1.23 (-1.94,-0.56)
Pregabalin 150mg b.i.d + Tolterodine ER 4mg q.d	[102]	-1.19 (-2.04,0.06)	-1.4 (-2.03,-0.26)	-1.25 (-2.09,-0.57)	-1.24 (-2.04,-0.57)
Fesoterodine ER 8mg q.d	[26]	-1.26 (-1.83,-0.89)	-1.25 (-1.7,-0.93)	-1.24 (-1.58,-0.88)	-1.22 (-1.56,-0.89)
Imidafenacin IR 0.25mg b.i.d	[37]	-1.29 (-2.41,-0.48)	-1.28 (-2.29,-0.6)	-1.28 (-1.88,-0.67)	-1.28 (-1.92,-0.65)
Solifenacin ER (5mg-10mg) q.d	[31]	-1.16 (-1.58,-0.49)	-1.18 (-1.58,-0.58)	-1.2 (-1.54,-0.81)	-1.17 (-1.53,-0.83)
Solifenacin ER 5mg - 15mg q.d	[34]	-1.19 (-19.35,15.31)	-1.21 (-8.54,1.97)	-1.19 (-1.67,-0.71)	-1.16 (-1.63,-0.69)
Mirabegron 100mg b.i.d	[48]	-1.17 (-2.14,-0.32)	-1.19 (-1.74,-0.58)	-1.17 (-1.62,-0.79)	-1.16 (-1.66,-0.8)
Solabegron 125mg b.i.d	[55]	-1.21 (-69.74,-0.46)	-1.2 (-24.06,-0.76)	-1.16 (-1.53,-0.77)	-1.16 (-1.54,-0.75)
Propiverine ER 30mg q.d	[42]	-1.18 (-41.8,-0.48)	-1.15 (-1.78,11.39)	-1.11 (-1.7,-0.67)	-1.1 (-1.73,-0.64)
Darifenacin ER 30mg q.d	[38]	-1.16 (-12.9,3.88)	-1.15 (-3.06,11.84)	-1.12 (-1.8,-0.53)	-1.18 (-1.84,-0.59)
Mirabegron 25mg q.d	[50]	-1.09 (-1.46,0.15)	-1.13 (-1.49,0.21)	-1.14 (-1.49,-0.78)	-1.13 (-1.49,-0.78)
Mirabegron 150mg b.i.d	[49]	-1.15 (-2.27,-0.24)	-1.18 (-1.82,-0.53)	-1.15 (-1.58,-0.75)	-1.14 (-1.6,-0.77)
Mirabegron 100mg q.d	[52]	-1.1 (-1.44,-0.37)	-1.12 (-1.45,-0.48)	-1.12 (-1.45,-0.78)	-1.12 (-1.45,-0.79)
Mirabegron 200mg q.d	[53]	-1.11 (-5.29,30.81)	-1.17 (-10.55,3.46)	-1.14 (-1.53,-0.72)	-1.13 (-1.53,-0.74)
Oxybutynin ER 10mg q.d	[8]	-1.18 (-27.39,2.21)	-1.13 (-2.09,4.24)	-1.14 (-1.67,-0.66)	-1.14 (-1.65,-0.67)
Propiverine 30mg b.i.d	[42]	-1.17 (-14.27,4.51)	-1.34 (-5.3,1.16)	-1.14 (-2.36,-0.53)	-1.18 (-2.55,-0.58)
Oxybutynin IR 3mg t.i.d	[19]	-1.04 (-1.56,32.3)	-1.08 (-1.61,6.44)	-1.1 (-1.58,-0.69)	-1.1 (-1.62,-0.69)
Mirabegron 50mg q.d	[51]	-1.08 (-1.41,-0.4)	-1.11 (-1.44,-0.38)	-1.12 (-1.44,-0.78)	-1.11 (-1.44,-0.79)
Solifenacin ER 5mg q.d	[29]	-1.13 (-1.61,-0.75)	-1.14 (-1.53,-0.79)	-1.11 (-1.46,-0.76)	-1.11 (-1.45,-0.77)
Tolterodine IR 2mg b.i.d + BT	[93]	-1.09 (-2.05,-0.06)	-1.09 (-2.02,-0.25)	-1.06 (-1.64,-0.49)	-1.08 (-1.65,-0.49)
Cizolirtine Citrate 400mg b.i.d	[57]	-1.16 (-4.01,-0.51)	-1.2 (-3.28,-0.6)	-1.04 (-1.71,-0.45)	-1.08 (-1.7,-0.46)
Trospium ER 60mg q.d	[44]	-1.01 (-1.5,17.75)	-1.05 (-1.54,14.01)	-1.08 (-1.52,-0.61)	-1.07 (-1.5,-0.63)
Propiverine 45mg t.i.d	[118]	-0.97 (-22.29,10.74)	-0.98 (-2.61,4.56)	-1.01 (-1.8,-0.28)	-1.01 (-2.01,-0.07)
Tolterodine IR 2mg b.i.d + Pilocarpine 9mg b.i.d	[101]	-1.06 (-78.09,4.47)	-1.04 (-13.41,6.74)	-1.05 (-1.45,-0.6)	-1.03 (-1.46,-0.61)
Fesoterodine ER 4mg q.d	[25]	-1.04 (-1.43,-0.67)	-1.05 (-1.43,-0.73)	-1.03 (-1.34,-0.7)	-1.02 (-1.33,-0.7)
Pregabalin 150mg b.i.d	[62]	-1.02 (-1.72,-0.04)	-1.07 (-1.68,-0.3)	-1.01 (-1.69,-0.44)	-1.02 (-1.66,-0.38)
Darifenacin ER 15mg q.d	[40]	-0.97 (-1.62,0.42)	-1 (-1.63,-0.06)	-1.03 (-1.55,-0.48)	-1.01 (-1.53,-0.49)
Propiverine IR 15mg b.i.d	[43]	-0.99 (-2.26,0.06)	-1.05 (-1.65,-0.33)	-1 (-1.45,-0.57)	-1 (-1.46,-0.54)
Tolterodine ER 4mg q.d	[4]	-0.98 (-1.28,-0.67)	-1 (-1.3,-0.72)	-0.98 (-1.27,-0.67)	-0.97 (-1.26,-0.69)
Oxybutynin IR 5mg t.i.d	[7]	-1.03 (-20.9,-0.61)	-1 (-1.46,6.64)	-0.98 (-1.38,-0.58)	-0.98 (-1.37,-0.59)
Propiverine ER 20mg q.d	[41]	-0.97 (-1.46,-0.52)	-1 (-1.38,-0.61)	-0.97 (-1.3,-0.62)	-0.97 (-1.31,-0.63)
Tolterodine IR 2mg b.i.d	[5]	-0.96 (-1.44,-0.38)	-0.98 (-1.36,-0.51)	-0.97 (-1.27,-0.63)	-0.96 (-1.27,-0.63)
Propiverine 60mg q.d	[119]	-0.86 (-16.98,10.28)	-0.98 (-2.65,5.84)	-0.91 (-1.6,0.42)	-0.9 (-1.53,0.25)
Oxybutynin intravesically 5mg t.i.d	[14]	-0.99 (-35.24,7.33)	-0.98 (-6.74,1.16)	-0.99 (-1.63,-0.45)	-0.96 (-1.62,-0.44)
Oxybutynin IR (2.5-5mg) b.i.d	[24]	-1 (-23.39,-0.49)	-0.96 (-29.88,-0.56)	-0.98 (-1.43,-0.44)	-0.94 (-1.38,-0.52)
Oxybutynin chloride topical gel 1g q.d	[13]	-0.92 (-6.17,34.22)	-0.94 (-1.46,14.52)	-0.94 (-1.35,-0.52)	-0.94 (-1.34,-0.54)
Oxybutynin vaginal ring 6mg q.d	[17]	-0.91 (-1.81,19.92)	-0.96 (-1.54,5.97)	-0.96 (-1.45,-0.52)	-0.94 (-1.4,-0.52)
Tolterodine IR 2mg b.i.d + PFE	[95]	-0.92 (-1.76,0.29)	-0.95 (-1.78,0.15)	-0.93 (-1.65,-0.09)	-0.96 (-1.64,-0.22)
PFMT + BT	[89]	-0.92 (-1.57,99.58)	-0.95 (-2.53,3.14)	-0.93 (-1.57,-0.29)	-0.92 (-1.55,-0.28)
Tolterodine IR 1mg b.i.d	[6]	-0.99 (-57.01,-0.54)	-0.95 (-12.77,-0.59)	-0.94 (-1.31,-0.44)	-0.93 (-1.29,-0.52)
Fesoterodine ER (4mg-8mg) q.d	[27]	-0.97 (-1.53,-0.55)	-0.97 (-1.4,-0.6)	-0.95 (-1.3,-0.6)	-0.95 (-1.29,-0.6)
Oxybutynin gel 84mg/day	[134]	-0.87 (-1.38,23.7)	-0.88 (-1.41,23.04)	-0.92 (-1.36,-0.44)	-0.91 (-1.34,-0.48)
Oxybutynin transdermal 3.9mg/day	[10]	-0.9 (-1.47,0.07)	-0.92 (-1.5,-0.32)	-0.92 (-1.27,-0.53)	-0.91 (-1.28,-0.54)

Table 3: Sensitivity analyses assessing the impact in change from baseline in urgency episodes for different choices of prior distribution on variance parameters for multivariate hierarchical network meta-analysis (cont.)

Oxybutynin vaginal ring 4mg q.d	[16]	-0.92 (-22.22,4.6)	-0.93 (-10.01,1.22)	-0.91 (-1.34,-0.45)	-0.89 (-1.34,-0.43)
Imidafenacin IR 0.1mg b.i.d	[36]	-0.87 (-1.35,-0.22)	-0.89 (-1.36,-0.39)	-0.9 (-1.27,-0.49)	-0.89 (-1.26,-0.51)
Terodiline IR 25mg b.i.d	[28]	-0.84 (-5.95,13.09)	-0.85 (-1.65,17.65)	-0.88 (-1.4,-0.36)	-0.87 (-1.36,-0.35)
Darifenacin ER 7.5mg q.d	[39]	-0.95 (-2.73,-0.27)	-0.97 (-2.15,-0.35)	-0.89 (-1.45,-0.28)	-0.93 (-1.52,-0.38)
Oxybutynin gel 56mg/day	[135]	-0.91 (-69.77,5.35)	-0.92 (-13.1,-0.43)	-0.87 (-1.32,-0.39)	-0.85 (-1.3,-0.38)
Oxbutynin patch 73.5mg	[15]	-0.82 (-1.25,102.6)	-0.89 (-1.34,63.1)	-0.88 (-1.26,-0.44)	-0.87 (-1.27,-0.45)
Elocalcitol 75mg	[70]	-0.84 (-1.68,0.15)	-0.93 (-1.65,-0.1)	-0.83 (-1.45,-0.23)	-0.88 (-1.48,-0.27)
Oxybutynin 20mg intravesically q.d	[106]	-0.83 (-2.43,13.33)	-0.88 (-7.58,4.12)	-0.87 (-1.37,-0.34)	-0.88 (-1.49,-0.31)
Imidafenacin IR 0.05mg b.i.d	[35]	-0.86 (-1.59,0.25)	-0.84 (-1.45,-0.03)	-0.87 (-1.37,-0.36)	-0.88 (-1.38,-0.36)
Oxybutynin ER 15mg q.d	[9]	-0.79 (-1.47,55.46)	-0.84 (-2.25,6.25)	-0.84 (-1.3,-0.28)	-0.81 (-1.29,-0.27)
Oxybutynin IR (5-20mg)	[23]	-0.83 (-8.22,11.46)	-0.86 (-10.63,0.54)	-0.85 (-1.34,-0.2)	-0.83 (-1.32,-0.18)
Oxybutynin ER (5-30mg) q.d	[22]	-0.77 (-1.26,47.29)	-0.81 (-1.39,12.69)	-0.82 (-1.27,-0.29)	-0.83 (-1.25,-0.28)
Trospium chloride 45mg t.i.d	[47]	-0.76 (-1.41,31.11)	-0.78 (-1.38,23.06)	-0.83 (-1.4,-0.16)	-0.83 (-1.39,-0.22)
Cizolirtine citrate 200mg b.i.d	[56]	-0.81 (-1.87,0.55)	-0.97 (-1.83,0.64)	-0.75 (-1.89,0.38)	-0.75 (-1.76,0.31)
PFMT	[84]	-0.87 (-15.47,19.03)	-0.71 (-1.45,29.63)	-0.8 (-1.49,-0.1)	-0.81 (-1.49,-0.15)
Oxybutynin transdermal 1.3mg/day	[11]	-0.74 (-1.23,14.67)	-0.89 (-31.49,-0.32)	-0.77 (-1.21,-0.26)	-0.79 (-1.23,-0.28)
Elocalcitol 150mg	[69]	-0.77 (-1.59,0.51)	-0.85 (-1.5,0.12)	-0.78 (-1.4,-0.16)	-0.83 (-1.47,-0.23)
Oxybutynin ER 2.5mg q.d	[20]	-0.8 (-2.01,0.93)	-0.85 (-1.58,0.25)	-0.82 (-1.27,-0.22)	-0.78 (-1.26,-0.18)
Duloxetine IR 40mg b.i.d	[65]	-0.86 (-39.06,0.18)	-0.83 (-6,2.1)	-0.76 (-1.45,-0.15)	-0.77 (-1.39,-0.18)
Bladder Training	[85]	-0.81 (-1.57,0.32)	-0.77 (-1.52,0.08)	-0.77 (-1.26,-0.26)	-0.78 (-1.28,-0.26)
Solabegron 50mg b.i.d	[54]	-0.74 (-1.23,261.6)	-0.84 (-11.73,7.79)	-0.8 (-1.18,-0.4)	-0.79 (-1.18,-0.38)
Oxybutynin IR 2.5mg t.i.d	[21]	-0.8 (-2.09,-0.33)	-0.83 (-1.81,-0.39)	-0.75 (-1.15,-0.32)	-0.74 (-1.16,-0.34)
Oxybutynin transdermal 2.6mg/day	[12]	-0.79 (-15.02,0.44)	-0.76 (-2.27,4.03)	-0.72 (-1.17,-0.14)	-0.72 (-1.18,-0.17)
Pregabalin 75mg b.i.d + Tolterodine ER 2mg q.d	[103]	-0.71 (-1.5,0.3)	-0.74 (-1.33,0.05)	-0.76 (-1.33,-0.22)	-0.75 (-1.35,-0.16)
Oxybutynin ER 2.5mg q.d + Bladder training	[92]	-0.74 (-2.33,0.62)	-0.8 (-1.78,0.45)	-0.81 (-1.82,0.06)	-0.68 (-1.63,0.35)
Oxybutynin IR 5mg b.i.d	[18]	-0.73 (-40.86,4.7)	-0.7 (-12.26,2.47)	-0.7 (-1.17,-0.09)	-0.69 (-1.18,-0.07)
Lipo-BoNTA	[138]	-0.76 (-2.62,0.42)	-0.73 (-2.13,0.31)	-0.69 (-1.6,0.12)	-0.71 (-1.61,0.26)
Serlopitant 0.25mg q.d	[107]	-0.58 (-1.18,26.58)	-0.7 (-1.21,15.49)	-0.63 (-1.18,-0.11)	-0.68 (-1.22,-0.13)
Serlopitant 4mg q.d	[109]	-0.6 (-1.28,31.89)	-0.74 (-7.43,2.89)	-0.65 (-1.19,-0.11)	-0.67 (-1.27,-0.13)
Tarafenacin 0.4mg q.d	[82]	-0.71 (-1.71,0.5)	-0.82 (-1.61,-0.02)	-0.73 (-1.39,-0.12)	-0.68 (-1.32,-0.02)
Electrostimulation + vaginal oestrogen cream 1.25mg/day	[133]	-0.77 (-7.23,0.11)	-0.76 (-6.74,0.02)	-0.67 (-1.34,0.12)	-0.64 (-1.38,0.13)
Electrostimulation	[80]	-0.8 (-4.85,-0.18)	-0.76 (-4.48,-0.25)	-0.7 (-1.23,-0.18)	-0.7 (-1.27,-0.19)
Serlopitant 1mg q.d	[108]	-0.56 (-25.9,2.42)	-0.55 (-11.97,1.64)	-0.48 (-1.02,0.14)	-0.52 (-1.06,0.07)
Estradiol 25mg	[68]	-0.6 (-62.48,0.04)	-0.54 (-4.97,1.42)	-0.53 (-1.05,0.03)	-0.53 (-1.04,-0.03)
Placebo	[1]	NA	NA	NA	NA
Netupitant 200mg q.d	[112]	-0.41 (-1.44,0.89)	-0.4 (-1.34,0.81)	-0.47 (-1.41,0.62)	-0.58 (-1.68,0.43)
Netupitant 100mg q.d	[111]	-0.42 (-1.59,0.69)	-0.41 (-1.48,0.55)	-0.41 (-1.38,0.52)	-0.6 (-1.6,0.49)
Tarafenacin 0.2mg q.d	[90]	-0.47 (-1.38,0.64)	-0.54 (-1.31,0.6)	-0.54 (-1.22,0.14)	-0.44 (-1.07,0.23)
Trospium IR 15mg t.i.d	[46]	-0.39 (-1.26,32.98)	-0.46 (-1.91,6.04)	-0.4 (-1.21,0.69)	-0.47 (-1.24,0.42)
ZD0947IL 25mg/day	[58]	-0.4 (-1.47,1.22)	-0.46 (-1.49,0.93)	-0.41 (-1.24,0.48)	-0.37 (-1.22,0.65)
Netupitant 50mg q.d	[110]	-0.32 (-1.42,0.84)	-0.37 (-1.28,0.72)	-0.41 (-1.4,0.74)	-0.5 (-1.43,0.57)
Electromagnetic stimulation	[125]	-0.81 (-34.11,10.2)	-0.43 (-2.64,20.71)	-0.81 (-2.42,0.94)	-0.89 (-2.45,0.91)
Oxybutynin ER 5-30mg/day + Behaviour therapy	[22]	-0.37 (-39.69,1.33)	-0.16 (-5.93,1.7)	-0.17 (-1.2,0.8)	-0.25 (-1.2,0.84)
ONO-8539 100mg b.i.d	[60]	-0.31 (-1.32,0.87)	-0.39 (-1.11,0.52)	-0.4 (-1.15,0.41)	-0.38 (-1.06,0.3)
Percutaneous tibial nerve stimulation	[83]	-0.7 (-3.2,0.34)	-0.56 (-2.93,0.3)	-0.52 (-1.5,0.39)	-0.47 (-1.54,0.43)
Vaginal oestrogen cream 1.25mg/day	[132]	-0.35 (-3.68,0.36)	-0.39 (-3.36,0.26)	-0.3 (-0.84,0.34)	-0.25 (-0.9,0.34)
Flavoxate chloride 200mg q.d	[64]	-0.31 (-53.47,0.55)	-0.12 (-0.99,34.65)	-0.17 (-0.89,0.61)	-0.17 (-0.93,0.6)
Resiniferatoxin 50nM	[67]	-0.04 (-1.54,57.63)	-0.05 (-2.24,17.83)	-0.11 (-1.14,1.13)	-0.15 (-1.24,1.18)
Emepronium bromide 200mg q.d	[63]	-0.04 (-0.81,38.91)	-0.12 (-0.96,23.73)	-0.18 (-0.91,0.69)	-0.17 (-0.94,0.63)
ONO-8539 300mg b.i.d	[61]	-0.09 (-1.14,0.67)	-0.09 (-0.99,0.62)	-0.14 (-0.77,0.53)	-0.12 (-0.74,0.52)
Propantheline Bromide 15mg t.i.d	[113]	0.01 (-6.67,36.21)	-0.21 (-22.27,0.96)	0.09 (-1.06,1.42)	-0.04 (-1.12,1.13)
Estradiol 1mg intravaginally	[127]	-0.06 (-46.43,1.83)	-0.17 (-20.47,1.46)	0.06 (-1.26,1.43)	-0.01 (-1.42,1.36)
ONO-8539 30mg b.i.d	[59]	0.03 (-0.71,1.52)	-0.06 (-0.82,1.28)	-0.02 (-0.73,0.63)	-0.02 (-0.67,0.65)
Control	[2]	0.09 (-0.77,15.33)	0.12 (-6.33,1.18)	0.09 (-0.79,0.82)	0.1 (-0.9,0.86)
Reflexology	[71]	0.12 (-0.89,15.79)	0.1 (-6.05,1.45)	0.1 (-0.87,0.9)	0.09 (-0.87,0.92)
Sham Therapy	[3]	0.22 (-0.89,54.33)	0.2 (-0.9,11.57)	0.18 (-0.93,1.13)	0.21 (-0.87,1.17)
Naftopidil 25mg q.d	[114]	3.21 (-13.5,27.33)	3.36 (0.5,13.03)	2.96 (0.53,5.47)	3.33 (1.73,5.28)
Solifenacin succinate 5mg q.d + Naftopidil 25mg q.d	[115]	5.63 (-3.65,14.63)	5.46 (2.81,17.52)	4.63 (2.27,7.02)	5.41 (3.56,7.84)

† Elements of $\boldsymbol{V^{1/2}}$ based on a Gamma(0.001,0.001) prior distribution on the precision scale
†† Elements of $\boldsymbol{V^{1/2}}$ based on a Half-Normal(0,1)I(0,) prior distribution on the standard deviation scale
§ Deviance of treatment effect profiles across outcomes, τ, based on a Gamma(0.01,0.01) prior distribution on the precision scale
§§ Deviance of treatment effect profiles across outcomes, τ, based on a Half-Normal(0,1)I(0,) prior distribution on the standard deviation scale
